# Growth Promotion-Related miRNAs in *Oncidium* Orchid Roots Colonized by the Endophytic Fungus *Piriformospora indica*

**DOI:** 10.1371/journal.pone.0084920

**Published:** 2014-01-07

**Authors:** Wei Ye, Chin-Hui Shen, Yuling Lin, Peng-Jen Chen, Xuming Xu, Ralf Oelmüller, Kai-Wun Yeh, Zhongxiong Lai

**Affiliations:** 1 Institute of Horticultural Biotechnology, Fujian Agriculture and Forestry University, Fuzhou, Fujian, China; 2 Institute of Plant Biology, National Taiwan University, Taipei, Taiwan; 3 Sanming Academy of Agricultural Sciences, Sanming, Fujian, China; 4 Ecological Materials Technology Department, Green Energy & Eco-technology System Center, ITRI South Campus, Industrial Technology Research Institute, Tainan, Taiwan; 5 Department of General Botany and Plant Physiology, Friedrich-Schiller University, Jena, Germany; University of Nottingham, United Kingdom

## Abstract

*Piriformospora indica*, an endophytic fungus of Sebacinales, colonizes the roots of a wide range of host plants and establishes various benefits for the plants. In this work, we describe miRNAs which are upregulated in *Oncidium* orchid roots after colonization by the fungus. Growth promotion and vigorous root development were observed in *Oncidium* hybrid orchid, while seedlings were colonized by *P. indica*. We performed a genome-wide expression profiling of small RNAs in *Oncidium* orchid roots either colonized or not-colonized by *P. indica*. After sequencing, 24,570,250 and 24744,141 clean reads were obtained from two libraries. 13,736 from 17,036,953 unique sequences showed homology to either 86 miRNA families described in 41 plant species, or to 46 potential novel miRNAs, or to 51 corresponding miRNA precursors. The predicted target genes of these miRNAs are mainly involved in auxin signal perception and transduction, transcription, development and plant defense. The expression analysis of miRNAs and target genes demonstrated the regulatory functions they may participate in. This study revealed that growth stimulation of the *Oncidium* orchid after colonization by *P. indica* includes an intricate network of miRNAs and their targets. The symbiotic function of *P. indica* on *Oncidium* orchid resembles previous findings on Chinese cabbage. This is the first study on growth regulation and development of *Oncidium* orchid by miRNAs induced by the symbiotic fungus *P. indica*.

## Introduction

*Piriformospora indica*, a root-colonizing endophyte with a broad host range, is intensively studied due to its diverse beneficial effects on the performance of both monocot and eudicot plants. *P. indica* improves nutrition uptake of the host plants [1], [2], enhances their resistance to biotic and abiotic stress [3]–[6], promotes the biomass of the aerial and under-ground parts [7] as well as the accumulation of secondary metabolites followed by a cell death-dependent phase, and this lifestyle is critical for local and systemic resistance induced by *P. indica* [6], [8]–[11]. Since the fungus can easily be cultivated without any host, it became a model organism to study symbiotic root interactions [3]. Proteomics and transcriptomics were employed to study the interaction of *P. indica* with *Arabidopsis thaliana* and barley, and stage-specific up- or down-regulated proteins and genes involved in phytohormone metabolism [11], [12] and membrane protein synthesis [13] were detected. *P. indica* also promotes growth of Chinese cabbage (*Brassica campestris* subsp.*chinensis*). Analysis of a double-subtracted EST cDNA library identified genes associated with auxin biosynthesis and signaling indicating that this phytohormone might be crucial in the Chinese cabbage/*P. indica* symbiosis [14].

*Oncidium* orchid, also known as “dancing lady orchid”, is one of the most important pot and cut flowers in Taiwan, Southeast Asia and China. *Oncidium* belongs to the epiphytic orchids inherently known for slow growth rate. They require 2–3 years from the seedling stage to flowering [15]. Besides, the most commercialized cultivar *Onc.* ‘Gower Ramsey’ (*Onc.* GR) is highly susceptible to the soft-rot (*Erwinia carotovora*) disease [16]. Since most of the commercial *Oncidium* cultivars are self-incompatible [17], [18] or show high sterility which prevents breeding by cross-hybridization, it is necessary to improve new varieties to overcome these problems. Although gene transformation technics to improve the *Oncidium* cultivar property have been developed [19]–[21], this technology is highly dependent on the available information on the genetics of *Oncidium*. Recently, some progress has been made with regard to *Oncidium* flowering time [22]–[26] and pigment biosynthesis [19], [20], [27], [28], but little is known about growth regulation and disease resistance in orchids. We observed that colonization of *Oncidium* roots by *P. indica* results in growth promotion, similar to results obtained for Chinese cabbage and other plant species. *P. indica* is phylogentically related to fungi isolated from the rhizosphere of orchids, and helpful for the germination of the orchid seeds *in* *vitro* [29]. However, the underlying molecular mechanisms establishing and maintaining the symbiosis are barely studied. The identification of genes responding to the colonization may help to elucidate the molecular basis of orchid growth and development.

Recent evidence indicates that micro RNAs (miRNAs) play an important role in the interaction between plants and soil microbes [30]. MiRNAs are a class of endogenous small non-coding RNAs, often 20–22 nt long, which regulate gene expression by mediating gene silencing at transcriptional (TGS) and post-transcriptional (PTGS) levels, including histone modification, DNA methylation, RNA silicing and translational repression [31], [32]. They play important roles in numerous plant processes including development, differentiation and response to biotic and abiotic stresses [32]–[39]. Furthermore, miRNAs are rapidly upregulated in response to wounding and other stress stimuli [40]. The biogenesis and mode of action of miRNAs have been extensively reviewed [39], [41].

MiRNAs recognize their mRNA targets based on near-perfect complementarity and suppress expression of the target genes by guiding degradation or translational repression of the cognate mRNA targets [42]. Furthermore, they tend to be largely acting as an “early” regulator of signal transduction in operating at the transcription factor (TF) level in various systems [43], [44]. During symbiosis, miRNAs are involved in regulating plant nutritional balance [45], [46], hormone homeostasis and signaling [47] and symbiotic nodule spatial and temporal development [48], [49]. MiRNAs can respond quickly to infection by symbiotic bacteria. In soybean roots, a set of miRNAs were found to be intensively up- or down- regulated by infection with the rhizobial bacterium *Brodyrnia japonicum* where they target a wide range of mRNAs [47], [49].

Traditional cloning and studying of miRNAs rely on the cloning technology,which is labor-intense and low efficient. Due to the development of next generation sequence technology, genome-wide detection of miRNAs became much easier. Moreover, the read numbers of unigenes among different libraries helps to estimate the expression level of an individual small RNA [50]. Here, we used high-throughput technology to study the miRNAs in roots of the *Oncidium* orchid colonized or not colonized by *P. indica*. A great number of conserved and novel miRNAs were identified. The predicted target genes of these miRNAs were mainly cataloged to phytohormone signal perception and transduction, transcription factors, secondary metabolites and plant defence mechanisms. This study was focused on the identification and characterization of conserved and novel miRNAs. The target transcripts of some *Oncidium* miRNAs were identified. The data revealed that the identified miRNAs may participate in establishing an intricate network which regulates plant growth, root development and defense. This study may help to understand the molecular basis of the symbiotic interaction of *P. indica* with orchid plants.

## Results

### The Effect of *P. indica* on *Oncidium* GR Growth and Root Development

**Figure 1:**
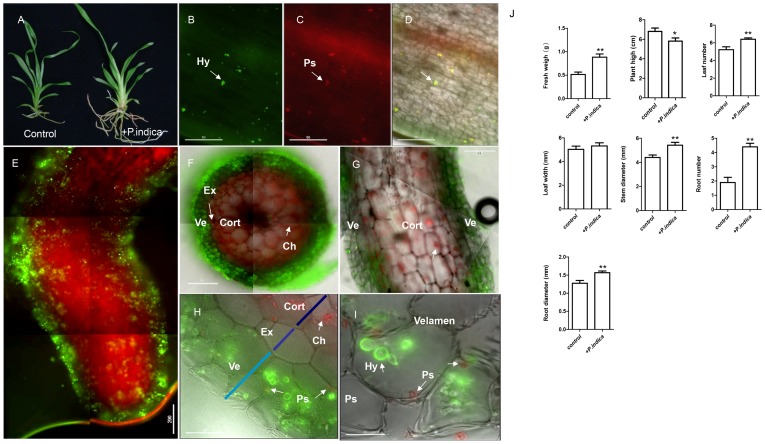
Growth effects of *P. indica* on *Oncidium* orchid. (A) Seedlings inoculated with *P. indica* for 8 weeks showed significantly enhanced growth and root development. (B–D) Anatomic structures of roots colonized by *P. indica* for 24 hours. Hyphae (green spots in B, arrow head) and penetration site (red spots in C, arrow head) were overlayed in bright field (D, arrow head). Bar = 50 µm. (E) Microscopic structure of roots colonized by *P. indica* for 5 days. A large number of hyphae were widespread over the root surface and tip, elongation zone and mature zone. Bar = 200 µm. (F, G) Microscopic structures of transverse sections and longitudinal sections of roots colonized by *P.indica* for 5 days. Hyphae fully colonized the velamen. Chloroplast autofluorescence (red) was also detected in cortex. Bar = 200 µm. (H) Microscopic structures of transverse section of roots colonized by *P. indica* for 5 days. *P. indica* was restricted in the velamen and not detectable in the exodermis of *Oncidium* roots. Chloroplast autofluorescence (red) was detected in cortex. Bar = 50 µm. (I) Micrograph of root cross sections from seedlings colonized with *P. indica* for 5 days. Without chlorophyll fluorescence in velamen, the penetration sites (red spot) were clearly detected. Bar = 20 µm. (J) Growth quantification of seedling colonized with *P. indica* for 8 weeks. Fresh weight, plant height, leaf number, leaf wide, stem diameter, root number and diameter were analyzed. Error bars represent SD for three independent experiments. *, *P* value<0.05; **, *P*<0.001. Hyphae were stained with chitin-specific WGA-AF488 (green). Penetrated sites (Ps) were stained with lectin-specific conA-AF633 (red) [88]. Samples were analyzed and photographed with an Olympus IX71 inverted microscope system (Japan). Ve, velamen; EX, exodermis; Cort: cortex; Ch: Chloroplast. Hy, hyphae; Ps, penetration site.

To understand the symbiotic interaction between *Oncidium* GR and *P. indica*, root tissue was analyzed after fungal colonization. *P. indica* penetrated into the root epidermal layers 24 h after inoculation (Figure 1B–D). Five days after inoculation, the hyphae were widely distributed over the root surface, and no significant difference was observed between root tip, elongation zone and differentiation zone (Figure 1E). This case is different from *A. thaliana* [6], [51] and barley [10], where hyphae preferentially colonized the differentiation zone. Cross and longitudinal sections show that hyphae fully colonized the velamen (Figure 1F, H). Since the velamen and exodermis do not contain plastids, the hyphae were easily detected after visualization by the carbohydrate binding lectin concanavalin A-AF633 (conA-633) (Figure 1H). Moreover, unlike other endophytic fungi which penetrate into the cortex cells through the exodermis [52], no hyphae were detected in the exodermis and cortex layer of *Oncidium*, even after a relative long period of co-cultivation (8 weeks). This suggests that *P. indica* may preferentially colonize dead cells in *Oncidium*, consistent with findings in barley where massive development of *P. indica* takes place in dead host cells [10].

Eight weeks after inoculation, the stems and roots developed better than orchids without *P. indica* co-cultivation. Furthermore, no necrotic lesions were observed (Figure 1A). With the colonization by *P. indica*, the fresh weight of the orchid seedlings is 2-fold higher than that of uninocculated control seedlings. Also the leaf number was more and the stem diameter was slightly thicker compared to the control. The number of the roots markedly increased approximately 2-fold and the diameter of the main root was 1∼2-fold bigger. These results indicated that the establishment of the beneficial symbiosis is relatively slow and mainly caused by an increase in the root biomass.

### Sequencing Small RNA from *Oncidium* Roots after Co-cultivation with *P. indica*

Roots colonized or not colonized by *P. indica* for 8 weeks were chosen for small RNAs sequencing. This time point was analyzed because a growth-promoting effect of the fungus is visible. After removal of the low quality contaminant and adapter reads, 24,570,250 and 24,744,141 clean read sequences were obtained from the libraries of control and colonized roots (Accession: SRP031471). The clean read sequences represented 97.15% and 99.66% of all reads, respectively. Their lengths range from 13 to 30 nucleotides (nt). Small RNAs of 20 to 24 nt represented 95.02% and 75.66%, respectively (Figure S1), of all small RNAs, indicating that both libraries are of high quality and can be used for further miRNA studies.

For both libraries, 17,036,953 unique sequences were obtained from a total of 49,314,391 clean reads. Among them, 62.56% were specific for the control roots, 27.47% were specific for the library of *P. indica*-colonized roots, and 9.97% overlapped in both libraries. After alignment to the Rfam 10.1 and Genbank databases, rRNA, snRNA and other no-coding RNA sequences were removed. The remaining 43,190 and 33,619 unique sequences in the two libraries were considered as potential miRNAs. However, most of the remaining unique sequences were still un-annotated. They accounted for 99.09% and 97.40% in the two libraries, respectively (Table 1). The high percentage of un-annotated small RNAs is mostly due to the insufficient genomic information of the *Oncidium* orchid.

**Table 1:** Category distribution of small RNAs in root tissues of *Oncidium* orchid ± *P. indica* colonization.

Category	Unique sRNAs	Percentage (%)	Unique sRNAs	Percentage (%)
Total	12,356,786	100%	6,378,130	100%
miRNA	43,190	0.35%	33,619	0.53%
rRNA	57,211	0.46%	101,266	1.59%
repeat	1	0.00%	1	0.00%
snRNA	1,973	0.02%	3,470	0.05%
snoRNA	870	0.01%	1,201	0.02%
tRNA	9,012	0.07%	26,409	0.41%
Un-annotation	12,244,529	99.09%	6,212,164	97.40%

−*P. indica*: roots without colonization by *P. indica*;

+*P. indica*: roots colonized by *P. indica.*

### Identification of Conserved miRNAs in Roots of *Oncidium* Orchid Colonized or Mock-treated by *P*. *indica*

For identification of conserved miRNAs, those with 20 to 24 nt were aligned with all known mature miRNAs from plants in the miRBase 19.0 Databank (E value = 1). Sequences with more than 4 nt differences were removed. Afterward, 2,789 from 17,036,953 unique sequences from both libraries showed homology to 86 miRNA families distributed in 41 species (Table S2). They are mainly found in *Cucumis melo, Malus domestica*, *Saccharum ssp*., and *Brachypodium distachyon* (Figure S2). Furthermore, it is well known, that miRNA* strands bound to Ago1 typically generate miRNAs starting with uridine, while Ago2-bound miRNA* usually starts with cytidine [53]. In our two libraries, most conserved miRNAs started with “U” (Figure S3), indicating that the miRNAs in *Oncidium* roots are mainly regulated by Ago1.

The vast majority and most abundantly conserved miRNAs were present in both libraries, and the number of miRNAs detectable only in one of the two libraries was low (Table S2). 1605 miRNAs belonging to 62 families were detected in both libraries. 1083 miRNAs belonging to 56 families were detected only in the library from *P. indica*-colonized roots. 101 miRNAs belonging to 31 families were detected only in the library from control roots. The miRNAs specifically detected in the library from *P. indica*-colonized roots were mir158a (t0035494; 41 reads), mir166l (t0039545; 36 reads) and mir528 (t0043631; 33 reads). The read number of those miRNAs specifically expressed in *P. indica*-colonized roots suggests that their accumulation is low (Table S2). Thus, miRNAs specifically expressed in the library of *P. indica*-colonized roots are difficult to detect and may not have significant impact on conserved miRNAs analysis. These results are similar to those reported from the plant pathogenic fungus *Sclerotinia sclerotiorum* [54], in which the number of low expressed conserved and specific miRNAs was also low. Furthermore, the number of conserved miRNAs in the control library was much lower than that in the library derived from *P. indica*–colonized roots. They were miRNA 397 (CK0328724; 6 reads), miRNA 171 (CK0347435; 6 reads) and miRNA 169 (CK0450655; 5 reads).

On the other hand, the number of reads of conserved miRNA families commonly occurring in both libraries showed significant differences between the two libraries. For example, the most abundant family mir528 generated 21287.27 TPM (times per million) in the *P. indica*-colonized library and only 3587.79 TPM in the control library. The top 20 miRNA families, including miR156 and miR528, were all significantly up-regulated in the *P. indica*-colonized library (Table 2). In contrast, a few miRNA families such as miR169, miR396, miR319, mir160, and mir5648 were down-regulated in the *P. indica*-colonized library, when compared to the control library (Table 2).

**Table 2:** Family members and counts of conserved miRNAs detected in root tissues of *Oncidium* orchid ± *P. indica* colonization.

		Count (TPM)				Count (TPM)	
Family	Member	−P.indica	+P.indica	Family	Member	−P.indica	+P.indica
miR156	83	2297.924	6689.814	miR479	1	0.0407	1.09135
miR158	294	2993.814	5677.85	miR528	7	0.3663	0.404204
miR159	213	1232.764	3019.887	miR529	4	0.2035	0.525465
miR160	257	1286.488	2528.901	miR535	9	0.2849	0.363783
miR162	200	687.0574	1778.496	miR829	2	0.2442	0.323363
miR164	83	351.2007	364.5513	miR845	5	0.2442	0.323363
miR166	117	160.928	370.2506	miR894	4	0.2442	0.161681
miR167	53	90.43549	270.8165	miR950	3	0.2442	0.161681
miR168	47	45.29915	128.2943	miR1310	299	3587.79	21287.27
miR169	39	55.59626	108.9329	miR1312	244	4714.53	20089.21
miR171	78	48.92145	96.07922	miR1314	378	6748.107	10194.83
miR172	36	13.10541	66.57235	miR2911	43	11.39601	47.29184
miR319	21	6.227106	10.26677	miR2916	14	11.02971	39.93533
miR390	11	2.767603	4.001617	miR2950	25	34.71713	12.08569
miR393	17	5.046805	1.333872	miR3699	27	2.035002	10.63056
miR394	34	1.953602	2.22312	miR3712	17	5.087505	5.578011
miR396	23	2.604803	1.172191	miR4995	3	0.0407	1.09135
miR397	1	0.691901	2.586904	miR5072	4	0.4884	0.485044
miR398	7	1.017501	1.172191	miR5083	12	0.2035	0.727567
miR399	4	0.976801	0.929669	miR5179	1	0.3663	0.444624
miR408	3	0	1.778496	miR5648	1	0.2035	0.404204

Count normalized by TPM;

−*P. indica*: roots without fungal colonization;

+*P. indica*: roots colonized with *P. indica.*

### Identification of Novel miRNAs in Roots of ± *P*. *indica*–colonized *Oncidium* Orchid

Since the whole genome sequence of *Oncidium* GR or any other orchid is not available, potential novel miRNAs were searched against the transcriptome database of *Oncidium* GR (http://predictor.nchu.edu.tw/oogb/) [26]. The secondary structure of miRNA precursors and dicer cleavage sites were predicted using the Mireap software. 46 putative miRNAs and 51 precursors were detected. Among them, 7 putative miRNAs, including *novel2*, *novel8, novel11, novel12, novel17*, *novel21* and *novel44* were detected in both libraries. Interestingly, 3 putative miRNAs (*novel9, novel10* and *novel25*) detected specifically in the *P. indica*-colonized library were identical in nucleotide sequence but different in their precursors. Similar results were found for *novel11*, *novel12* and *novel44* (Table S3).

These potential novel miRNAs were 20 to 23 nt long. Similar to conserved miRNAs, the majority (65.21%) of these novel miRNAs begins with “U” indicating that they were bound and processed by AGO1 [53]. The stem-loop structures of the potential precursors for the putative miRNAs were analyzed for RNA structure (Figure S4). The minimum free energy values ranged from 21.4 to 127.1 kcal/mol, and the average value was 49.99 kcal/mol.

The putative novel miRNAs showed much lower read numbers than those of the conserved miRNAs. 46 putative miRNAs generated only 175.09 TPM and 270.53 TPM in both libraries, respectively. The most abundantly detected putative miRNA was *novel11*, which was predicted to be generated from several different precursors and also named *novel12* and *novel44*. In total, they were expressed with 50.88 TPM in the control library, but were down-regulated with 3.71 TPM in the *P. indica*-colonized library. The predicted target gene was either CC-NBS-LRR (Unigene38669) or an rp1-like protein (JL929550.1) (Table S3), suggesting that they may play a role in triggering plant immunity [55], [56]. A second putative miRNA was *novel9*, which generated 49.43 TPM specifically in the *P. indica*-colonized library. It was also predicted to be generated from several different precursors, and also named *novel10* and *novel25*. The predicted target gene coding for a protein kinase (JL915652.1 or JL916309.1) may be involved in a wide range of symbiotic processes [57] (Table S3).

### Prediction of miRNA Target Genes

To understand the regulatory function of miRNAs during symbiosis, those miRNAs which were abundantly detected and significantly up-/down-regulated by *P*. *indica* were selected for further investigation. 1,834 conserved miRNAs belonging to 26 families were selected for target gene prediction. Due to the limited genome information, the information from the *Oncidium* mRNA database [26] was supplemented with mRNA database information from *Oryza sativa* and *A. thaliana*. After alignment in psRNAtarget, 702 best fit target candidates were obtained (Table S4).

**Figure 2:**
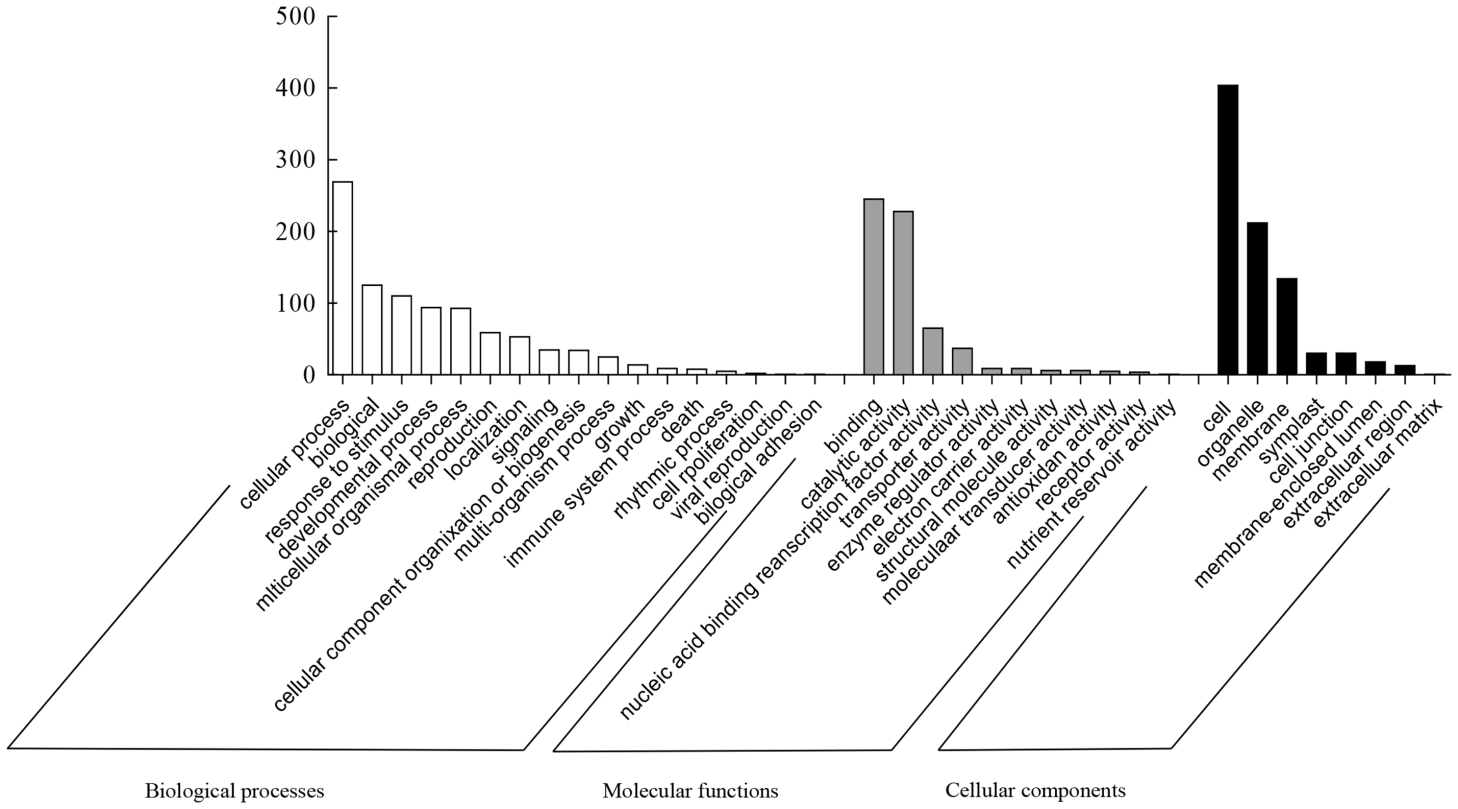
GO analysis of the conserved miRNA target candidates. The potential targets of miRNAs were predicted with psRNATarget by alignment to mRNAs from *Oncidium, Oryza stativa* and *A. thaliana*. The best hit results were chosen and annotated using Blast 2 GO.

Subsequently, annotation and GO analysis were conducted by Blast 2 GO. 653 targets were annotated and distributed in 38 categories (Table S4). In the subcategory of biological process, targets genes were mainly involved in root development, defense, hormone-mediated signaling, cell death and cell cycle (Figure 2). In the subcategory of molecular function, target genes were mainly related to amino acid metabolism, secondary metabolites, kinases, epigenetic and posttranscriptional modifications, redox regulation and transporters (Figure 2). In the subcategory of cellular components, target genes belonging the mitochondrial respiratory chain complex I, heterotrimeric G-protein complex, integral membrane proteins of the endoplasmic reticulum and chloroplast proteins were detected (Figure 2). Combined with the number of reads (Table S5), the most abundant miRNAs were predicted to target the genes involved in root development, such as mir535, mir156, mir166, mir168, mir393, mir894 (Table 3). Others are related to auxin signaling, such as mir164 and mir167 (Table 3). Another group of miRNAs, such as mir528, mir397, mir408, may target genes involved in cell wall metabolic and redox regulation (Table 3). Subsequently, we chose some representative miRNAs to identify the predicted targets by surveying the *Oncidium* GR transcriptomic database (Table S6). The results indicated that most of the predicted target genes of the conserved miRNAs were consistent with the results using the *O. sativa* and *A. thaliana* mRNA databases, but some miRNAs such as mir528, which was only detected in monocotyledons, were predicted to target different genes in each of the three databases. For example, the targets of mir528 were predicted as *FAR1* (NM_119978.3), *DUF3444* (NM_001084639.1) and *VTE1* (NM_119430.4) in the *A. thaliana* database; but they were predicted as plastocyanin-like protein (LOC_Os08g04310.1), a laccase precursor (LOC_Os01g44330.1), Cu/Zn-SOD protein (LOC_Os08g44770.1,2), multi-copper oxidase (LOC_Os01g03620.1) and L-ascorbate oxidase precursor (LOC_Os06g37150.1) [58]–[63] in the *O. sativa* database. However, α-lactate dehydrogenase (JL926279.1), pyruvate dehydrogenase e1 component subunit alpha (Unigene38362), F-box protein 13-like protein (Unigene13132) and a tetratricopeptide repeat-containing protein (Unigene39801) were predicted as target genes of mir528 in the *Oncidium* GR transcriptomic database. Similar results were found for mir390, which was predicted to target an LRR-kinase in *Oncidium* GR transcriptome database, but predicted as TSA3 in *A. thaliana* mRNA database.

**Table 3:** Target genes prediction for miRNAs in *P. indica* colonized- *Oncidium* roots.

Family	P/CK*	Sequence	Annotation	
mir156	0.66	TGACAGAAGAGAGTGAGCAC	promoter-binding protein spl	
mir159	0.48	TTTGGATTGAAGGGAGCTCTG	MYB transcription factors (glycosylation enzyme-like protein)	
mir160	1.34	TGCCTGGCTCCCTGTATGCCA	auxin response factors	
mir162	0.5	TCGATAAACCTCTGCATCCGGC	MicroRNA biogenesis, DCL1 (endoribonuclease dicer)	
mir164	0.43	TGGAGAAGCAGGGCACGTGCA	NAC domain transcription factors (an (no apical meristem)-like protein)	
mir166	0.53	TCGGACCAGGCTTCATTCCCC	HD-ZIP III transcription factors (class iii homeobox-leucine zipper protein)	
mir167	0.41	TGAAGCTGCCAGCATGATCTGA	auxin response factor 6, and 8	
mir168	0.51	TCGCTTGGTGCAGGTCGGGAC	argonaute1- partial	
mir169	1.91	CAGCCAAGGATGACTTGCCGA	nuclear transcription factor Y subunit a-5	
mir171	0.28	AGATATTGGAACGGTTCAATC	SCL-like transcription (promote shoot branching)	
mir172	0.73	CGAATCTTGATGATGCTGCAT	apetala2-like protein	
mir390	0.25	AAGCTCAGGAGGGATAGCGCC	LRR receptor-like kinase fls2-like	
mir393	0.68	TTCCAAAGGGATCGCATTGATC	TIR, transport inhibitor response 1	
mir394	0.11	TTGGCATTCTGTCCACCTCC	F-box family protein	
mir395	0.6	GTGAAGTGTTCGGATCGCTGC	potassium transporter 8-like	
mir396	2.79	TTCCACAGCTTTCTTGAACTG	growth-regulating factor 1	
mir397	0.34	TTGAGGGCAGCGTTGATGAAA	laccase 1a; β-6-tubulin	
mir398	1.02	TGTGTTCTCAGGTCGCCCCCG	CZSOD	
mir399	1.08	GCCGCAAGGAGATCTGCTCAC	transcription factor myb4	
mir408	0.98	TGCACTGCCTCTTCCCTGGCT	translation elongation factor ts	
mir528	0.18	TGGAAGGGGCATGCAGAGGAG	peroxidase-like protein	
mir529	0.37	AGAAGAGAGAGAGTACAGCCT	promoter-binding protein spl9	
mir535	0.23	TGACAACGAGAGAGAGCACG	senescence-associated protein 5	
mir829	3.94	AGGCTCTGATACCAAATGATGCAA	gag-pol polyprotein	
mir845	3.86	CGGCTCTGATACCAATTGTTGGGT	retrotransposon ty1-copia subclass	
mir894	0.38	GTTTCACGTCGGGTTCACCA	chloroplast rna binding protein	
mir2911	0.25	GCCGGCCGGGGGACGGACTGGGA	phospholipase c	
mir2916	0.52	GGGGCTCGAAGACGATCAGAT	calmodulin binding protein	
mir2950	0.33	TTCCATCTCTTGCACACTGGA	F-box only protein 13-like	
mir5293	3.7	GAAGAAGAAGTAGAAGAAGAAGAA	F-box protein	

P/CK: ratio of miRNA TPM between roots with/without colonization by *P.indica.*

In summary, the prediction of target genes revealed that the miRNAs in the *P*. *indica*–colonized roots of *Oncidium* may regulate a wide range of molecular processes. Especially, proteins involved in auxin signaling and development were identified (Table 3). Auxin has been shown to play a critical role in *P. indica*-mediated growth promotion and development in Chinese cabbage roots [14], [64]. This implicates that miRNAs play important roles in the regulation of growth promotion during symbiosis of *Oncidium* and *P. indica*.

### Expression Patterns of miRNAs and their Putative Targets in *P*. *indica* –colonized Roots

**Figure 3:**
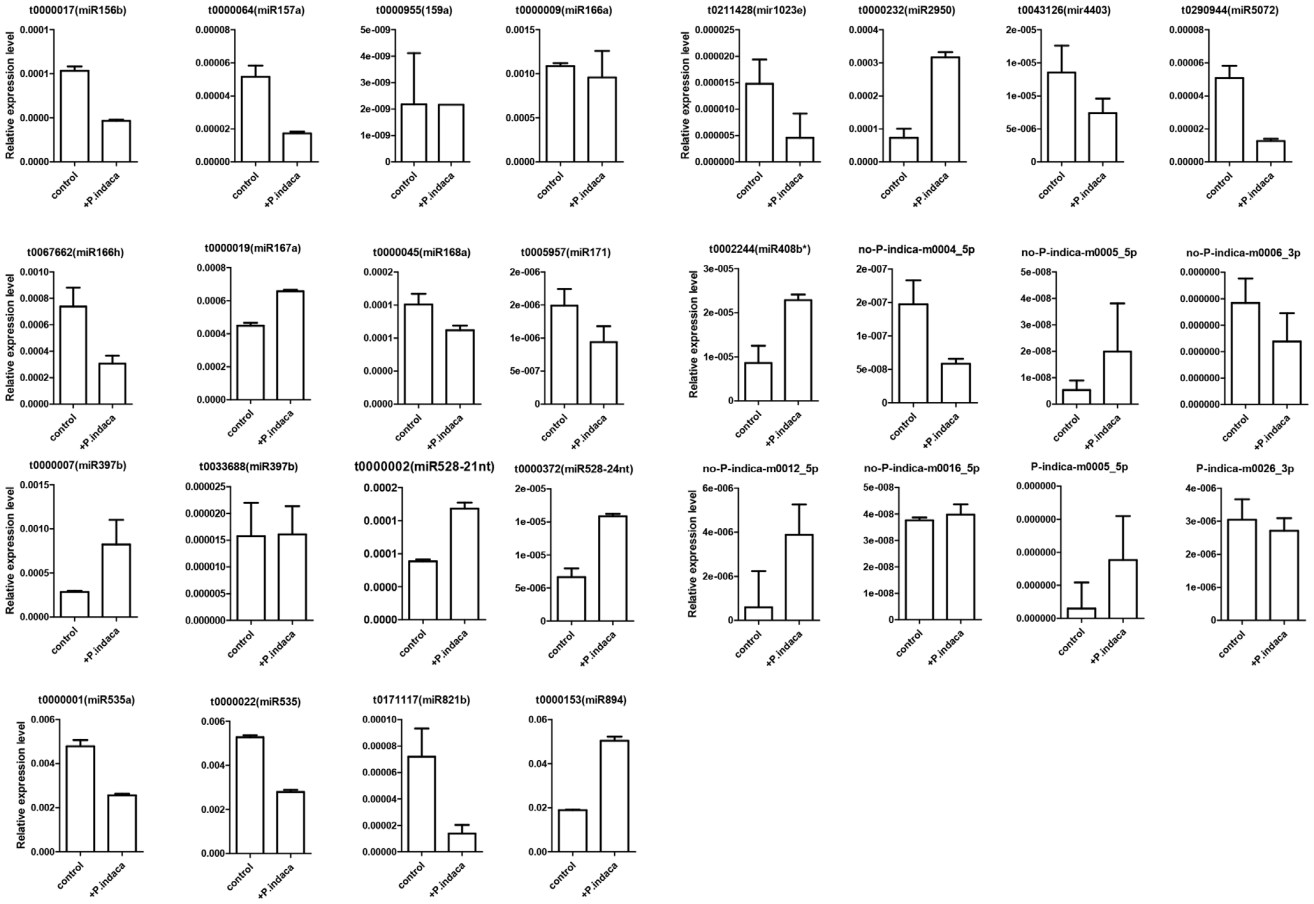
Expression analyses of miRNAs in *Oncidium* orchid ± *P. indica* by qPCR. Roots colonized ± *P. indica* for 8 weeks were sampled. Data represent the mean ± SD of 3 replicates and normalization by 5.8S rRNA. **e** represents the decimal point. * indicates antisense strand; no *P. ind*i*ca* indicates that the novel miRNAs were specifically found in the*–P. indica* orchid library. *P. indica* indicates that the novel miRNAs were specifically found in the +*P. indica* orchid library.

To elucidate the regulatory function of miRNAs on their putative targets, quantitative real time PCR (qPCR) was conducted to investigate the expression levels during different periods after colonization by *P. indica*. Most of the conserved miRNAs were detectable by qPCR (Figure 3). Members in the same family showed similar expression pattern in qPCR, such as mir156s, mir535s and mir528s. Furthermore, in accordance with bioinformatic data of the deep sequencing, the most abundant miRNAs, such as mir528 and mir156b did not show higher expression level than other miRNAs. Conversely, mir894, mir535a, mir166a, mir166h, mir167, mir168, mir397 and mir2950 showed higher expression levels in *P. indica* infected *Oncidium* root tissue (Figure 3). A few conserved miRNAs with low read numbers can barely be detected in the qPCR assay. These results indicated that qPCR data are largely in accordance with the read number data from deep sequencing.

**Figure 4:**
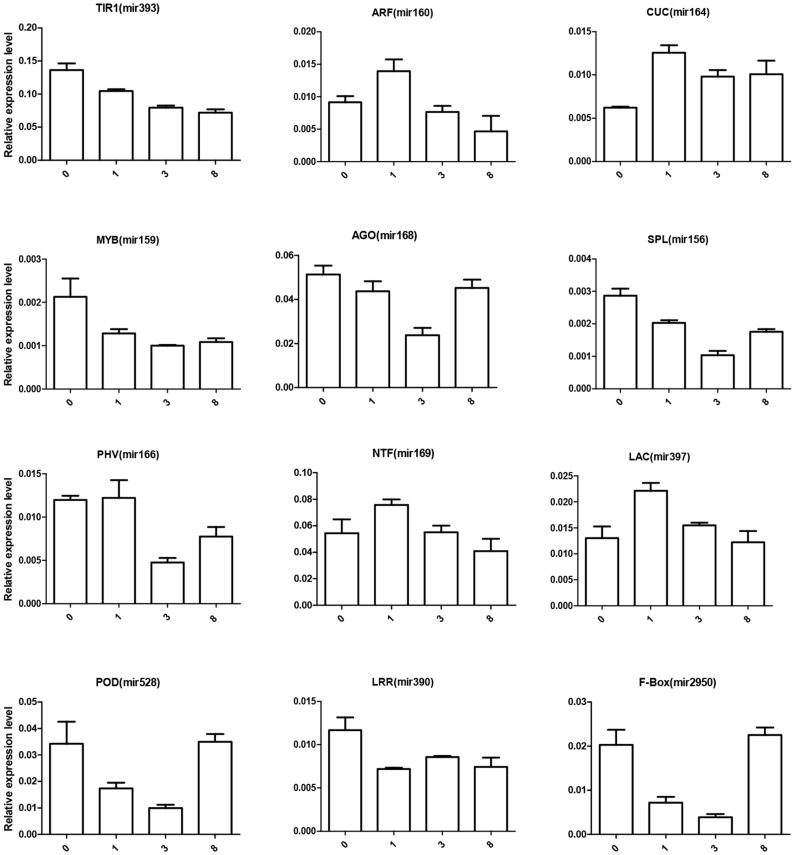
Expression analyses of the target genes of miRNAs by qPCR. Roots colonized with *P. indica* for 0, 1, 3 and 8 weeks were sampled and mRNA expression level was analyzed by qPCR. Data represents the mean ± SD of 3 replicates, and were normalized to the *Actin* mRNA level.

Subsequently, we used qPCR to study the expression pattern of miRNA target genes during the symbiotic process. Among the putative targets, mRNAs for auxin signal tranduction components showed significant changes (Figure 4). The mRNA for the auxin receptor protein TIR1, target of mir393, showed a gradual down-regulation from 0 to 8 weeks. The mRNAs for auxin response factors (ARFs), targets of mir160, were up-regulated during the first week followed by down-regulation between the 3^rd^ and 8^th^ week. We could not detect the two *ARFs*, type 6 and 8, targets of mir167, in our qPCR assay, suggesting that the transcript abundance is low. ARFs are considered to be a key player in regulating the auxin signal pathway postively or negatively, and they are involved in auxin homeostasis by conjugating free auxin via GH3 [65].

Plants need a “reprogram cycle” to adjust growth and development during a symbiotic process [66]. The mRNA level involved in development, such as for the squamosa promoter binding protein, SPL, AGO1, and for the class III homeobox-leucine zipper transcription factor (PHV; HD-ZIP III transcription factors) were regulated by mir156/mir529, mir168 and mir166, respectively [67]. Their expression was down-regulated from the 1^st^ to the 3^rd^ week followed by up-regulation at the 8^th^ week (Figure 4). Apparently, these mRNAs are first repressed and later-on stimulated. Another mRNA encoding the *Nuclear Factor Y* (*NTFY*) regulated by mir169, which is critical for symbiotic nodule development [48], was up-regulated during early colonizing periods and declined later (Figure 4).

Furthermore, cell wall metabolism-related genes, such as *laccase* (*LAC*) which is targeted by mir397, were up-regulated during the 1^st^ week, before they returned to the normal level between the 3^rd^ and 8^th^ week (Figure 4). Several *LRR-kinase* genes (*LRR*), which are targets of mir390, were down-regulated after colonization. *F-box* genes, targets of mir2950, were also down-regulated between the 1^st^ and 3^rd^ week followed by normal levels at the 8^th^ week (Figure 4). LRR-kinases [68] and F-box proteins [69] derive from large gene families and the proteins are involved in many different biological processes, thus the exact function of these genes during symbiosis needs to be further studied.

Considering that these targets were significantly up- or down-regulated and the miRNAs were abundantly detected in the high-throughput sequencing, it is interesting to study the interaction between the miRNAs and their targets. Full understanding of the physiological function of these miRNAs and targets during symbiosis may help to unravel their roles in the symbiosis.

## Discussion

### Deep Sequencing miRNA in Roots of *Oncidium*

*P. indica* colonizes the roots of a wide host range, thereby promoting growth and plant performance [7]. This is often associated with alterations in root architecture [13]. In this study, we demonstrate that the fresh weight of *Oncidium* seedlings was significantly increased after 8 weeks of co-cultivation with the fungus, which includes a higher leaf number, bigger stem diameter, an increase in the root number and root diameter and longer height of the seedlings (Figure 1). Due to the inherently slow growth of orchids, growth promoting effects have practical implications for commercial purposes. *P. indica* was reported to be isolated from the rhizosphere of the shrubs *Zizyphus nummularia* and *Prosopis juliflora* in the Indian desert [3]. It is closely related to the multinuclear fungi *Rhizoctonia* and *Sebacinales,* which are naturally associated with diverse orchid plants, and are capable in promoting orchid seed germination [29]. Here, we provide evidence that *P. indica* promotes growth of both aerial part and roots in orchids.

The purpose of this work is to understand the functions and regulatory mechanisms of miRNAs in *Oncidium* orchid growth regulation during the symbiosis with *P. indica*. A high-throughput sequencing and comparative expression analysis were conducted. In total, 17,036,953 unique sequences from 24,570,250 and 24,744,141 clean reads in control and *P. indica*-colonized root libraries were obtained, and 13,736 unique sequences showed homology to 941 miRNA families distributed in 45 plant species. Above all, there are 46 putative novel miRNAs and 51 corresponding precursors.

Orchids are ancient species which emerged shortly after the mass extinction. They became the most diverse plant family over the world [70]. To date, only a few studies on miRNAs in orchid were performed. They demonstrated that only a very few miRNAs are conserved between species. The majority of known miRNAs are family- or species-specific miRNAs, which derived from reversed duplication events or accumulation of mutations within inverted repeats. The differential expression of miRNAs may be essential for the physiological function and contributes to species evolution [71], [72]. To date, miRNA studies have been carried out on the tropic orchid *Phalaenopsis* [73]. Compared to *Oncidium*, the most abundant top 20 miRNAs conserved in roots, such as mir528, mir156, mir166, mir167, mir168, mir159, mir529, and mir894, were also abundantly found in *Phalaenopsis*. The *Oncidium*-specific miRNAs are mir397, mir408, mir2916, mir2950, mir5077, and mir5059, while *Phalaenopsis*–specific miRNAs are mir169, mir172, mir396, mir1318 and mir2911. Furthermore, we also detected a considerable number of new members of conserved miRNA families and novel miRNAs. It is known that the variation in miRNA precursors foldback, processing efficiency and miRNA size may influence the maturation type of miRNAs. The young miRNAs are often expressed in very low levels and in restricted spatial/temporal patterns, and some of the newly identified miRNAs have no obvious function [72]. Most of the newly identified miRNAs in this study had low number of reads and were hardly detectable by qPCR. Further research is required for eludicating their function in *Oncidium*.

### Role of miRNAs and their Targets on Regulating Phytohormone Levels and Root Development during Symbiosis

The miRNAs involved in auxin signalling functions were easily detectable in our experiment, including miR160, mir164, mir167, mir393, miR394 and mir5293. Besides, mir390, another auxin-related miRNA is related to the *Arabidopsis TAS*3 RNAs, and predicted to target the mRNA encoding a LRR-kinase, which is responsible for regulating lateral root growth [74]. This miRNA is common in monocotyledonous plants [75]. Phytohormones play key roles in plant growth and development, and are also required for resistance against abiotic and biotic stresses [11], [12], [76], [77]. Previously, phytohormone metabolism genes including auxin, gibberellin (GA) and abscisic acid (ABA) were found to be up-regulated after colonization with *P. indica* in barley roots [11], and auxin was considered to be a key phytohormone in plant growth promotion triggered by *P. indica* [14], [64]. In our experiments, auxin-related genes such as *TIR1*, *ARF* and *CUC*, which were regulated by mir393, mir160 and mir164, were up- or down-regulated during different phases of the symbiosis, and a putative GA pathway gene, *MYB,* regulated by mir159, was down-regulated during symbiosis. Auxin and GA probably function as a SA suppressor or JA enhancer in symbiotic processes [11], [78]–[80]. Taken together, the high expression level of this group of miRNAs may probably balance growth and defense during symbiosis.

Another group of miRNA including mir156, mir166 and mir169, which targets mRNAs for the transcription factor genes *SPL*, *PHV/PHB* and *NTF*, were also abundantly detected in our experiment (Table S5). Also mir162 and mir168 are highly expressed. They function as feed-back regulators to miRNAs and catalyse the *AGO1* and *DCL* mRNAs (Table S5). AGO1 and the transcription factor PHV/B may cooperatively control the development of shoot apical meristem (SAM) and the root apical meristem (RAM) [81]. Plants which fail to express these miRNA-targets have lesions in root development [66], [81]–[87]. Furthermore, these miRNAs also accumulated in the nodules of *Medicago truncatula* after colonization by the endophytic fungus [48], [49]. It appears that plants reprogram the development of the SAM and RAM in the symbiotic interaction with microbes by regulating the expression of these miRNA in critical spatial and temporal patterns [48].

MiRNAs such as mir397 or mir408 may be necessary for *Oncidium* to enhance anti-oxidative capacity and resistance against stress by mediating copper distribution and playing roles in the cell wall metabolism during growth and development processes trigged by *P. indica*.

### Conclusions

We report on the miRNA profiling of *Oncidium* orchid after colonization by *P. indica*. The miRNAs and their target genes illustrate that the physiological metabolism of *Oncidium* is reprogrammed in response to the symbiotic interaction. Genes participating in phytohormone signaling, for cell wall metabolism and regulatory transcription factors are major targets of the *P. indica*-induced miRNAs in *Oncidium* roots. Therefore, we propose that *P. indica* alters the miRNA pattern to establish an intricate network for growth promotion, developmental reprogramming and enhances resistance in the roots of *Oncidium*. Several novel unique miRNAs were detected, for which a function could not yet be identified. Further investigations on the molecular mechanism of miRNAs in symbiotic interactions are of huge significance.

## Materials and Methods

### Plant Materials

*Oncidium* GR flask seedlings were cultured on 1/2 Murashige-Skoog (MS) medium, and incubated with 16 h photoperiod (100 µmol m^−2^ s^−1^) at 25°C. Seedlings about 3 cm high were selected and transferred to fresh medium. After 10 days of acclimating cultivation, plants were inoculated with *P. indica* mycelium as described [52]. Briefly, one agar block of 5 mm in diameter was placed per seedling at a distance of 1 cm from the adventitious root. The seedlings were either exposed to an agar block containing *P. indica* (+ *P. indica*) or to a block without the fungus (control; - *P. indica*). After 8 week of co-cultivation, the biomass of the seedlings was determined. They were then immediately frozen in liquid N_2_ and stored at −80°C for RNA extraction and other assays.

### Anatomic Dissection of Root Tissues Colonized by P. indica

Root samples were fixed in 4% formaldehyde in 20 mM PBS, pH 7.4, for 60 minutes at 37°C followed by dehydration with 5% sucrose in 20 mM PBS, pH 7.4 at 4°C overnight. Thin sections were cut by free hand and stained with chitin-specific WGA, Alexa Fluor^R^ 488 conjugate (WGA-AF488, Invitrogen USA) or Alexa Fluor^R^ conjugated to succinylated concanavalin A (ConA-AF633, Invitrogen USA), according to the manufactureŕs protocol. Sections were photographed by Olympus IX17, WGA-AF488 was excited at 488 nm and detected at 505–540 nm, ConA-AF633 was excited at 546 and detected at 600–660 nm.

### Small RNA Library Construction and Sequencing

Total RNA was isolated from control roots and those colonized with *P. indica* using the pine tree method. The RNA integrity number (RIN) was examined on Agilent 2100; when the RIN was >8.0, the samples were sent to BGI (Shengzheng China) for small RNA *Solexa* sequencing. In brief: the small molecule RNAs were separated by 15% (w/v) PAGE (18–30 nt), subsequently, the purified small RNAs were ligated to a pair of *Solexa* adaptors to the 5′ and 3′ ends, reverse transcribed to cDNA using a RT primer, and finally amplified by PCR and sequenced. The results were deposited in Sequence Read Archive (SRA) on NCBI website. The accession number is SRP031471.

### Identification of Conserved and Novel miRNAs

After removal of the adapter sequences, low quality tags and contaminated sequences, rRNA, tRNA, snRNA, etc. were identified by alignment to the Rfam 10.1 and Genbank databases. Conserved miRNA were identified by search against known plant miRNAs in miRBase 19.0 (http://www.mirbase.org). Novel miRNAs were predicted by Mireap (http://sourceforge.net/projects/mireap/). Secondary structure of all candidate precursors was mapped in RNAstructure Web (http://rna.urmc.rochester.edu/RNAstructureWeb/).

### Prediction and Annotation of Potential Targets of miRNAs

The potential targets of miRNAs were predicted with psRNATarget (plantgrn.noble.org/psRNATarget/) by alignment to mRNAs from *Oncidium*, *Oryza stativa* and *A. thaliana* (data were downloaded from the NBCI GenBank), the best hit results were chosen and annotated using Blast 2 GO.

### Expression Analysis of miRNAs and their Potential Targets by Quantitative Real Time PCR

For expression analysis of miRNAs and their potential targets, RNA samples were isolated from roots, which were collected from 3 independent replicates of seedlings cultured under the same condition as described above. Small and high molecular weight RNAs were isolated by LiCl and isopropanol methods, respectively [73]. For miRNA quantitative real time PCR (qPCR), the cDNAs were synthesized with the QuantiMir RT kit (RA420AU-3; SBI). Abundantly expressed miRNAs (based on the number of reads) and novel miRNAs were chosen for analysis (Figure S2). Using 5.8S rRNA as internal control, expression analyses of these miRNAs were performed with the Applied Biosystems 7500 Fast Real-Time PCR System using the SYBR Green PCR master kit (with ROX). The qPCR reaction was performed in a 30 µl reaction system and programmed as follow: 95°C for 2 min; 40 cycles of 95°C 15 s, 65°C 15 s, 72°C 30 s, following by a dissociation program from 60°C to 95°C. Each reaction was repeated three times.

For potential target qPCR, the cDNAs were synthesized by Revert Aid First Strand cDNA Synthesis kit (#k1622; Fermentas). Using actin as internal control, the qPCR reaction was carried out in a 20 µl reaction system, and the qPCR program is identical to that described above, but annealing was performed at 60°C. Primers information is shown in Table S1.

## Supporting Information

Table S1 **Primer sequance information.**
(XLSX)

Table S2 **Conserved miRNA sequence information.**
(XLSX)

Table S3 **The novel miRNA sequence information.**
(XLSX)

Table S4 **selected sRNAs_GO term from target of miRBase.**
(XLSX)

Table S5 **abundant sRNAs_GO term from target of miRBase.**
(XLSX)

Table S6 **Confirmed target pretiction and annotation in Oncidium.**
(XLSX)

Figure S1 **Length distribution of small RNA.**
(PPTX)

Figure S2 **Distribution of plant species conserved miRNA homology.**
(PPTX)

Figure S3 **Frequency of the first nucleotide of the conserved miRNA.**
(PPTX)

Figure S4 **Secondary structure of miRNA precursor.**
(PPTX)

## References

[1] Sherameti I, Shahollari B, Venus Y, Altschmied L, Varma A, et al (2005) The endophytic fungus *Piriformospora indica* stimulates the expression of nitrate reductase and the starch-degrading enzyme glucan-water dikinase in tobacco and *Arabidopsis* roots through a homeodomain transcription factor that binds to a conserved motif in their promoters. J Biol Chem 280: 26241–26247. 15710607 10.1074/jbc.M500447200

[2] Yadav V, Kumar M, Deep DK, Kumar H, Sharma R, et al (2010) A phosphate transporter from the root endophytic fungus *Piriformospora indica* plays a role in phosphate transport to the host plant. J Biol Chem 285: 26532–26544. 20479005 10.1074/jbc.M110.111021 PMC2924090

[3] Varma A, Savita V, Sudha, Sahay N, Butehorn B, et al (1999) *Piriformospora indica*, a cultivable plant-growth-promoting root endophyte. Appl Environ Microbiol 65: 2741–2744. 10347070 10.1128/aem.65.6.2741-2744.1999 PMC91405

[4] Waller F, Achatz B, Baltruschat H, Fodor J, Becker K, et al (2005) The endophytic fungus *Piriformospora indica* reprograms barley to salt-stress tolerance, disease resistance, and higher yield. Proc Natl Acad Sci U S A 102: 13386–13391. 16174735 10.1073/pnas.0504423102 PMC1224632

[5] Baltruschat H, Fodor J, Harrach BD, Niemczyk E, Barna B, et al (2008) Salt tolerance of barley induced by the root endophyte *Piriformospora indica* is associated with a strong increase in antioxidants. New Phytol 180: 501–510. 18681935 10.1111/j.1469-8137.2008.02583.x

[6] Stein E, Molitor A, Kogel KH, Waller F (2008) Systemic resistance in *Arabidopsis* conferred by the mycorrhizal fungus *Piriformospora indica* requires jasmonic acid signaling and the cytoplasmic function of NPR1. Plant Cell Physiol 49: 1747–1751. 18842596 10.1093/pcp/pcn147

[7] Sirrenberg A, Gobel C, Grond S, Czempinski N, Ratzinger A, et al (2007) *Piriformospora indica* affects plant growth by auxin production. Physiol Plant 131: 581–589. 18251849 10.1111/j.1399-3054.2007.00983.x

[8] Qiang X, Weiss M, Kogel KH, Schäfer P (2012) *Piriformospora indica*-a mutualistic basidiomycete with an exceptionally large plant host range. Mol Plant Pathol 13: 508–518. 22111580 10.1111/j.1364-3703.2011.00764.x PMC6638644

[9] Qiang X, Zechmann B, Reitz MU, Kogel KH, Schäfer P (2012) The mutualistic fungus *Piriformospora indica* colonizes *Arabidopsis* roots by inducing an endoplasmic reticulum stress-triggered caspase-dependent cell death. Plant Cell 24: 794–809. 22337916 10.1105/tpc.111.093260 PMC3315247

[10] Deshmukh S, Huckelhoven R, Schäfer P, Imani J, Sharma M, et al (2006) The root endophytic fungus *Piriformospora indica* requires host cell death for proliferation during mutualistic symbiosis with barley. Proc Natl Acad Sci U S A 103: 18450–18457. 17116870 10.1073/pnas.0605697103 PMC1697795

[11] Schäfer P, Pfiffi S, Voll LM, Zajic D, Chandler PM, et al (2009) Manipulation of plant innate immunity and gibberellin as factor of compatibility in the mutualistic association of barley roots with *Piriformospora indica* . Plant J 59: 461–474. 19392709 10.1111/j.1365-313X.2009.03887.x

[12] Camehl I, Sherameti I, Venus Y, Bethke G, Varma A, et al (2010) Ethylene signalling and ethylene-targeted transcription factors are required to balance beneficial and nonbeneficial traits in the symbiosis between the endophytic fungus *Piriformospora indica* and *Arabidopsis thaliana* . New Phytol 185: 1062–1073. 20085621 10.1111/j.1469-8137.2009.03149.x

[13] Peskan-Berghofer T, Shahollari B, Giong PH, Hehl S, Markert C, et al (2004) Association of *Piriformospora indica* with *Arabidopsis* thaliana roots represents a novel system to study beneficial plant-microbe interactions and involves early plant protein modifications in the endoplasmic reticulum and at the plasma membrane. Physiol Plant 122: 465–477.

[14] Lee YC, Johnson JM, Chien CT, Sun C, Cai D, et al (2011) Growth promotion of Chinese cabbage and *Arabidopsis* by *Piriformospora indica* is not stimulated by mycelium-synthesized auxin. Mol Plant Microbe Interact 24: 421–431. 21375386 10.1094/MPMI-05-10-0110

[15] Chugh S, Guha S, Rao IU (2009) Micropropagation of orchids: A review on the potential of different explants. Scientia Horticulturae 122: 507–520.

[16] You SJ, Liau CH, Huang HE, Feng TY, Prasad V, et al (2003) Sweet pepper ferredoxin-like protein (*pflp*) gene as a novel selection marker for orchid transformation. Planta 217: 60–65. 12721849 10.1007/s00425-002-0970-7

[17] Alcantara S, Semir J, Solferini VN (2006) Low genetic structure in an epiphytic Orchidaceae (*Oncidium hookeri*) in the Atlantic rainforest of South-eastern Brazil. Ann Bot 98: 1207–1213. 17008349 10.1093/aob/mcl202 PMC2803584

[18] Singer RB KS (2003) Notes on the pollination biology of *Notylia nemorosa* (Orchidaceae: Oncidiinae): do pollinators necessarily promote cross-pollination? J Plant Res 116: 19–25. 12605296 10.1007/s10265-002-0064-4

[19] Chiou CY, Pan HA, Chuang YN, Yeh KW (2010) Differential expression of carotenoid-related genes determines diversified carotenoid coloration in floral tissues of *Oncidium* cultivars. Planta 232: 937–948. 20635095 10.1007/s00425-010-1222-x

[20] Chiou CY, Yeh KW (2008) Differential expression of *MYB* gene (*OgMYB1*) determines color patterning in floral tissue of *Oncidium* Gower Ramsey. Plant Mol Biol 66: 379–388. 18161007 10.1007/s11103-007-9275-3

[21] Liau CH, Lu JC, Prasad V, Hsiao HH, You SJ, et al (2003) The sweet pepper ferredoxin-like protein (*pflp*) conferred resistance against soft rot disease in *Oncidium* orchid. Transgenic Res 12: 329–336. 12779121 10.1023/a:1023343620729

[22] Tan J, Wang HL, Yeh KW (2005) Analysis of organ-specific expressed genes in *Oncidium* orchid by subtractive expressed sequence tags library. Biotechnol Lett 27: 1517–1528. 16231226 10.1007/s10529-005-1468-8

[23] Wang CY, Chiou CY, Wang HL, Krishnamurthy R, Venkatagiri S, et al (2008) Carbohydrate mobilization and gene regulatory profile in the pseudobulb of *Oncidium* orchid during the flowering process. Planta 227: 1063–1077. 18188590 10.1007/s00425-007-0681-1

[24] Chang YY, Chiu YF, Wu JW, Yang CH (2009) Four orchid (*Oncidium* Gower Ramsey) *AP1*/*AGL9*-like MADS box genes show novel expression patterns and cause different effects on floral transition and formation in *Arabidopsis thaliana* . Plant Cell Physiol 50: 1425–1438. 19541596 10.1093/pcp/pcp087

[25] Hou CJ, Yang CH (2009) Functional analysis of *FT* and *TFL1* orthologs from orchid (*Oncidium* Gower Ramsey) that regulate the vegetative to reproductive transition. Plant Cell Physiol 50: 1544–1557. 19570813 10.1093/pcp/pcp099

[26] Chang YY, Chu YW, Chen CW, Leu WM, Hsu HF, et al (2011) Characterization of *Oncidium* ‘Gower Ramsey’ transcriptomes using 454 GS-FLX pyrosequencing and their application to the identification of genes associated with flowering time. Plant Cell Physiol 52: 1532–1545. 21785129 10.1093/pcp/pcr101

[27] Liu XJ, Chuang YN, Chiou CY, Chin DC, Shen FQ, et al (2012) Methylation effect on chalcone synthase gene expression determines anthocyanin pigmentation in floral tissues of two *Oncidium* orchid cultivars. Planta 236: 401–409. 22391855 10.1007/s00425-012-1616-z

[28] Chiou CY, Wu K, Yeh KW (2008) Characterization and promoter activity of chromoplast specific carotenoid associated gene (*CHRC*) from *Oncidium* Gower Ramsey. Biotechnol Lett 30: 1861–1866. 18575811 10.1007/s10529-008-9767-5

[29] Schäfer P, Kogel K-H (2009) The sebacinoid fungus *Piriformospora indica*: an Orchid mycorrhiza which may increase host plant reproduction and fitness. Mycota 5: 99–112.

[30] Simon SA, Meyers BC, Sherrier DJ (2009) MicroRNAs in the rhizobia legume symbiosis. Plant Physiol 151: 1002–1008. 19789286 10.1104/pp.109.144345 PMC2773061

[31] Baulcombe D (2004) RNA silencing in plants. Nature 431: 356–363. 15372043 10.1038/nature02874

[32] Hajdarpasic A, Ruggenthaler P (2012) Analysis of miRNA expression under stress in *Arabidopsis thaliana* . Bosn J Basic Med Sci 12: 169–176. 22938544 10.17305/bjbms.2012.2471 PMC4362426

[33] Chen X (2012) Small RNAs in development - insights from plants. Curr Opin Genet Dev 22: 361–367. 22578318 10.1016/j.gde.2012.04.004 PMC3419802

[34] Khan G, Declerck M, Sorin C, Hartmann C, Crespi M, et al (2011) MicroRNAs as regulators of root development and architecture. Plant Mol Biol 77: 1573–5028. 10.1007/s11103-011-9793-x 21607657

[35] Luo M, Zhang ZM, Gao J, Zeng X, Pan GT (2011) The role of miR319 in plant development regulation. Yi Chuan 33: 1203–1211. 22120075 10.3724/sp.j.1005.2011.01203

[36] Sunkar R, Li YF, Jagadeeswaran G (2012) Functions of microRNAs in plant stress responses. Trends Plant Sci 17: 196–203. 22365280 10.1016/j.tplants.2012.01.010

[37] Phillips JR, Dalmay T, Bartels D (2007) The role of small RNAs in abiotic stress. FEBS Lett 581: 3592–3597. 17451688 10.1016/j.febslet.2007.04.007

[38] Khraiwesh B, Zhu JK, Zhu J (2012) Role of miRNAs and siRNAs in biotic and abiotic stress responses of plants. Biochim Biophys Acta 1819: 137–148. 21605713 10.1016/j.bbagrm.2011.05.001 PMC3175014

[39] Guleria P, Mahajan M, Bhardwaj J, Yadav SK (2011) Plant small RNAs: biogenesis, mode of action and their roles in abiotic stresses. Genomics, Proteomics & Bioinformatics 9: 183–199. 10.1016/S1672-0229(11)60022-3 PMC5054152 22289475

[40] Tang S, Wang Y, Li Z, Gui Y, Xiao B, et al (2012) Identification of wounding and topping responsive small RNAs in tobacco (*Nicotiana tabacum*). BMC Plant Biol 12: 28. 22353177 10.1186/1471-2229-12-28 PMC3306195

[41] Bartel DP (2004) MicroRNAs: genomics, biogenesis, mechanism, and function. Cell 116: 281–297. 14744438 10.1016/s0092-8674(04)00045-5

[42] Li X, Zhang YZ (2005) Computational detection of microRNAs targeting transcription factor genes in *Arabidopsis thaliana* . Comput Biol Chem 29: 360–367. 16221572 10.1016/j.compbiolchem.2005.08.005

[43] Szittya G, Moxon S, Santos DM, Jing R, Fevereiro MP, et al (2008) High-throughput sequencing of *Medicago truncatula* short RNAs identifies eight new miRNA families. BMC Genomics 9: 593. 19068109 10.1186/1471-2164-9-593 PMC2621214

[44] Voinnet O (2009) Origin, biogenesis, and activity of plant microRNAs. Cell 136: 669–687. 19239888 10.1016/j.cell.2009.01.046

[45] Branscheid A, Sieh D, Pant BD, May P, Devers EA, et al (2010) Expression pattern suggests a role of MiR399 in the regulation of the cellular response to local Pi increase during arbuscular mycorrhizal symbiosis. Mol Plant Microbe Interact 23: 915–926. 20521954 10.1094/MPMI-23-7-0915

[46] Devers EA, Branscheid A, May P, Krajinski F (2011) Stars and symbiosis: microRNA- and microRNA*-mediated transcript cleavage involved in arbuscular mycorrhizal symbiosis. Plant Physiol 156: 1990–2010. 21571671 10.1104/pp.111.172627 PMC3149951

[47] Subramanian S, Fu Y, Sunkar R, Barbazuk WB, Zhu JK, et al (2008) Novel and nodulation-regulated microRNAs in soybean roots. BMC Genomics 9: 160. 18402695 10.1186/1471-2164-9-160 PMC2335117

[48] Combier JP, Frugier F, de Billy F, Boualem A, El-Yahyaoui F, et al (2006) *MtHAP2–1* is a key transcriptional regulator of symbiotic nodule development regulated by microRNA169 in *Medicago truncatula* . Genes Dev 20: 3084–3088. 17114582 10.1101/gad.402806 PMC1635144

[49] Lelandais-Briere C, Naya L, Sallet E, Calenge F, Frugier F, et al (2009) Genome-wide *Medicago truncatula* small RNA analysis revealed novel microRNAs and isoforms differentially regulated in roots and nodules. Plant Cell 21: 2780–2796. 19767456 10.1105/tpc.109.068130 PMC2768930

[50] Dillies M-A, Rau A, Aubert J, Hennequet-Antier C, Jeanmougin M, et al (2013) A comprehensive evaluation of normalization methods for Illumina high-throughput RNA sequencing data analysis. Brief Bioinform 14: 671–683. 22988256 10.1093/bib/bbs046

[51] Jacobs S, Zechmann B, Molitor A, Trujillo M, Petutschnig E, et al (2011) Broad-spectrum suppression of innate immunity is required for colonization of *Arabidopsis* roots by the fungus *Piriformospora indica* . Plant Physiol 156: 726–740. 21474434 10.1104/pp.111.176446 PMC3177271

[52] Senthilkumar S, Krishnamurthy KV, Britto SJ, Arockiasamy DI (2000) Visualization of orchid mycorrhizal fungal structures with fluorescence dye using epifluorescence microscopy. Current Science 79: 1527–1528.

[53] Ghildiyal M, Xu J, Seitz H, Weng Z, Zamore PD (2010) Sorting of Drosophila small silencing RNAs partitions microRNA* strands into the RNA interference pathway. RNA 16: 43–56. 19917635 10.1261/rna.1972910 PMC2802036

[54] Zhou J, Fu Y, Xie J, Li B, Jiang D, et al (2012) Identification of microRNA-like RNAs in a plant pathogenic fungus *Sclerotinia sclerotiorum* by high-throughput sequencing. Mol Genet Genomics 287: 275–282. 22314800 10.1007/s00438-012-0678-8

[55] Eitas TK, Dangl JL (2010) NB-LRR proteins: pairs, pieces, perception, partners, and pathways. Curr Opin Plant Biol 13: 472–477. 20483655 10.1016/j.pbi.2010.04.007 PMC2910844

[56] Ramakrishna W, Emberton J, Ogden M, SanMiguel P, Bennetzen JL (2002) Structural analysis of the maize *Rp1* complex reveals numerous sites and unexpected mechanisms of local rearrangement. Plant Cell 14: 3213–3223. 12468738 10.1105/tpc.006338 PMC151213

[57] Stone JM, Walker JC (1995) Plant protein kinase families and signal transduction. Plant Physiol 108: 451–457. 7610156 10.1104/pp.108.2.451 PMC157363

[58] Abdel-Ghany SE, Pilon M (2008) MicroRNA-mediated systemic down-regulation of copper protein expression in response to low copper availability in *Arabidopsis* . J Biol Chem 283: 15932–15945. 18408011 10.1074/jbc.M801406200 PMC3259626

[59] McCaig BC, Meagher RB, Dean JF (2005) Gene structure and molecular analysis of the laccase-like multicopper oxidase (LMCO) gene family in *Arabidopsis thaliana* . Planta 221: 619–636. 15940465 10.1007/s00425-004-1472-6

[60] Ryden LG, Hunt LT (1993) Evolution of protein complexity: the blue copper-containing oxidases and related proteins. J Mol Evol 36: 41–66. 8433378 10.1007/BF02407305

[61] Cai X, Davis EJ, Ballif J, Liang M, Bushman E, et al (2006) Mutant identification and characterization of the laccase gene family in *Arabidopsis* . J Exp Bot 57: 2563–2569. 16804053 10.1093/jxb/erl022

[62] Xue LJ, Zhang JJ, Xue HW (2009) Characterization and expression profiles of miRNAs in rice seeds. Nucleic Acids Res 37: 916–930. 19103661 10.1093/nar/gkn998 PMC2647296

[63] Zhang J, Zhang S, Han S, Wu T, Li X, et al (2012) Genome-wide identification of microRNAs in larch and stage-specific modulation of 11 conserved microRNAs and their targets during somatic embryogenesis. Planta 236: 647–657. 22526500 10.1007/s00425-012-1643-9

[64] Vadassery J, Ritter C, Venus Y, Camehl I, Varma A, et al (2008) The role of auxins and cytokinins in the mutualistic interaction between *Arabidopsis* and *Piriformospora indica* . Mol Plant Microbe Interact 21: 1371–1383. 18785832 10.1094/MPMI-21-10-1371

[65] Guilfoyle TJ, Ulmasov T, Hagen G (1998) The ARF family of transcription factors and their role in plant hormone-responsive transcription. Cell Mol Life Sci 54: 619–627. 9711229 10.1007/s000180050190 PMC11147363

[66] Wang CY, Chen YQ, Liu Q (2011) Sculpting the meristem: the roles of miRNAs in plant stem cells. Biochem Biophys Res Commun 409: 363–366. 21570951 10.1016/j.bbrc.2011.04.123

[67] Elhiti M, Stasolla C (2009) Structure and function of homodomain-leucine zipper (HD-Zip) proteins. Plant Signal Behav 4: 86–88. 19649178 10.4161/psb.4.2.7692 PMC2637487

[68] Sun X, Wang GL (2011) Genome-wide identification, characterization and phylogenetic analysis of the rice LRR-kinases. PLoS ONE 6: e16079. 21408199 10.1371/journal.pone.0016079 PMC3050792

[69] Xu G, Ma H, Nei M, Kong H (2009) Evolution of F-box genes in plants: different modes of sequence divergence and their relationships with functional diversification. Proc Natl Acad Sci U S A 106: 835–840. 19126682 10.1073/pnas.0812043106 PMC2630105

[70] Ramirez SR, Gravendeel B, Singer RB, Marshall CR, Pierce NE (2007) Dating the origin of the Orchidaceae from a fossil orchid with its pollinator. Nature 448: 1042–1045. 17728756 10.1038/nature06039

[71] Hu HY GS, Xi J, Yan Z, Fu N, Zhang X, Menzel C, Liang H, Yang H, Zhao M, Zeng R, Chen W, Pääbo S, Khaitovich P (2011) MicroRNA expression and regulation in human, chimpanzee, and macaque brains. PLoS genetics 7: e1002327. 22022286 10.1371/journal.pgen.1002327 PMC3192836

[72] Cuperus JT, Fahlgren N, Carrington JC (2011) Evolution and functional diversification of *MIRNA* genes. Plant Cell 23: 431–442. 21317375 10.1105/tpc.110.082784 PMC3077775

[73] An FM, Hsiao SR, Chan MT (2011) Sequencing-based approaches reveal low ambient temperature-responsive and tissue-specific microRNAs in *phalaenopsis* orchid. PLoS ONE 6: e18937. 21573107 10.1371/journal.pone.0018937 PMC3089612

[74] Montgomery TA, Howell MD, Cuperus JT, Li D, Hansen JE, et al (2008) Specificity of ARGONAUTE7-miR390 interaction and dual functionality in *TAS3* trans-acting siRNA formation. Cell 133: 128–141. 18342362 10.1016/j.cell.2008.02.033

[75] Sunkar R, Girke T, Jain PK, Zhu JK (2005) Cloning and characterization of microRNAs from rice. Plant Cell 17: 1397–1411. 15805478 10.1105/tpc.105.031682 PMC1091763

[76] Khatabi B, Molitor A, Lindermayr C, Pfiffi S, Durner J, et al (2012) Ethylene supports colonization of plant roots by the mutualistic fungus *Piriformospora indica* . PLoS ONE 7: e35502. 22536394 10.1371/journal.pone.0035502 PMC3334895

[77] Camehl I, Oelmüller R (2010) Do ethylene response factorS9 and −14 repress *PR* gene expression in the interaction between *Piriformospora indica* and *Arabidopsis*? Plant Signal Behav 5: 932–936. 20505369 10.4161/psb.5.8.12036 PMC3115165

[78] Navarro L, Bari R, Achard P, Lison P, Nemri A, et al (2008) DELLAs control plant immune responses by modulating the balance of jasmonic acid and salicylic acid signaling. Curr Biol 18: 650–655. 18450451 10.1016/j.cub.2008.03.060

[79] Nagpal P, Ellis CM, Weber H, Ploense SE, Barkawi LS, et al (2005) Auxin response factors ARF6 and ARF8 promote jasmonic acid production and flower maturation. Development 132: 4107–4118. 16107481 10.1242/dev.01955

[80] Wang D, Pajerowska-Mukhtar K, Culler AH, Dong X (2007) Salicylic acid inhibits pathogen growth in plants through repression of the auxin signaling pathway. Curr Biol 17: 1784–1790. 17919906 10.1016/j.cub.2007.09.025

[81] Zhang Z, Zhang X (2012) Argonautes compete for miR165/166 to regulate shoot apical meristem development. Curr Opin Plant Biol 15: 652–658. 22727764 10.1016/j.pbi.2012.05.007 PMC3460042

[82] Xie Z, Kasschau KD, Carrington JC (2003) Negative feedback regulation of *Dicer-Like1* in *Arabidopsis* by microRNA-guided mRNA degradation. Curr Biol 13: 784–789. 12725739 10.1016/s0960-9822(03)00281-1

[83] Vaucheret H, Vazquez F, Crete P, Bartel DP (2004) The action of *ARGONAUTE1* in the miRNA pathway and its regulation by the miRNA pathway are crucial for plant development. Genes Dev 18: 1187–1197. 15131082 10.1101/gad.1201404 PMC415643

[84] Kidner CA, Martienssen RA (2005) The role of ARGONAUTE1 (AGO1) in meristem formation and identity. Dev Biol 280: 504–517. 15882589 10.1016/j.ydbio.2005.01.031

[85] Zhou GK, Kubo M, Zhong R, Demura T, Ye ZH (2007) Overexpression of miR165 affects apical meristem formation, organ polarity establishment and vascular development in *Arabidopsis* . Plant Cell Physiol 48: 391–404. 17237362 10.1093/pcp/pcm008

[86] Ling LZ, Zhang SD (2012) Exploring the evolutionary differences of SBP-box genes targeted by *miR156* and *miR529* in plants. Genetica 140: 317–324. 23054224 10.1007/s10709-012-9684-3

[87] Li WX, Oono Y, Zhu J, He XJ, Wu JM, et al (2008) The *Arabidopsis* NFYA5 transcription factor is regulated transcriptionally and posttranscriptionally to promote drought resistance. Plant Cell 20: 2238–2251. 18682547 10.1105/tpc.108.059444 PMC2553615

[88] Zuccaro A, Lahrmann U, Guldener U, Langen G, Pfiffi S, et al (2011) Endophytic life strategies decoded by genome and transcriptome analyses of the mutualistic root symbiont *Piriformospora indica* . PLoS Path 7: e1002290. 10.1371/journal.ppat.1002290 PMC3192844 22022265

