# Supplementary material for: Growth Promotion-Related miRNAs in Oncidium Orchid Roots Colonized by the Endophytic Fungus Piriformospora indica
Source: PLoS One. 2014 Jan 7;9(1):e84920. doi: 10.1371/journal.pone.0084920 (PMC3883679; doi:10.1371/journal.pone.0084920)
Supplement: Figure S1 — Length distribution of small RNA. (PPTX) [file pone.0084920.s007.pptx]

## Slide 1
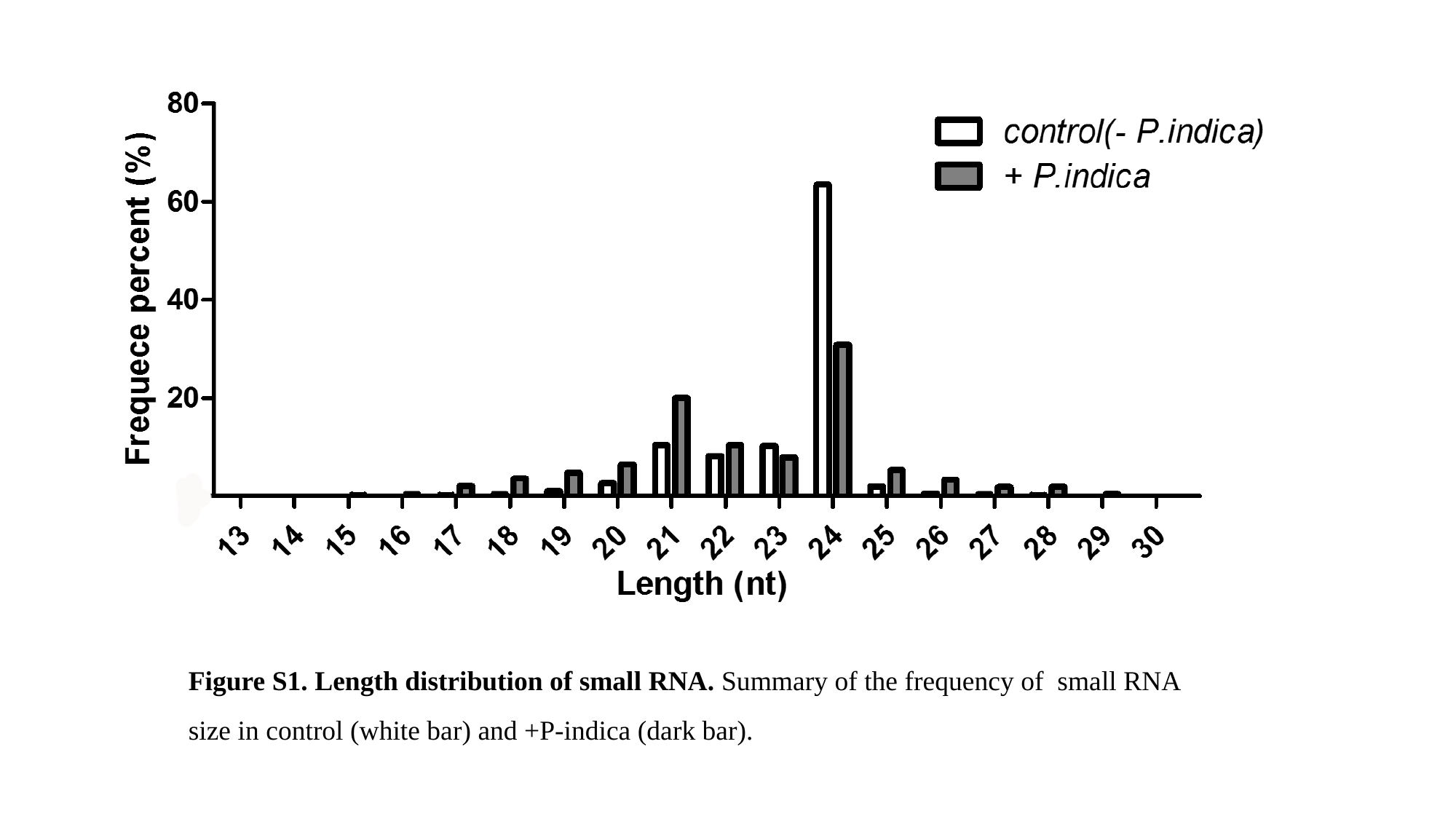

Figure S1. Length distribution of small RNA. Summary of the frequency of small RNA size in control (white bar) and +P-indica (dark bar).
