# Supplementary material for: Growth Promotion-Related miRNAs in Oncidium Orchid Roots Colonized by the Endophytic Fungus Piriformospora indica
Source: PLoS One. 2014 Jan 7;9(1):e84920. doi: 10.1371/journal.pone.0084920 (PMC3883679; doi:10.1371/journal.pone.0084920)
Supplement: Figure S2 — Distribution of plant species conserved miRNA homology. (PPTX) [file pone.0084920.s008.pptx]

## Slide 1
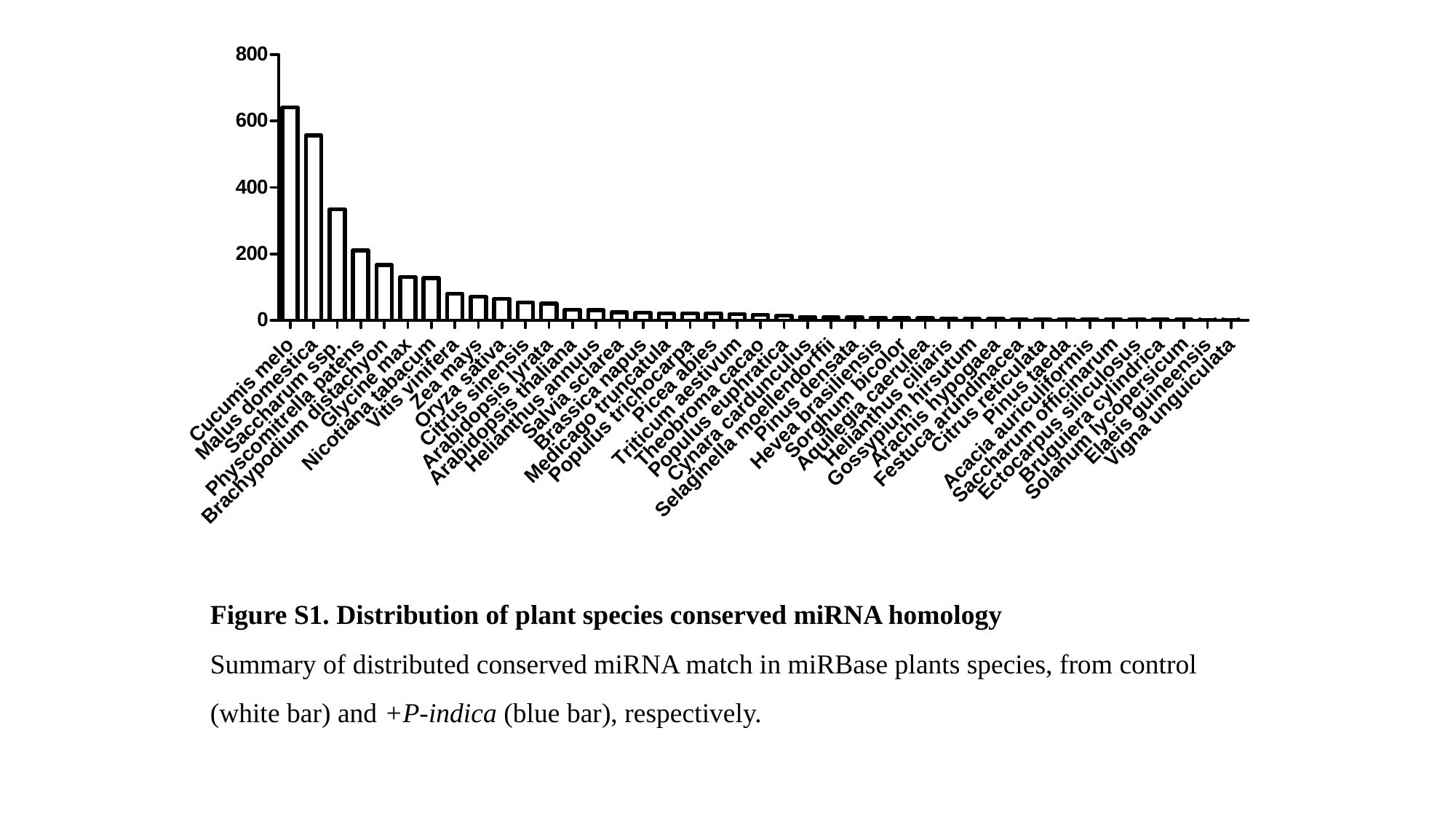

Figure S1. Distribution of plant species conserved miRNA homology
Summary of distributed conserved miRNA match in miRBase plants species, from control (white bar) and +P-indica (blue bar), respectively.
