# Supplementary material for: Growth Promotion-Related miRNAs in Oncidium Orchid Roots Colonized by the Endophytic Fungus Piriformospora indica
Source: PLoS One. 2014 Jan 7;9(1):e84920. doi: 10.1371/journal.pone.0084920 (PMC3883679; doi:10.1371/journal.pone.0084920)
Supplement: Figure S3 — Frequency of the first nucleotide of the conserved miRNA. (PPTX) [file pone.0084920.s009.pptx]

## Slide 1
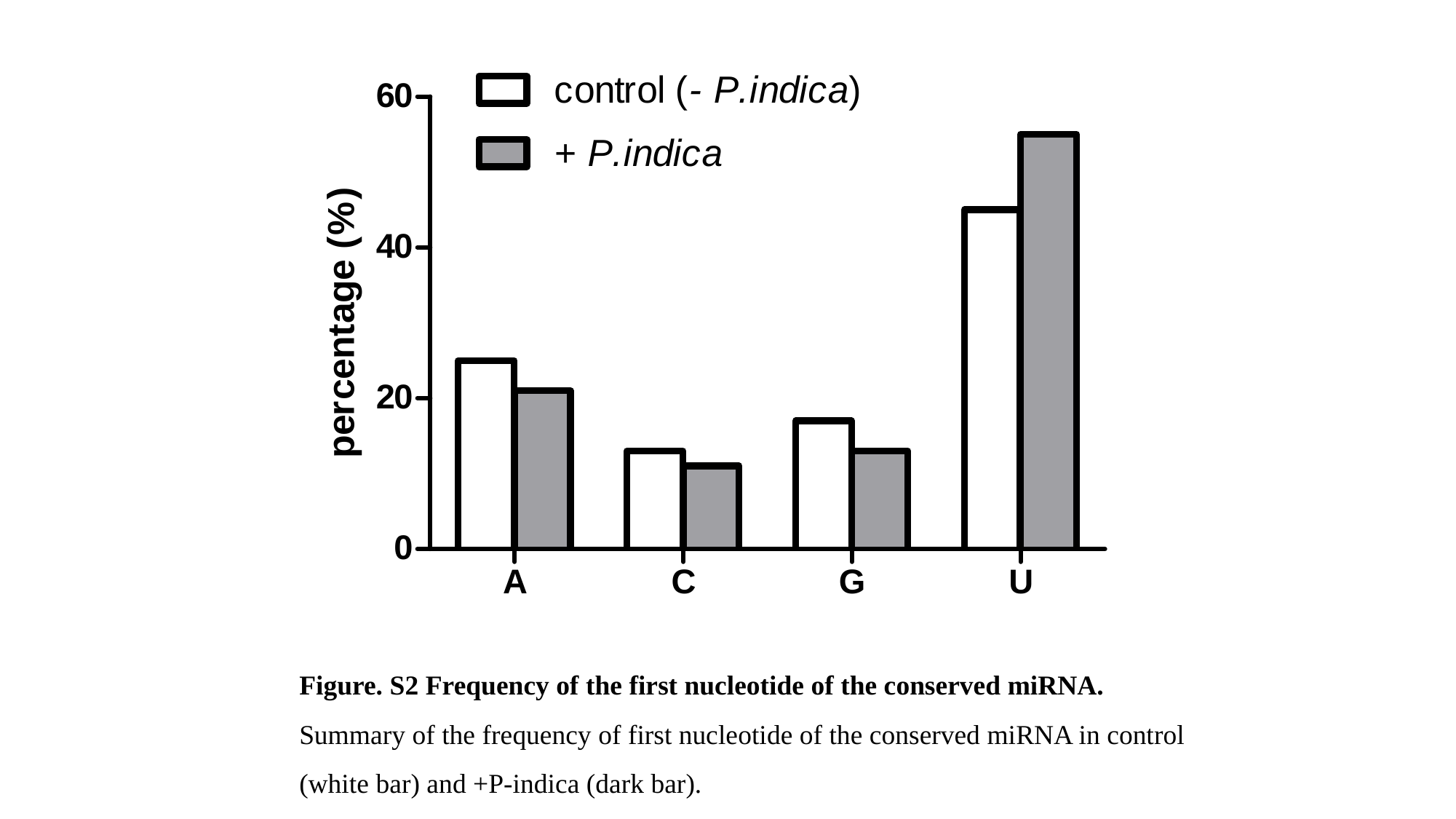

Figure. S2 Frequency of the first nucleotide of the conserved miRNA. Summary of the frequency of first nucleotide of the conserved miRNA in control (white bar) and +P-indica (dark bar).
