# Supplementary material for: Growth Promotion-Related miRNAs in Oncidium Orchid Roots Colonized by the Endophytic Fungus Piriformospora indica
Source: PLoS One. 2014 Jan 7;9(1):e84920. doi: 10.1371/journal.pone.0084920 (PMC3883679; doi:10.1371/journal.pone.0084920)
Supplement: Figure S4 — Secondary structure of miRNA precursor. (PPTX) [file pone.0084920.s010.pptx]

## Slide 1
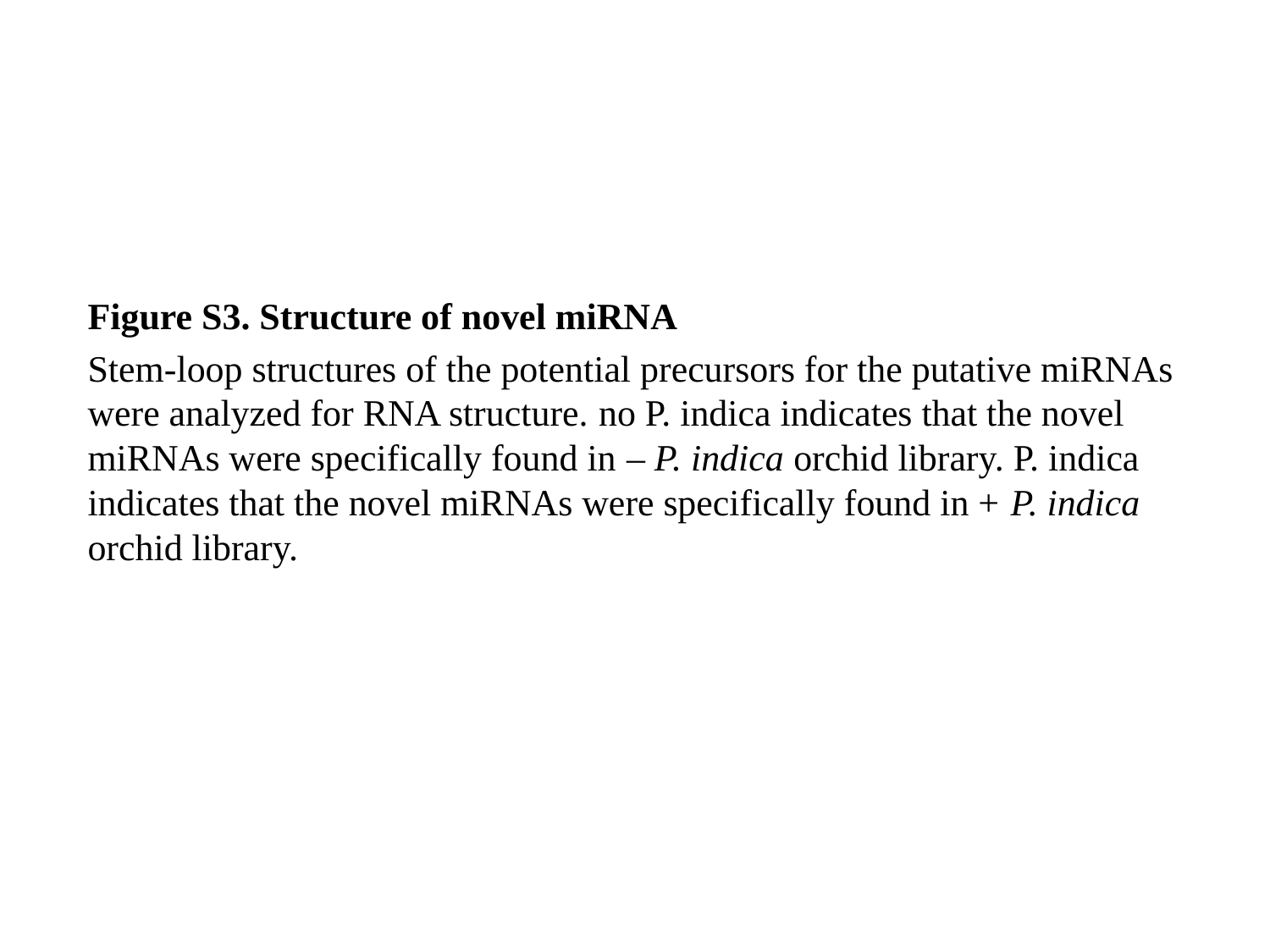

Figure S3. Structure of novel miRNA
Stem-loop structures of the potential precursors for the putative miRNAs were analyzed for RNA structure. no P. indica indicates that the novel miRNAs were specifically found in – P. indica orchid library. P. indica indicates that the novel miRNAs were specifically found in + P. indica orchid library.

## Slide 2
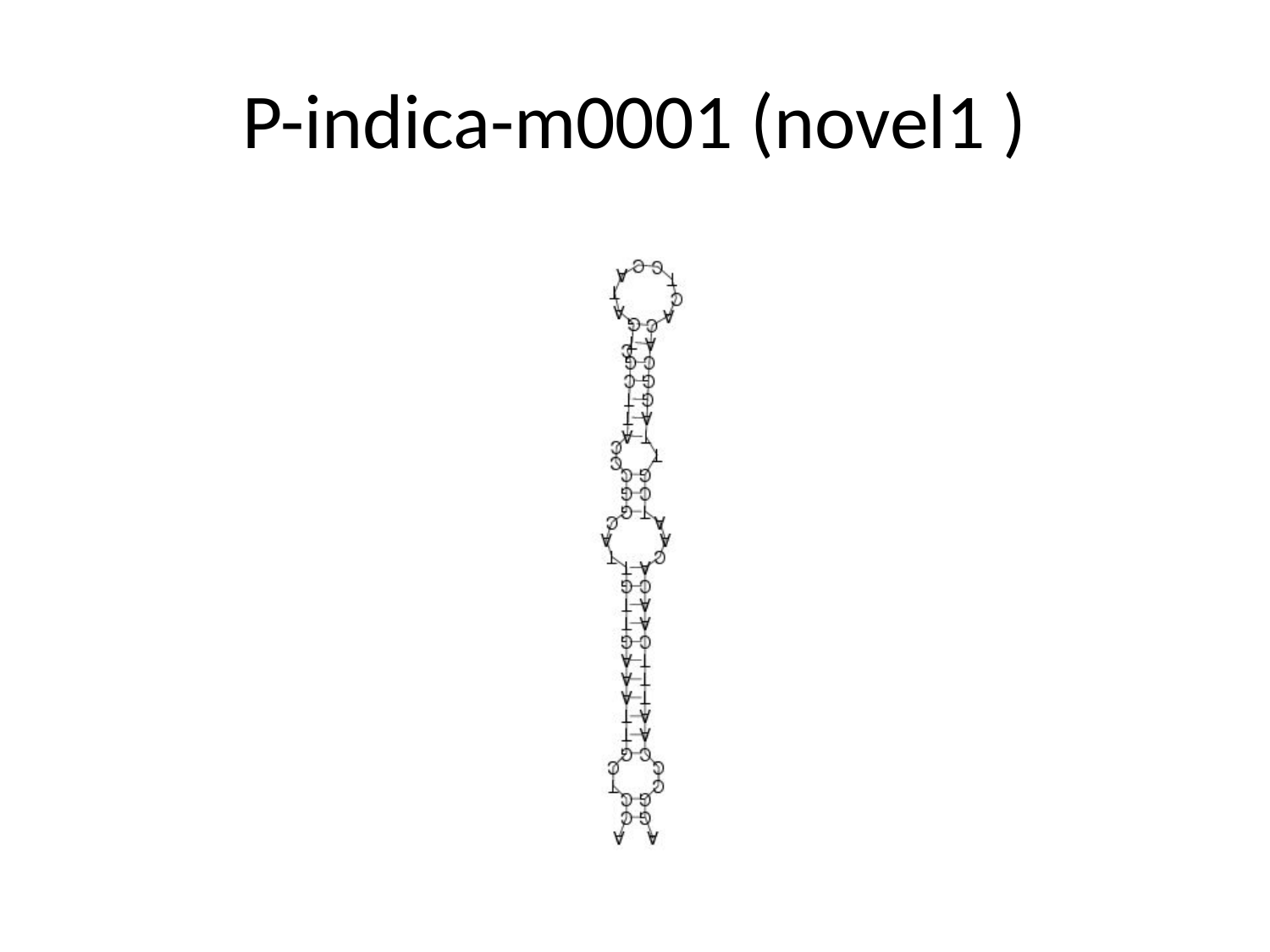

# P-indica-m0001 (novel1 )

## Slide 3
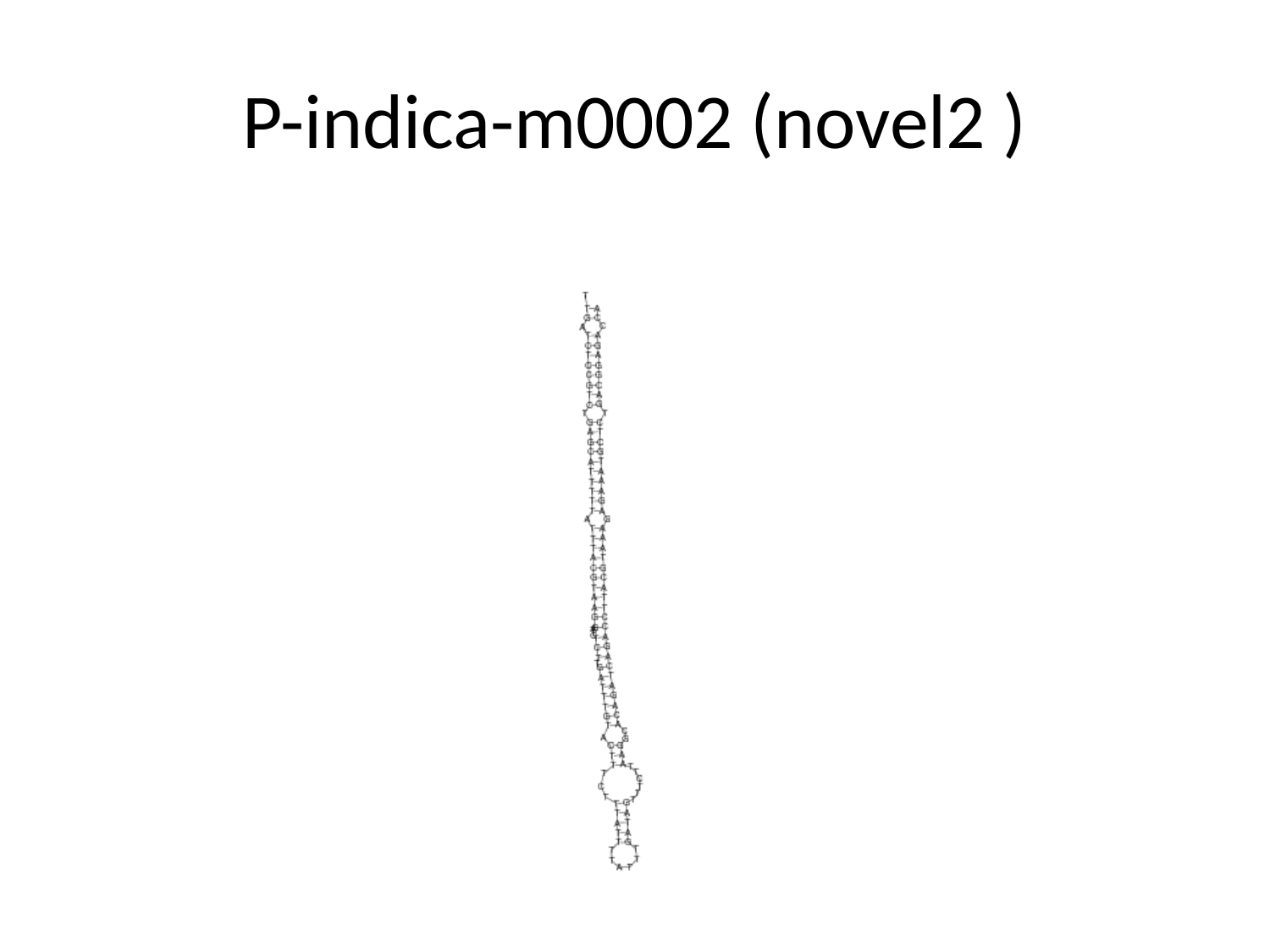

# P-indica-m0002 (novel2 )

## Slide 4
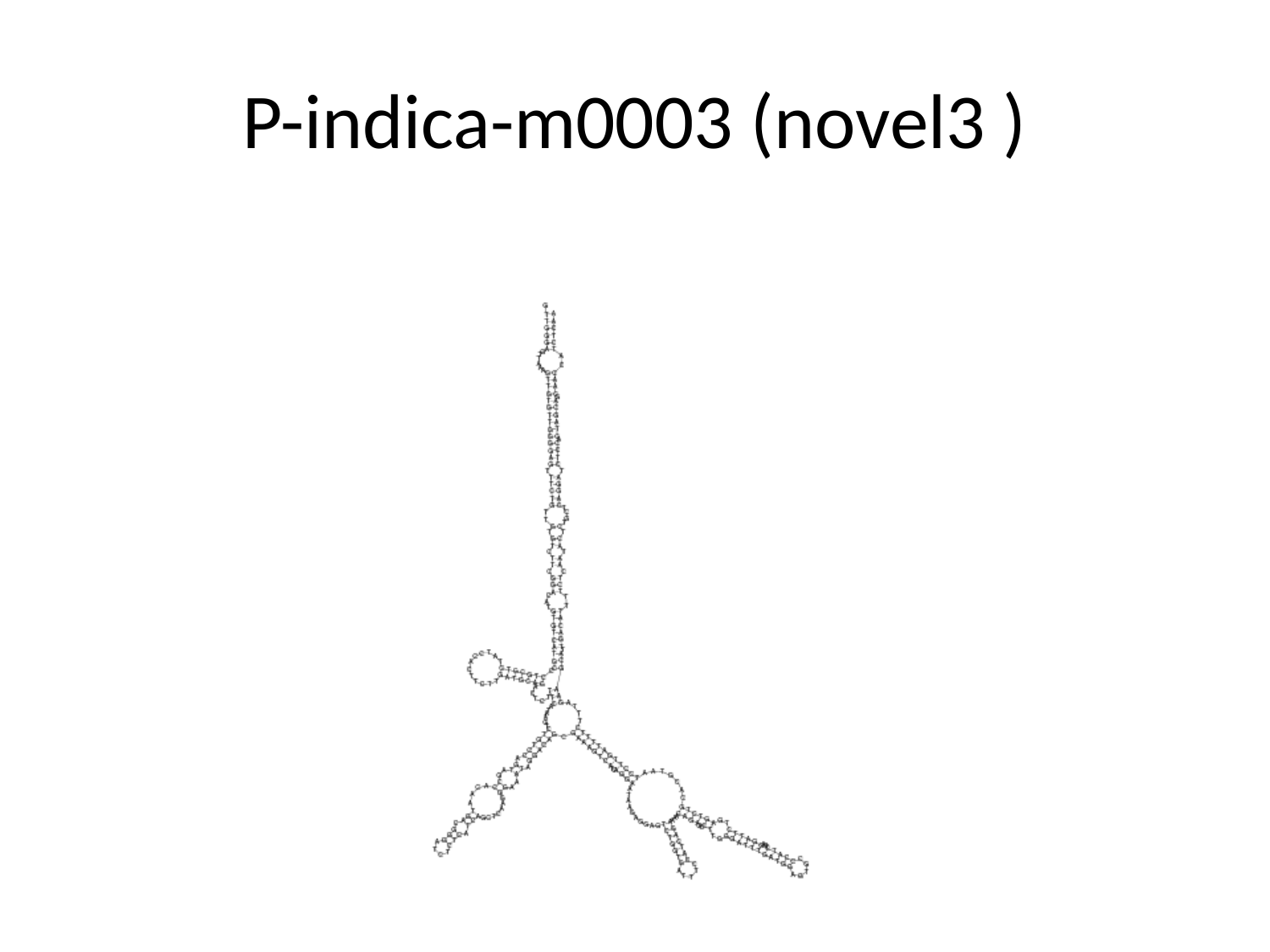

# P-indica-m0003 (novel3 )

## Slide 5
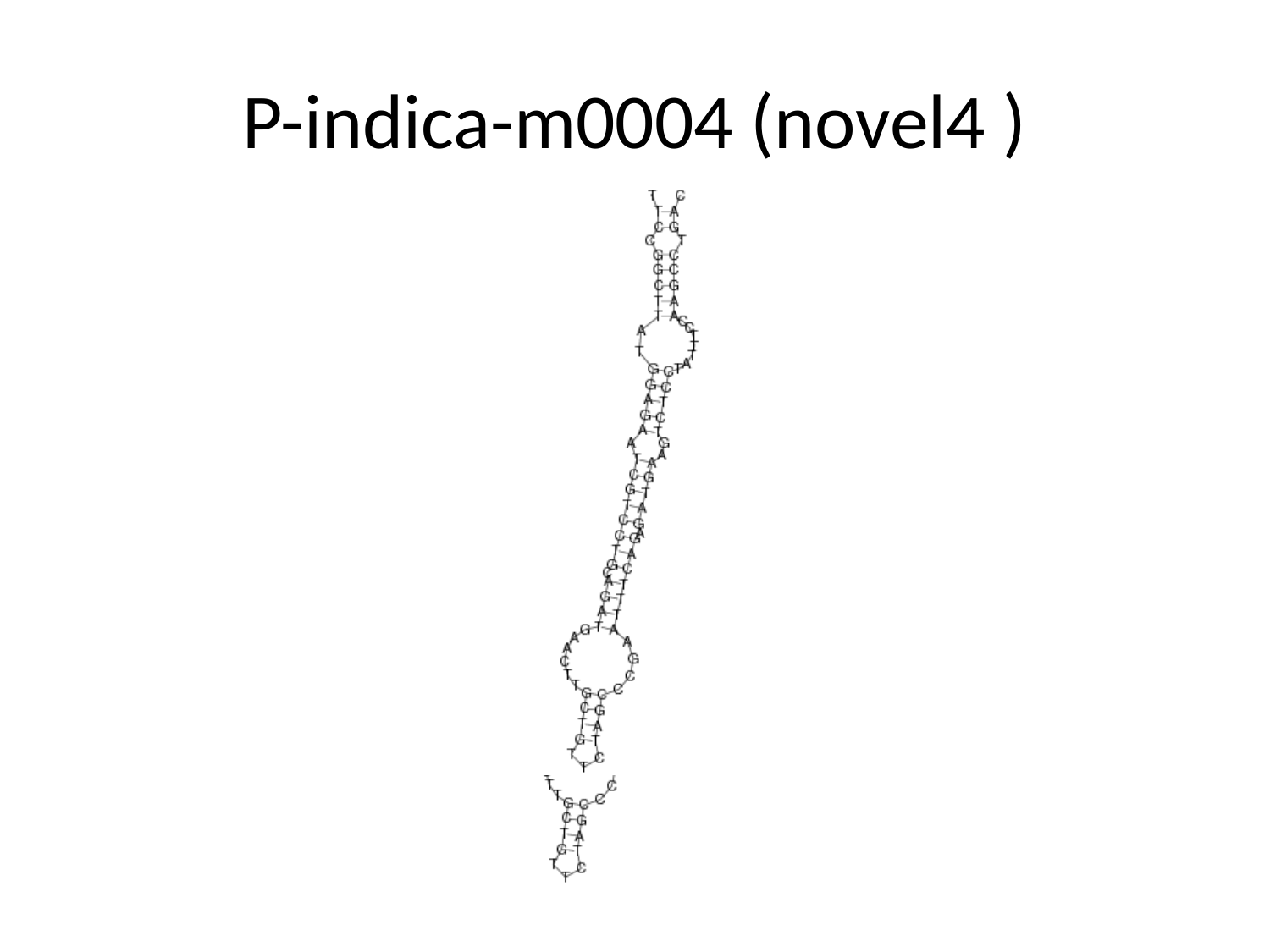

# P-indica-m0004 (novel4 )

## Slide 6
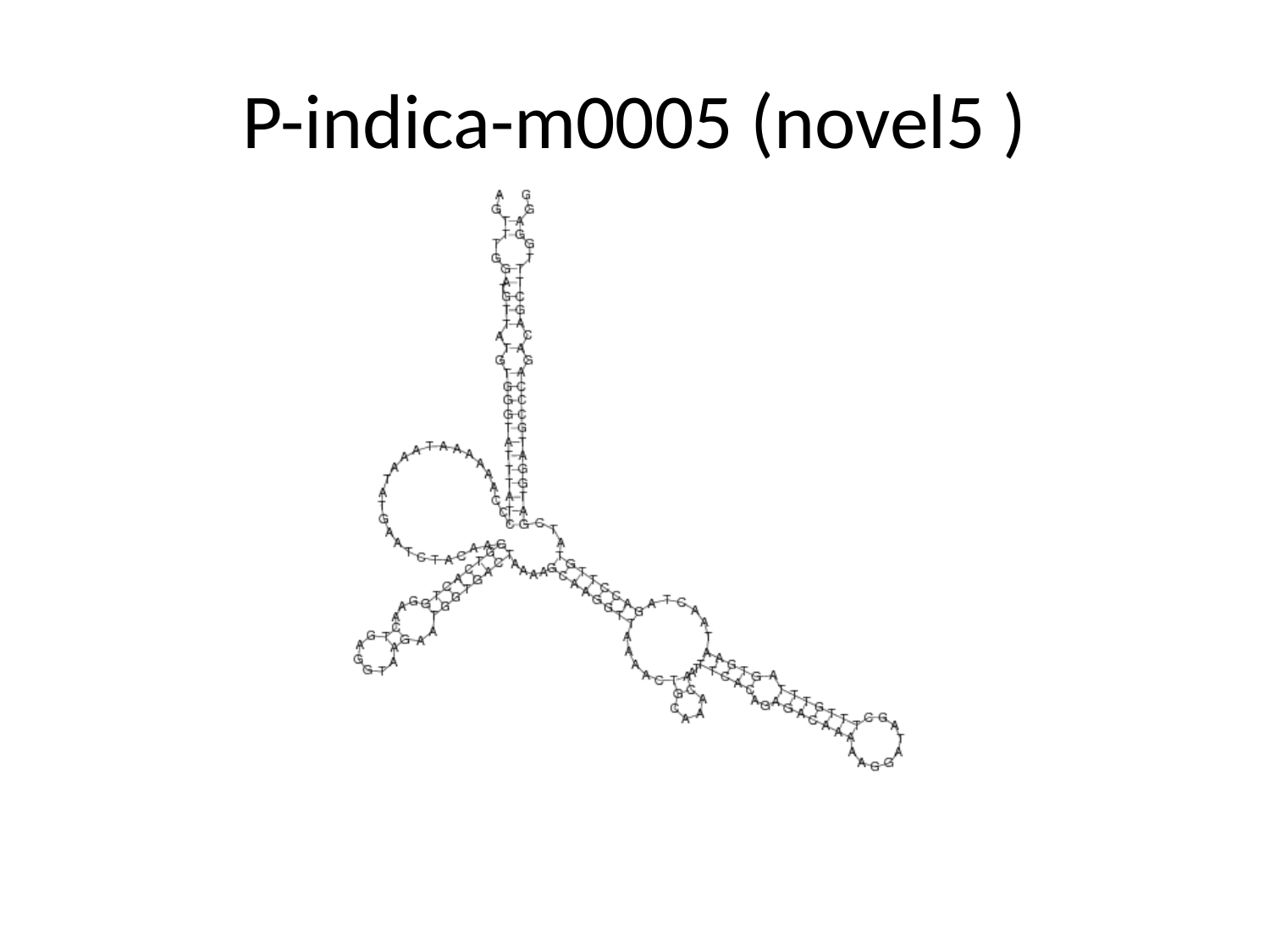

# P-indica-m0005 (novel5 )

## Slide 7
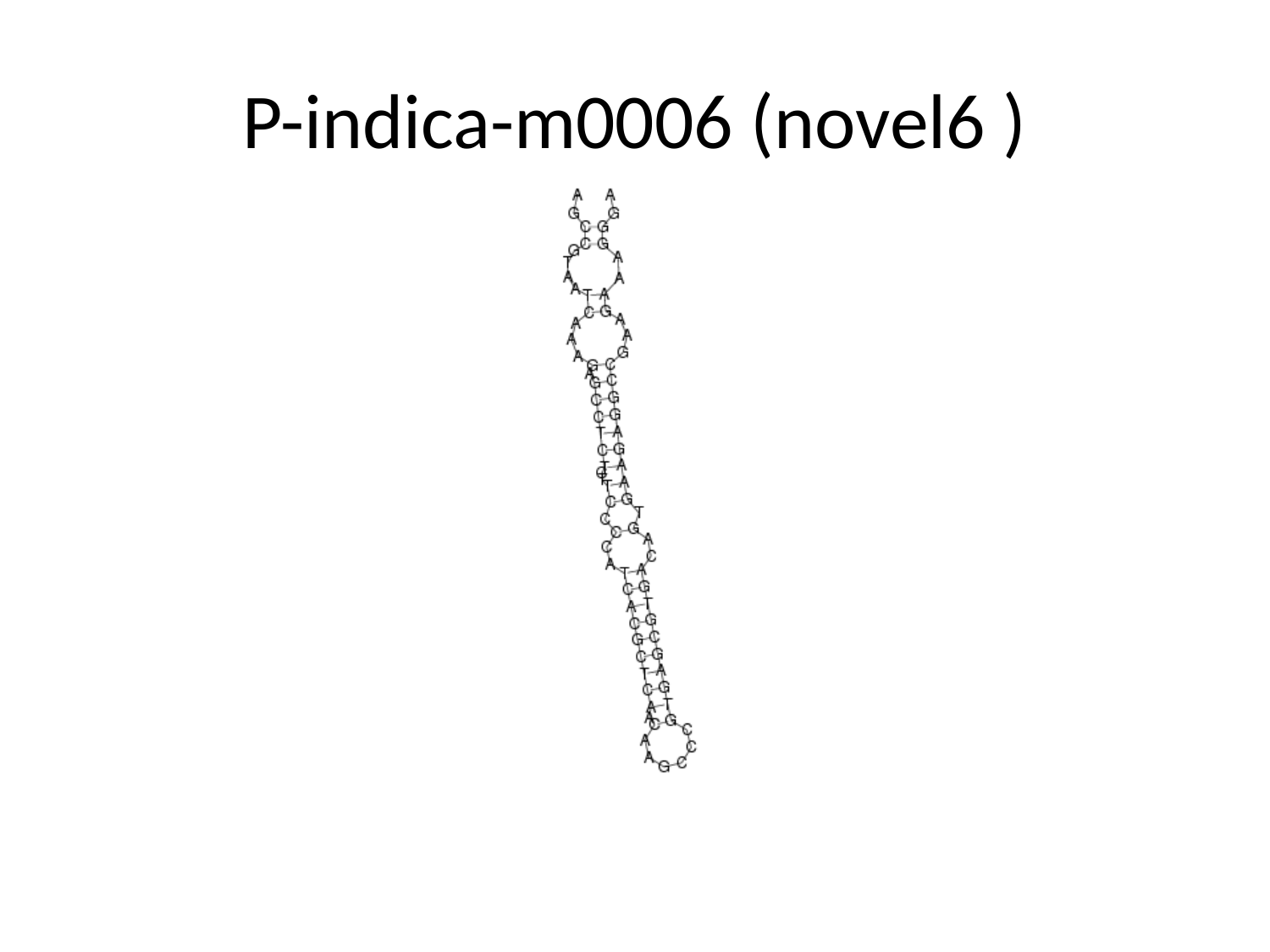

# P-indica-m0006 (novel6 )

## Slide 8
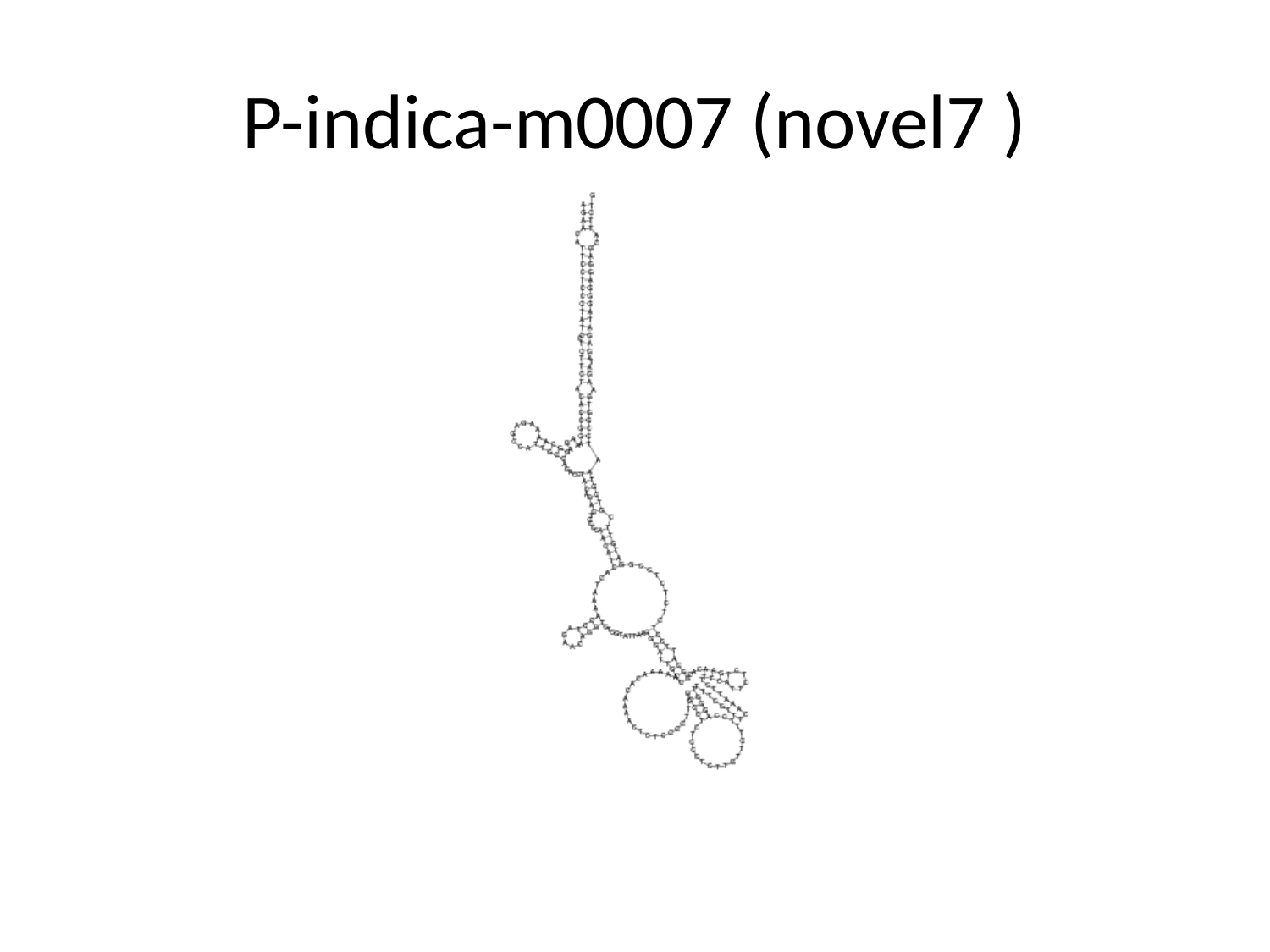

# P-indica-m0007 (novel7 )

## Slide 9
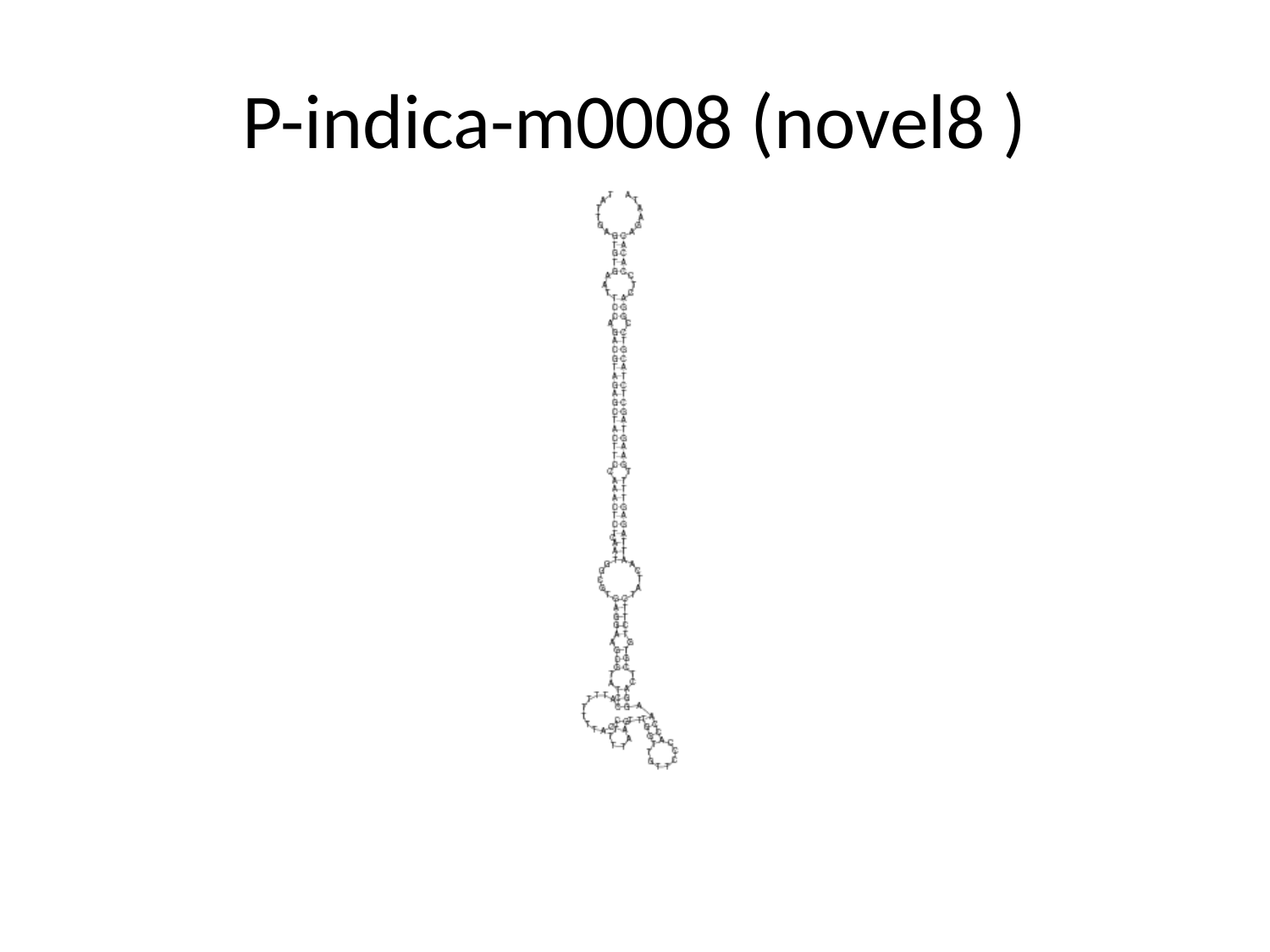

# P-indica-m0008 (novel8 )

## Slide 10
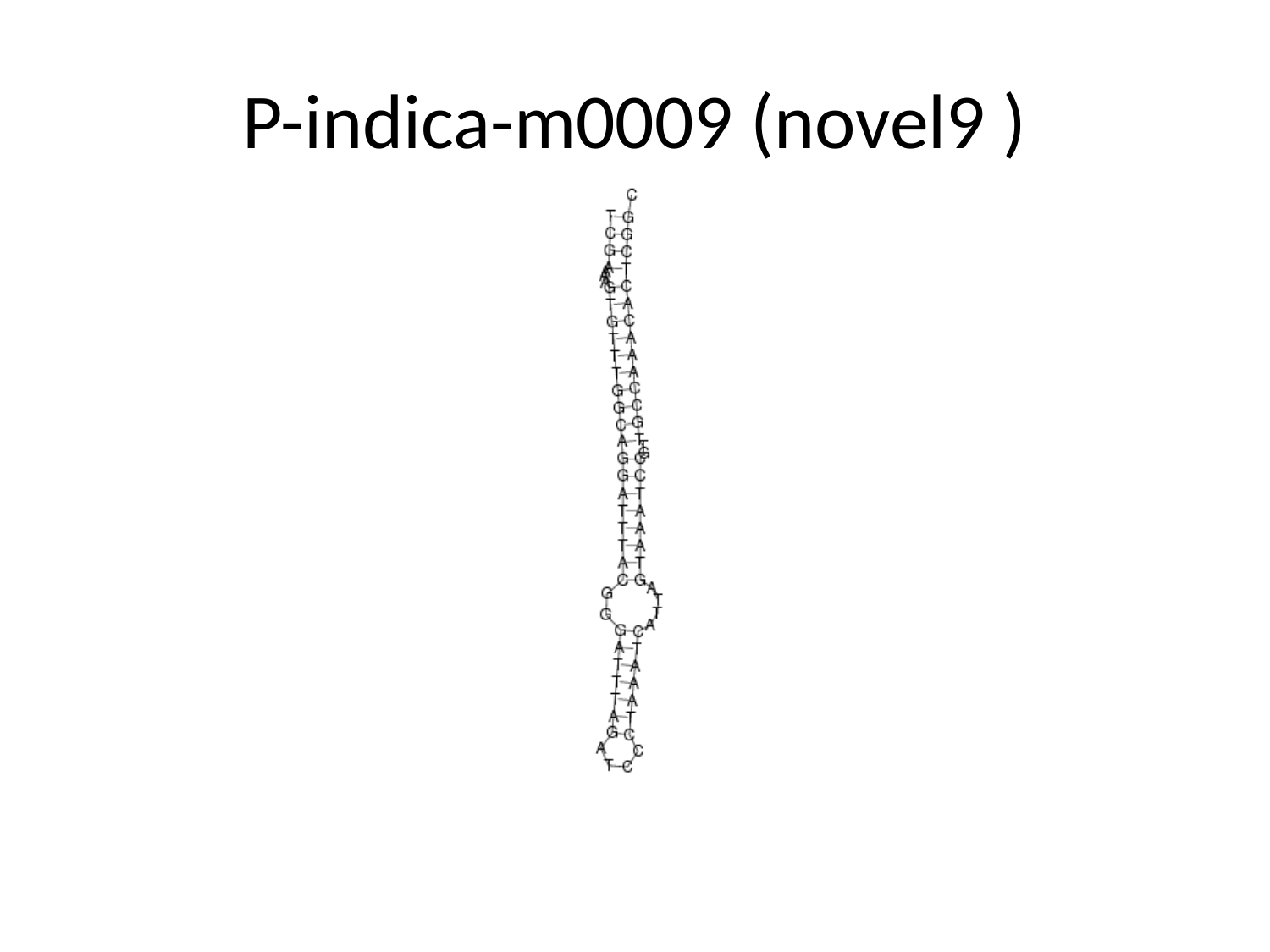

# P-indica-m0009 (novel9 )

## Slide 11
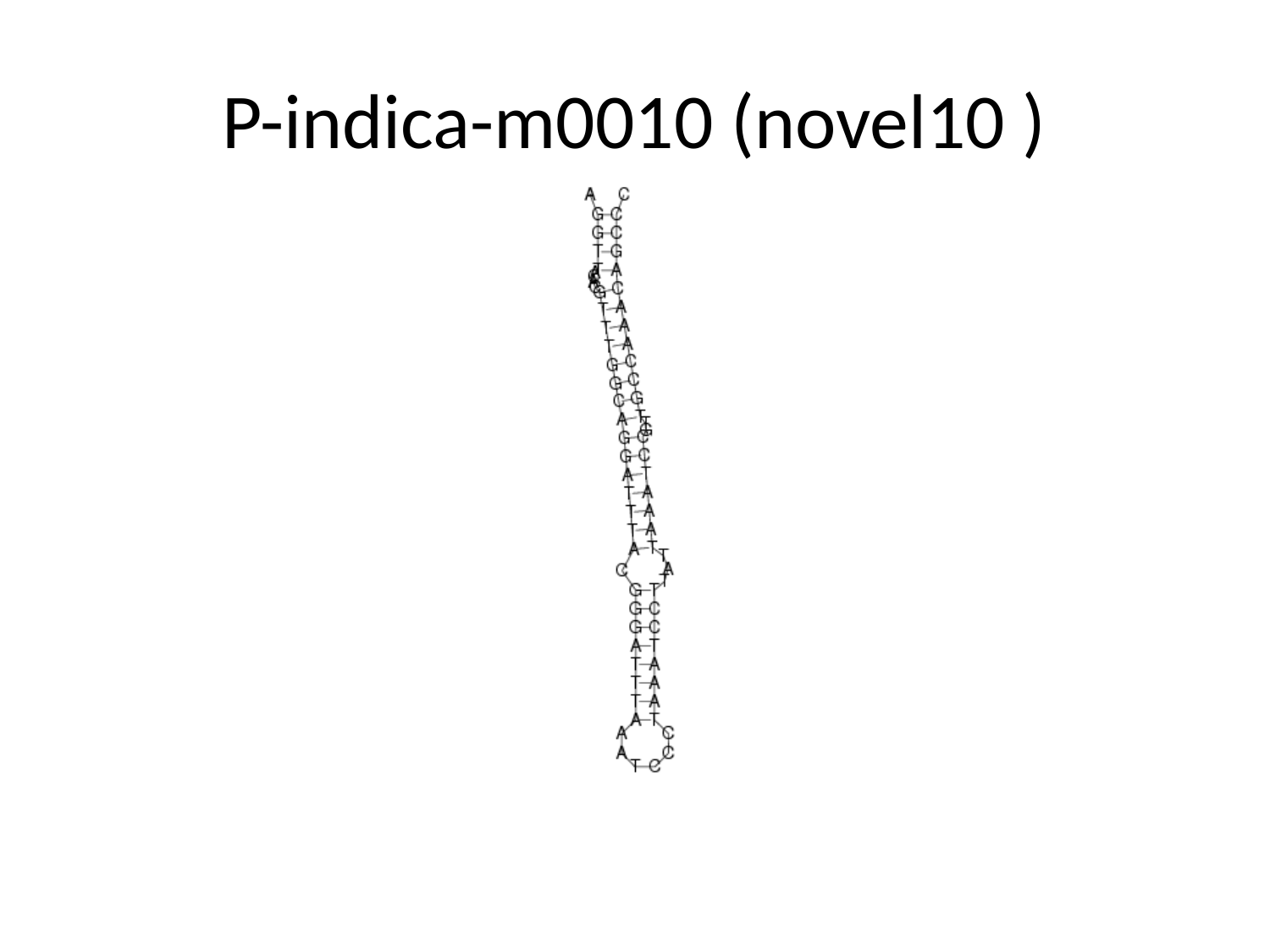

# P-indica-m0010 (novel10 )

## Slide 12
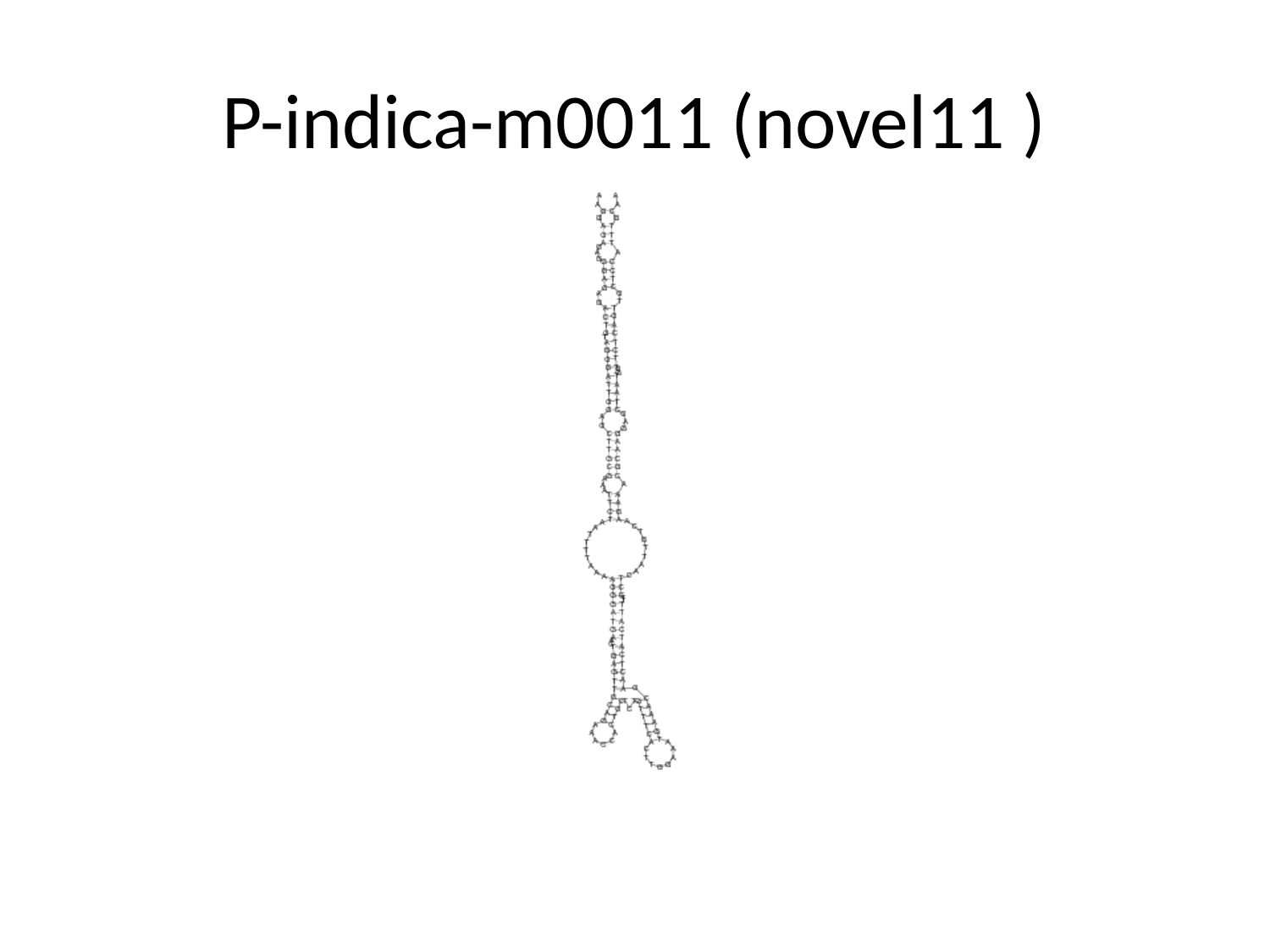

# P-indica-m0011 (novel11 )

## Slide 13
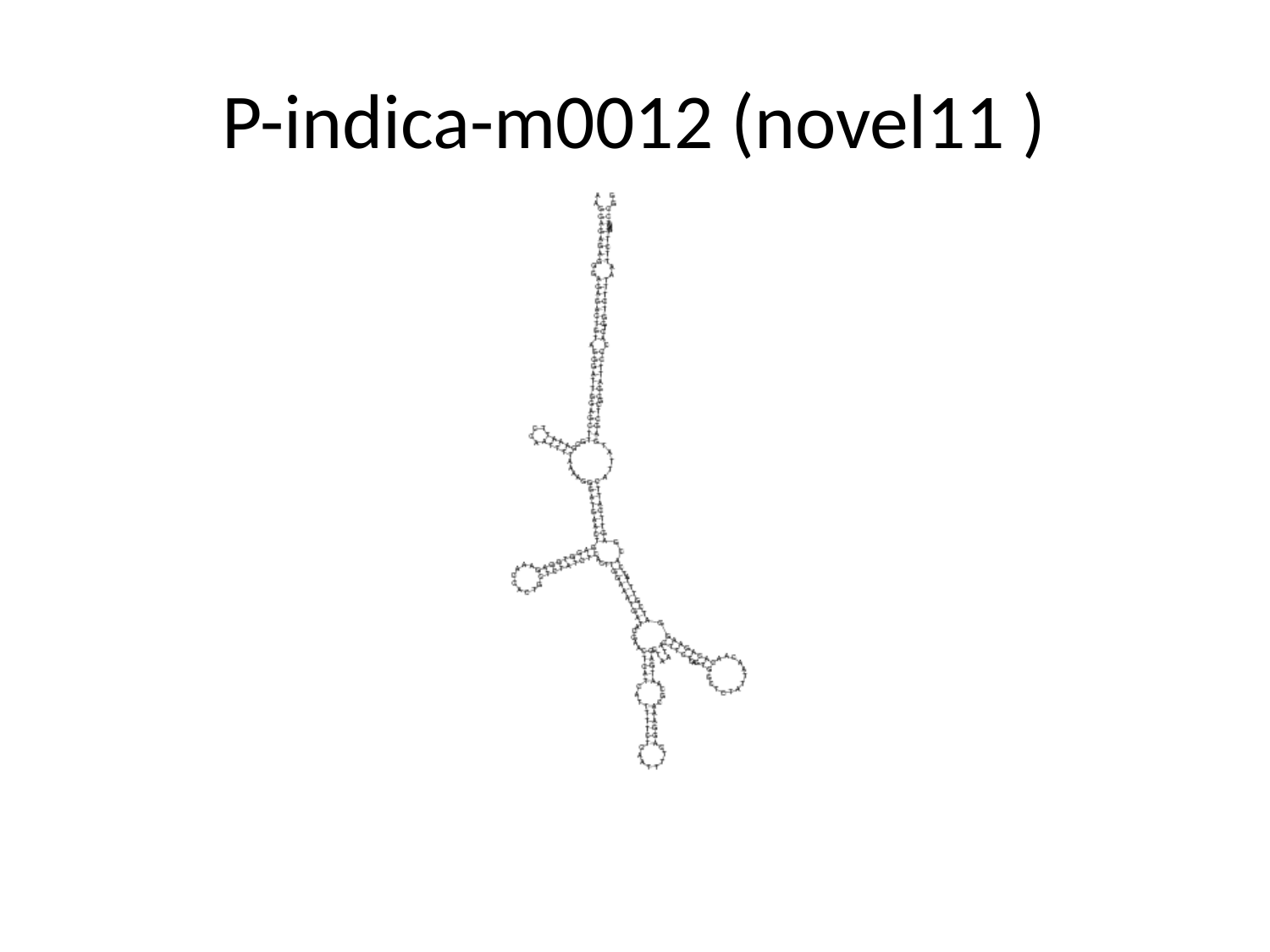

# P-indica-m0012 (novel11 )

## Slide 14
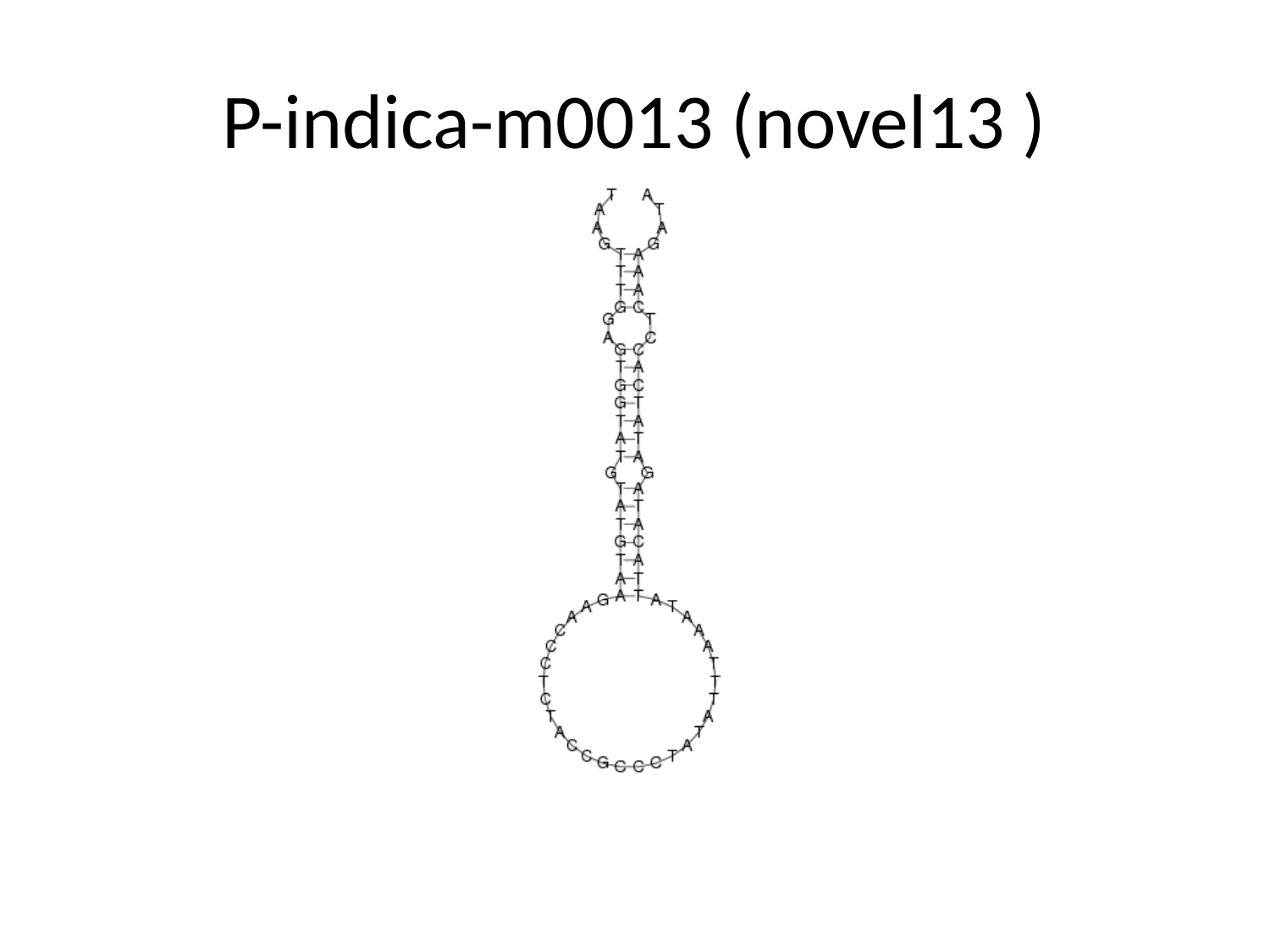

# P-indica-m0013 (novel13 )

## Slide 15
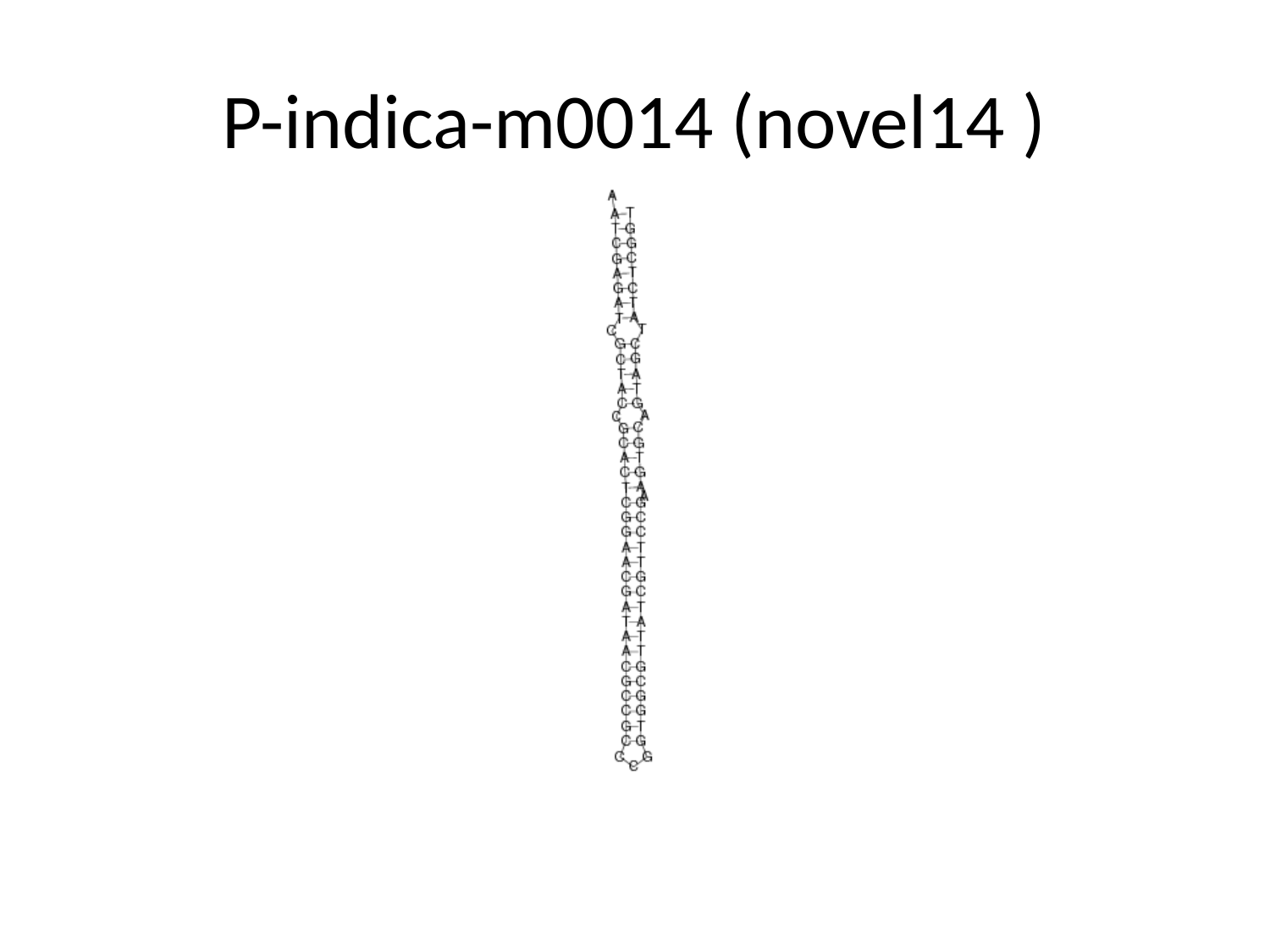

# P-indica-m0014 (novel14 )

## Slide 16
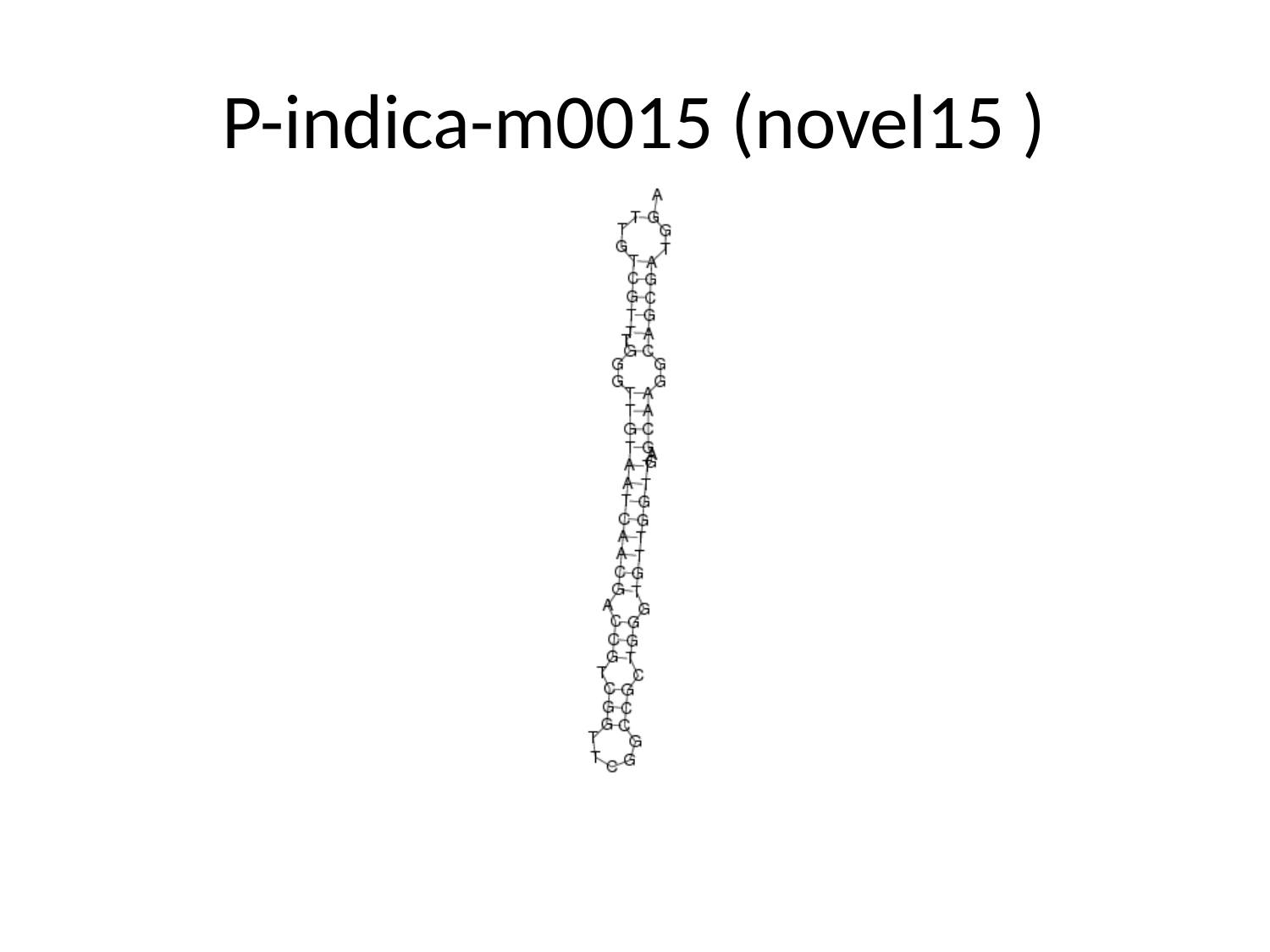

# P-indica-m0015 (novel15 )

## Slide 17
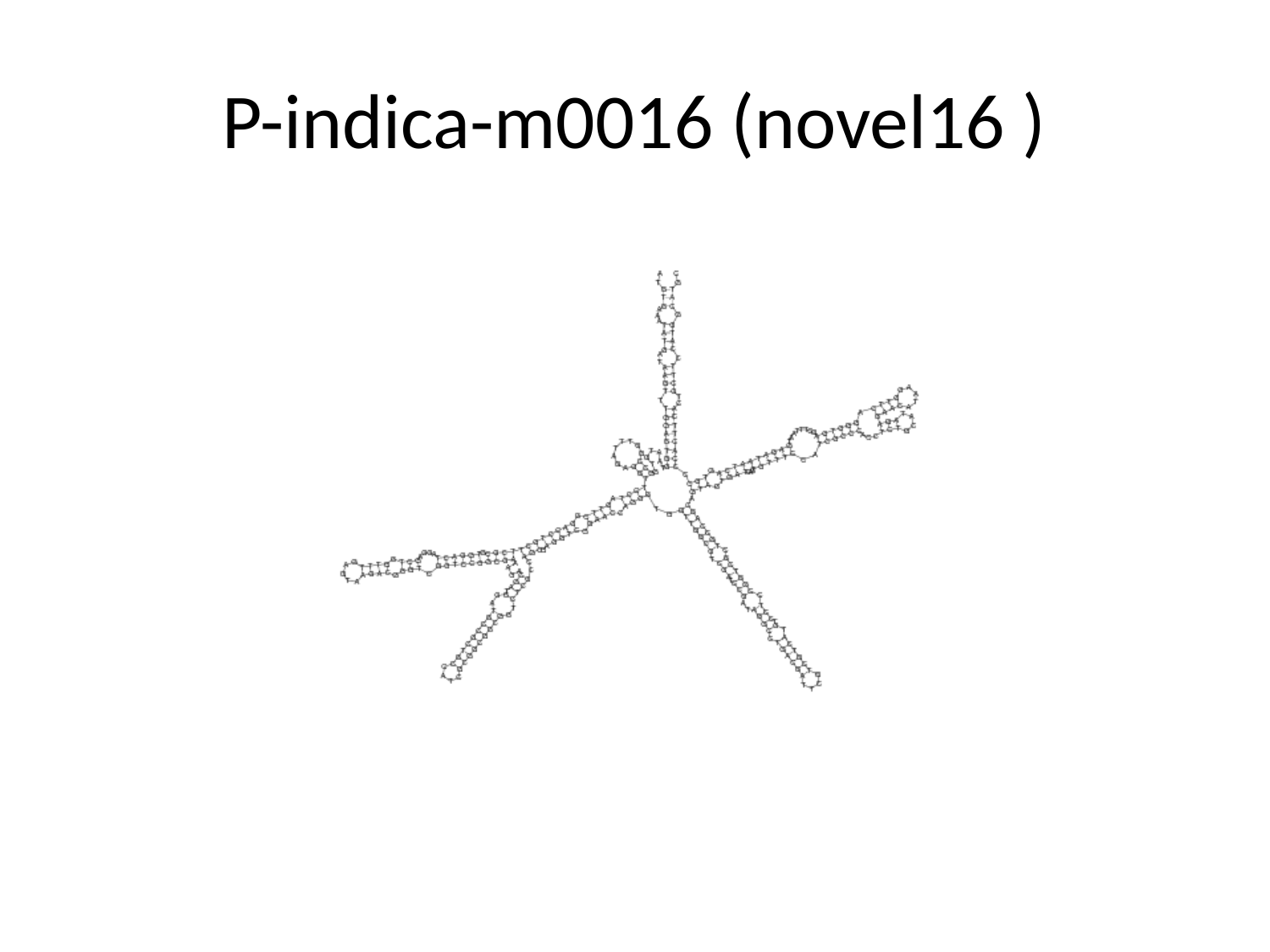

# P-indica-m0016 (novel16 )

## Slide 18
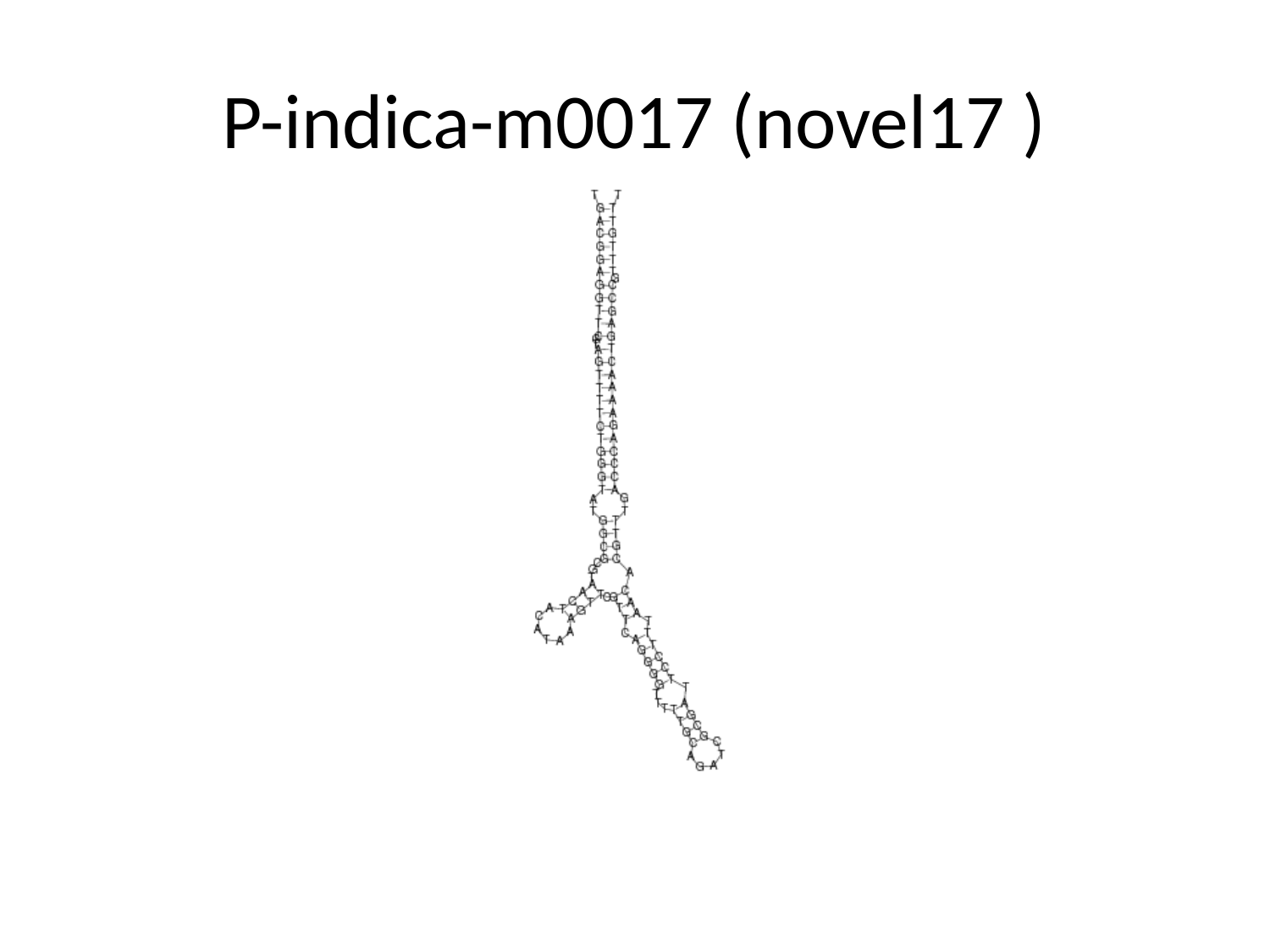

# P-indica-m0017 (novel17 )

## Slide 19
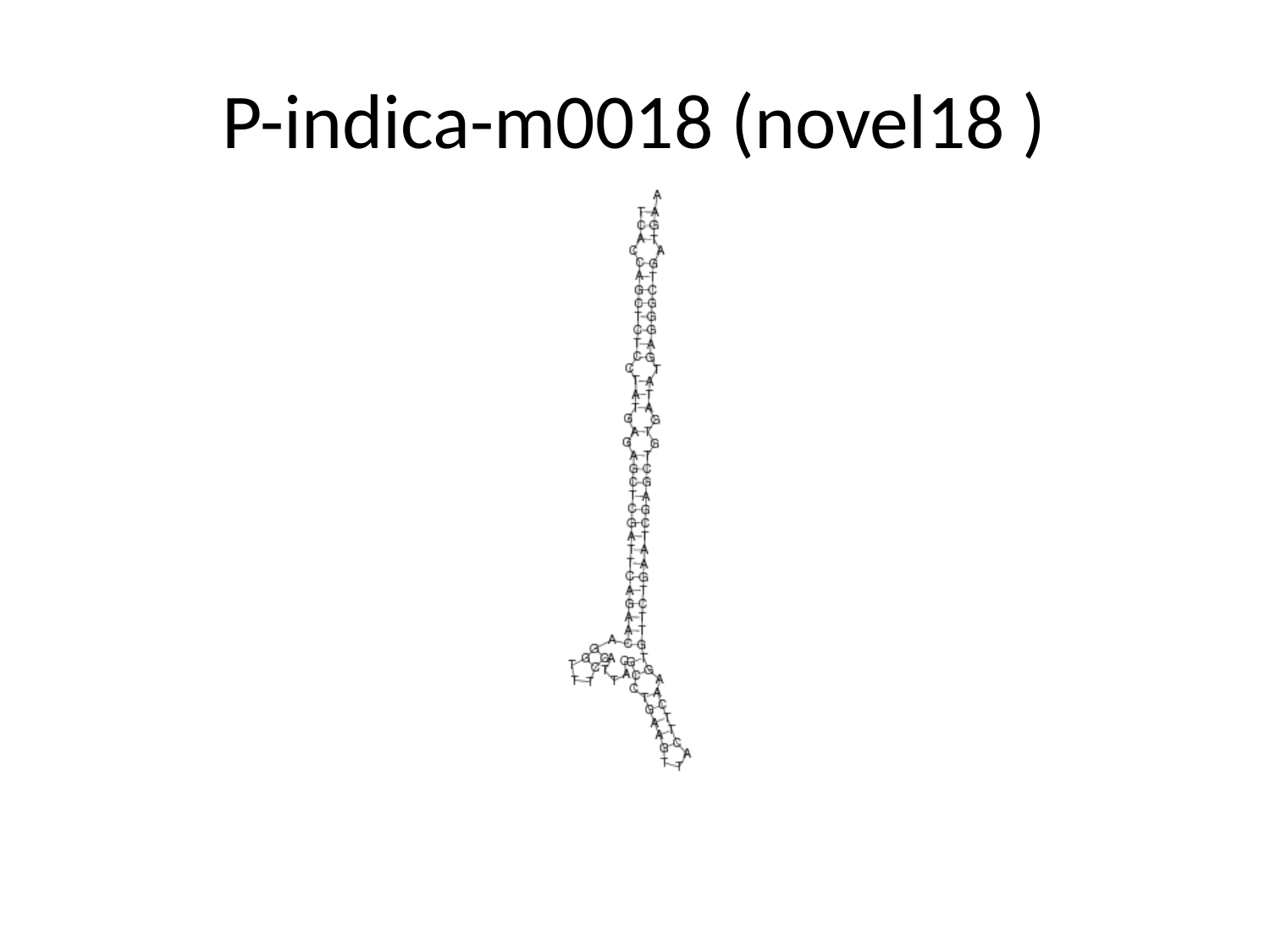

# P-indica-m0018 (novel18 )

## Slide 20
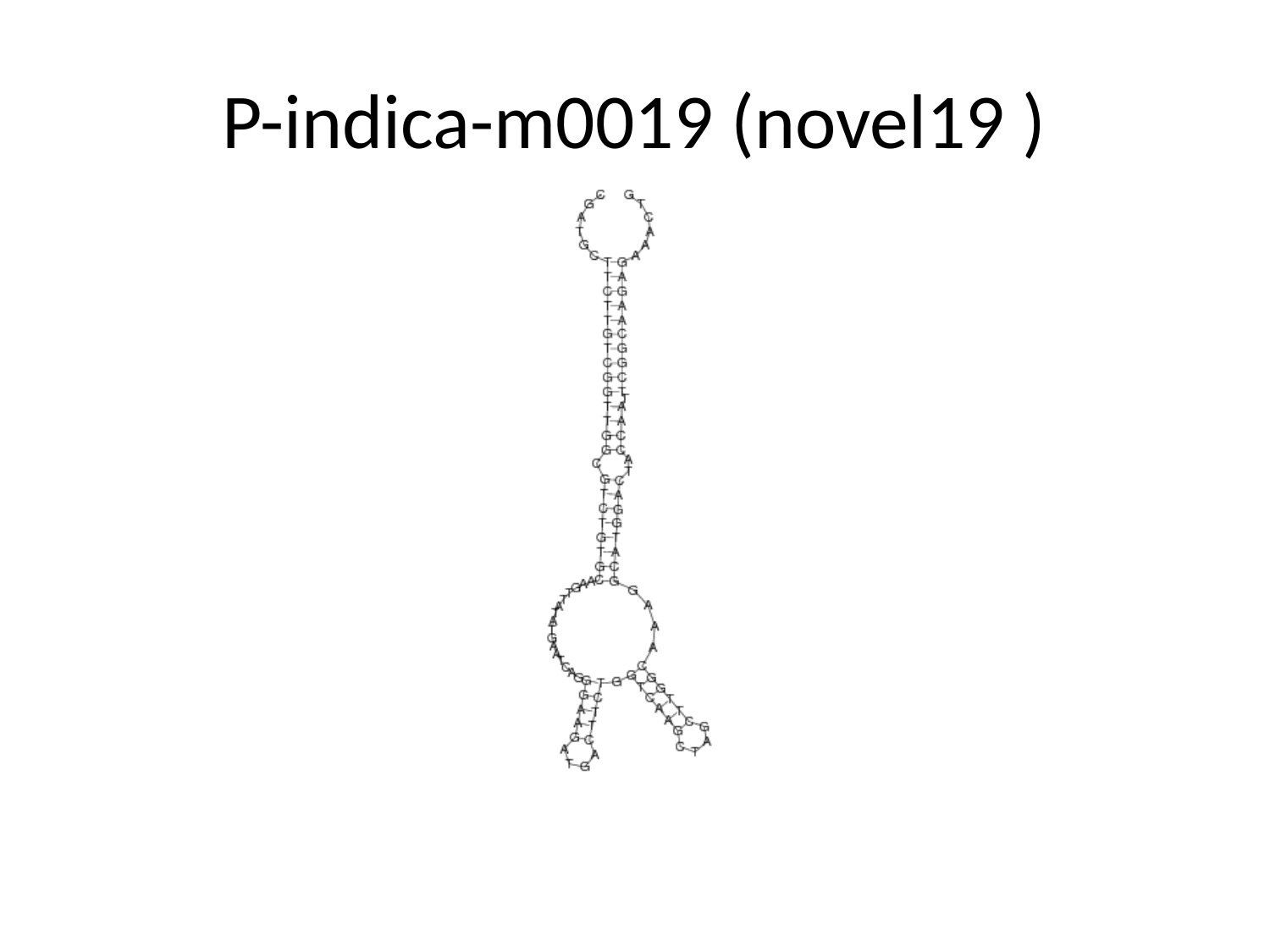

# P-indica-m0019 (novel19 )

## Slide 21
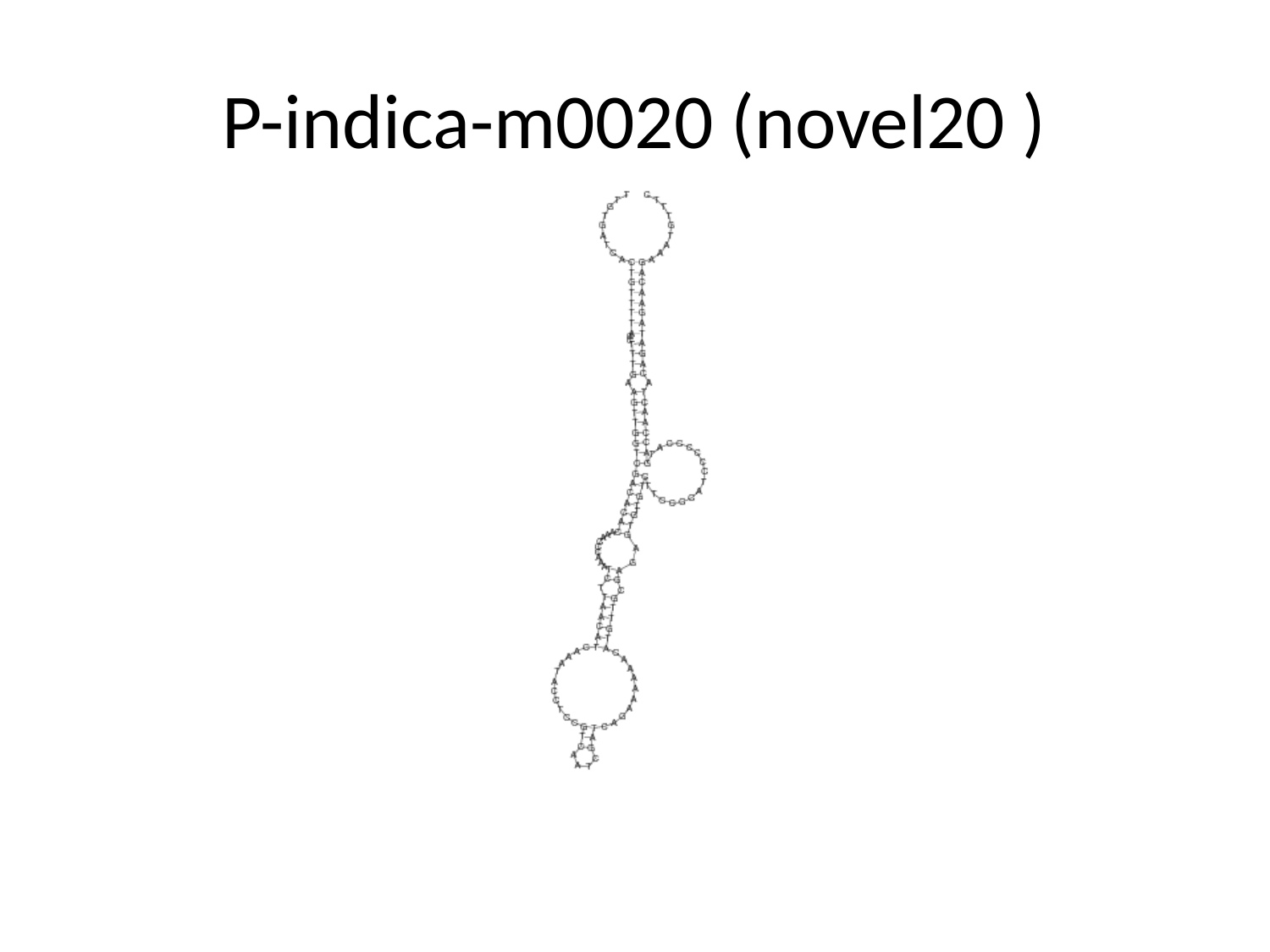

# P-indica-m0020 (novel20 )

## Slide 22
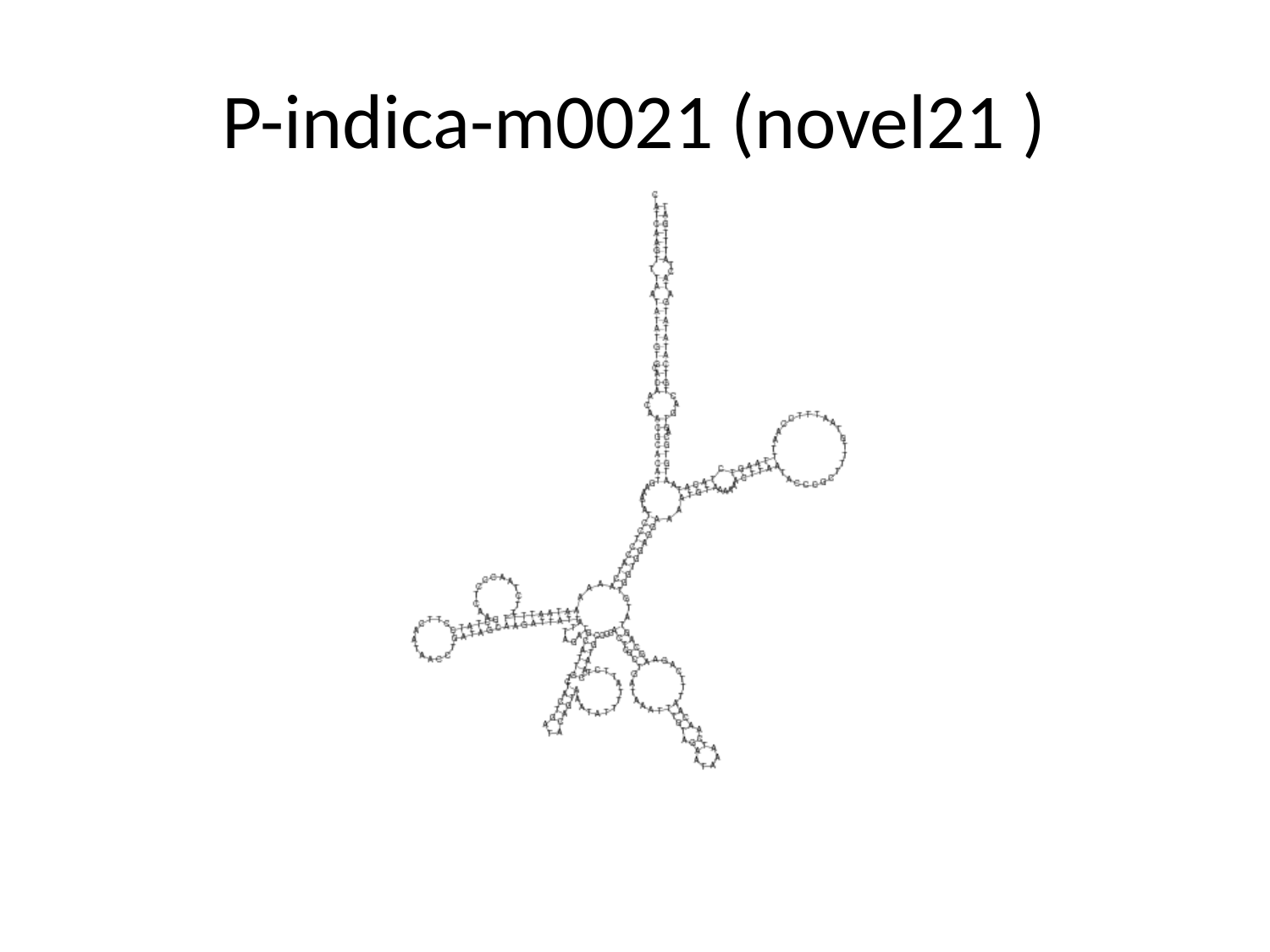

# P-indica-m0021 (novel21 )

## Slide 23
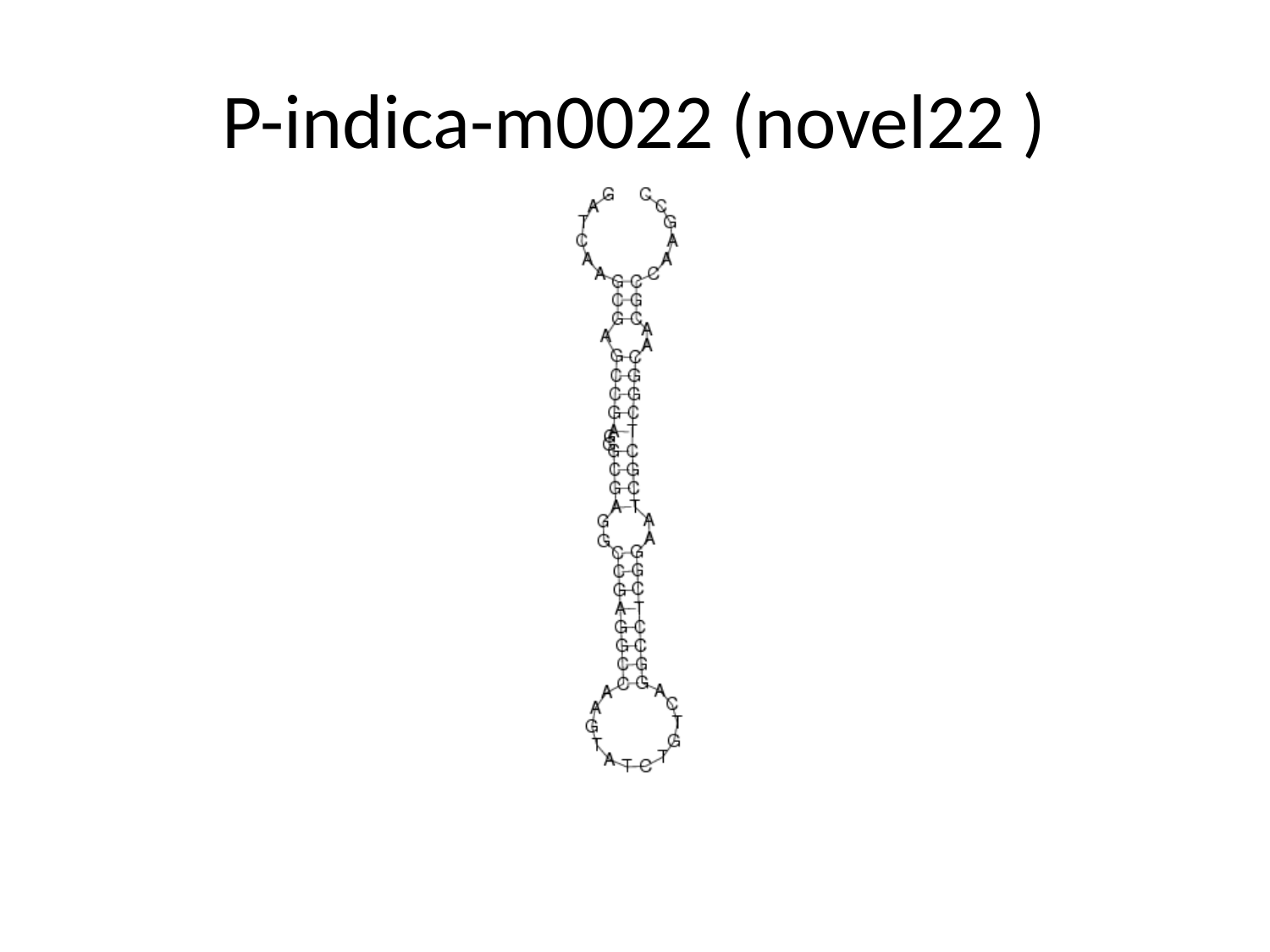

# P-indica-m0022 (novel22 )

## Slide 24
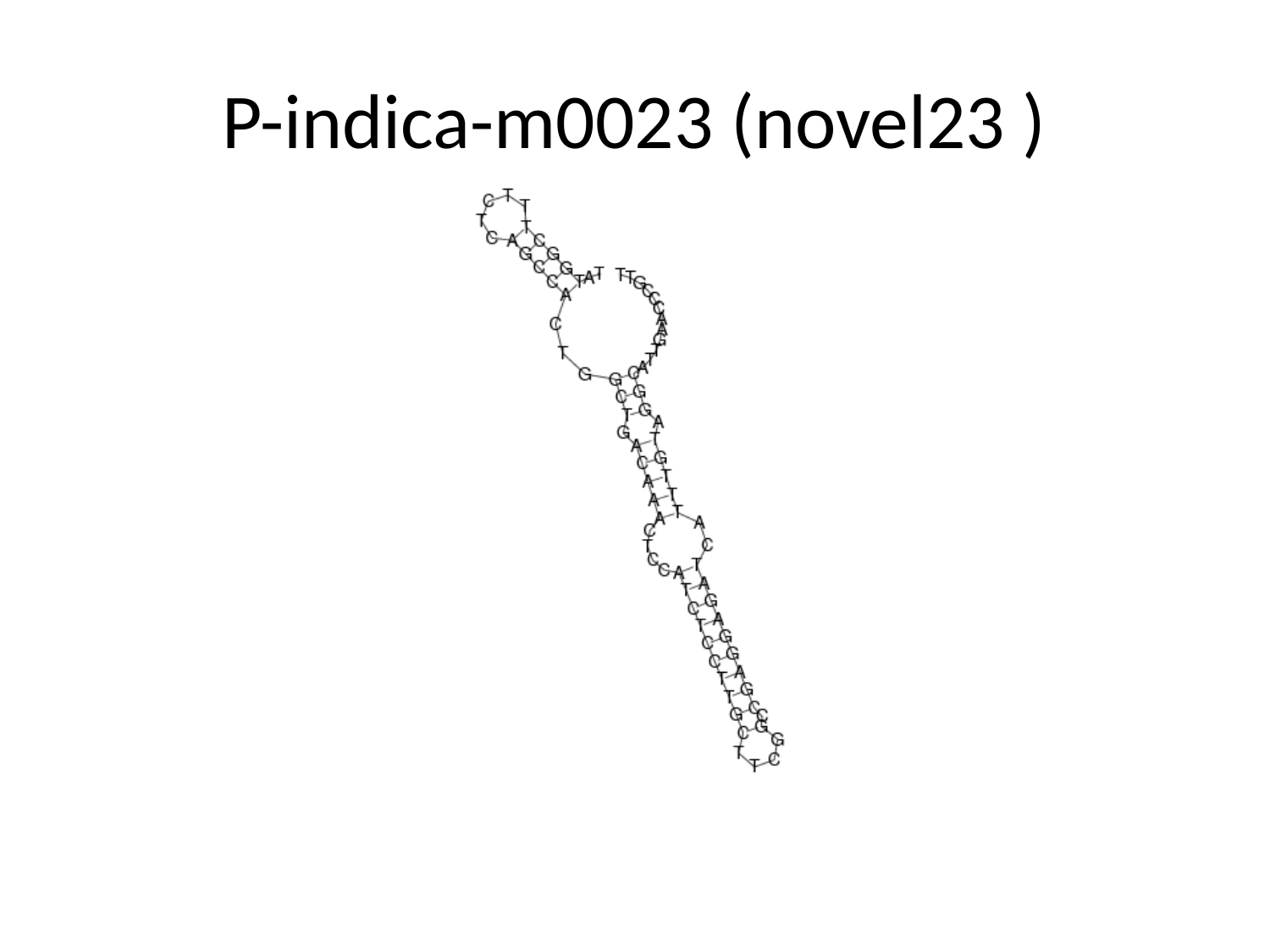

# P-indica-m0023 (novel23 )

## Slide 25
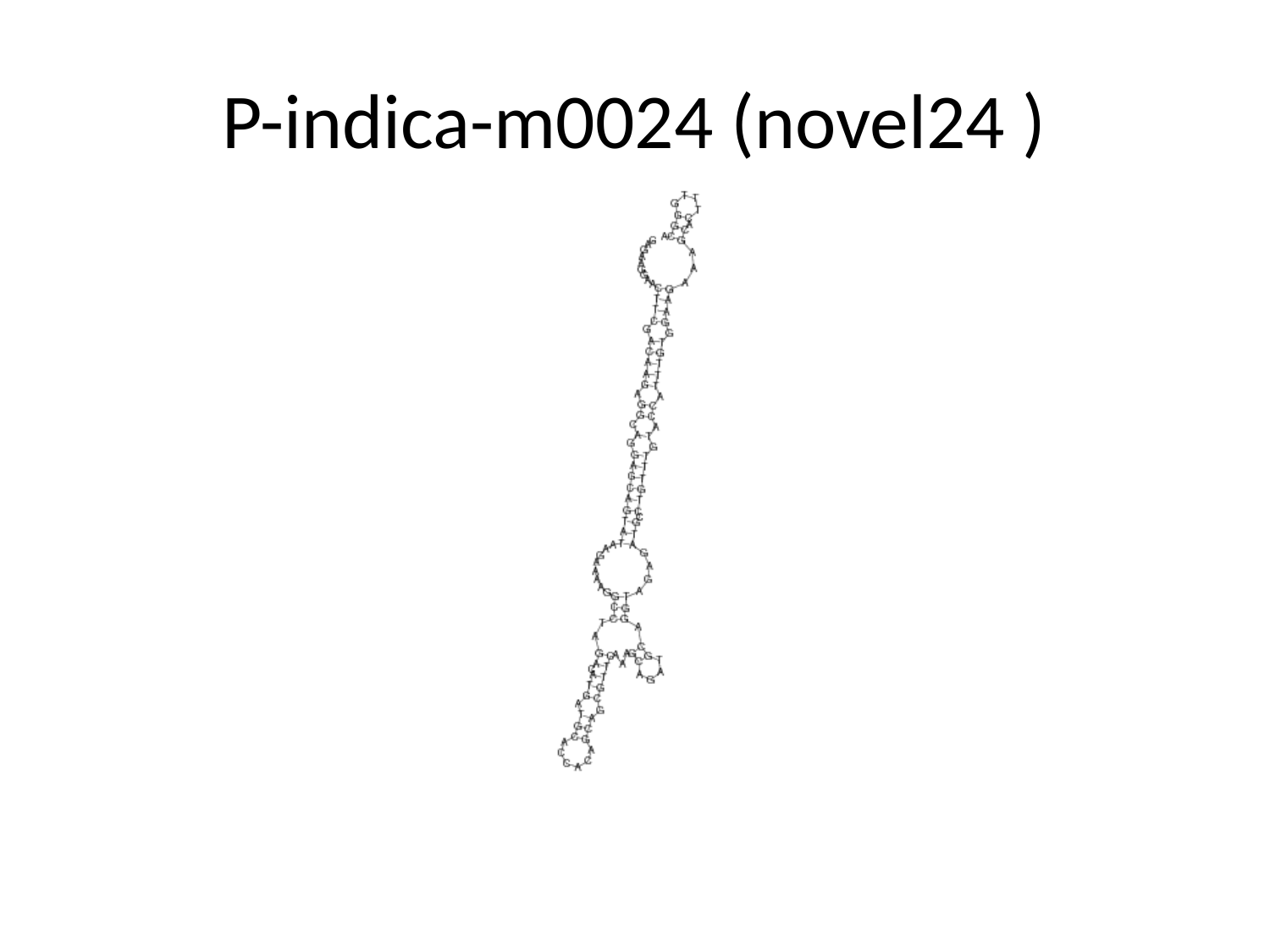

# P-indica-m0024 (novel24 )

## Slide 26
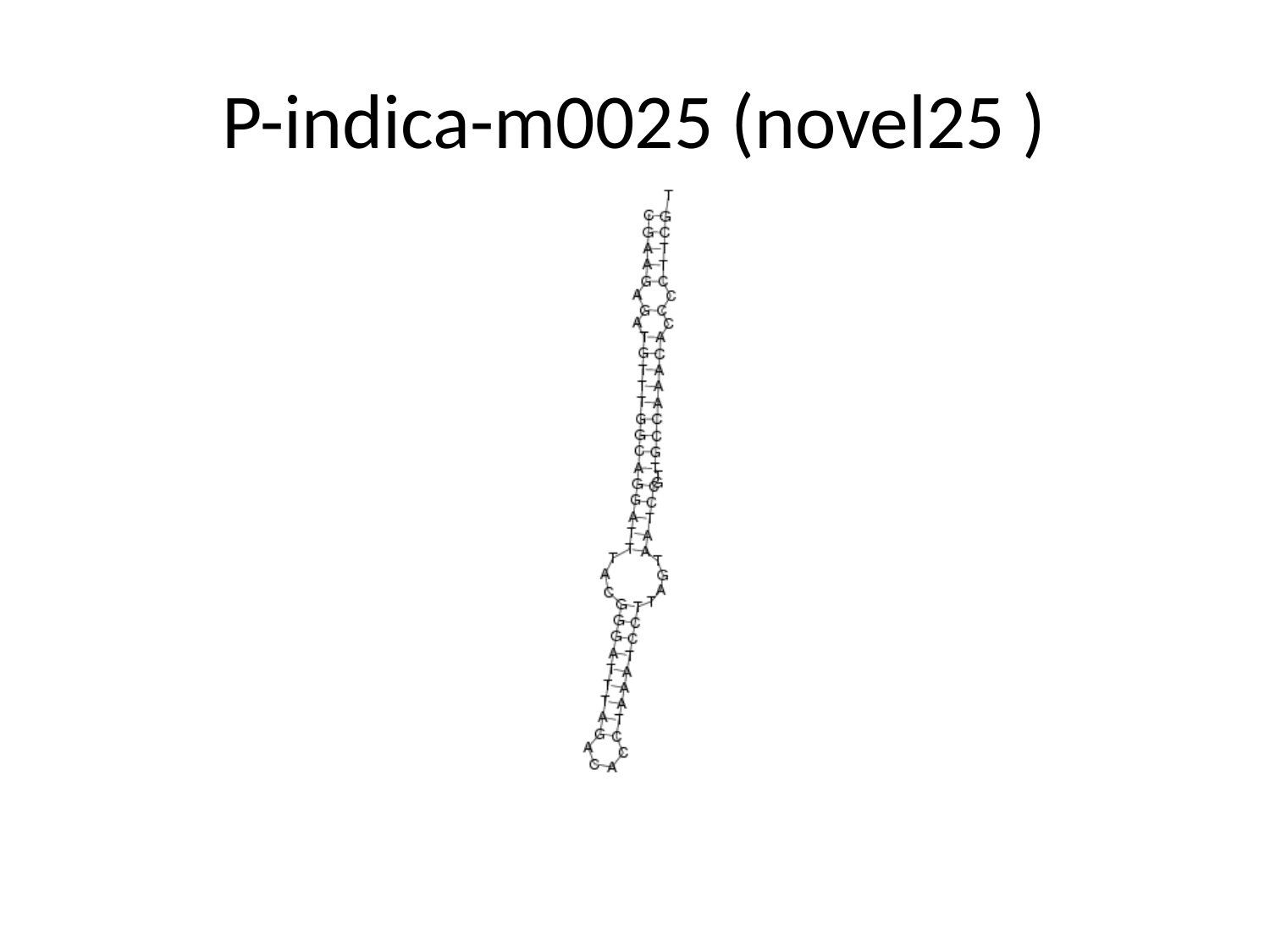

# P-indica-m0025 (novel25 )

## Slide 27
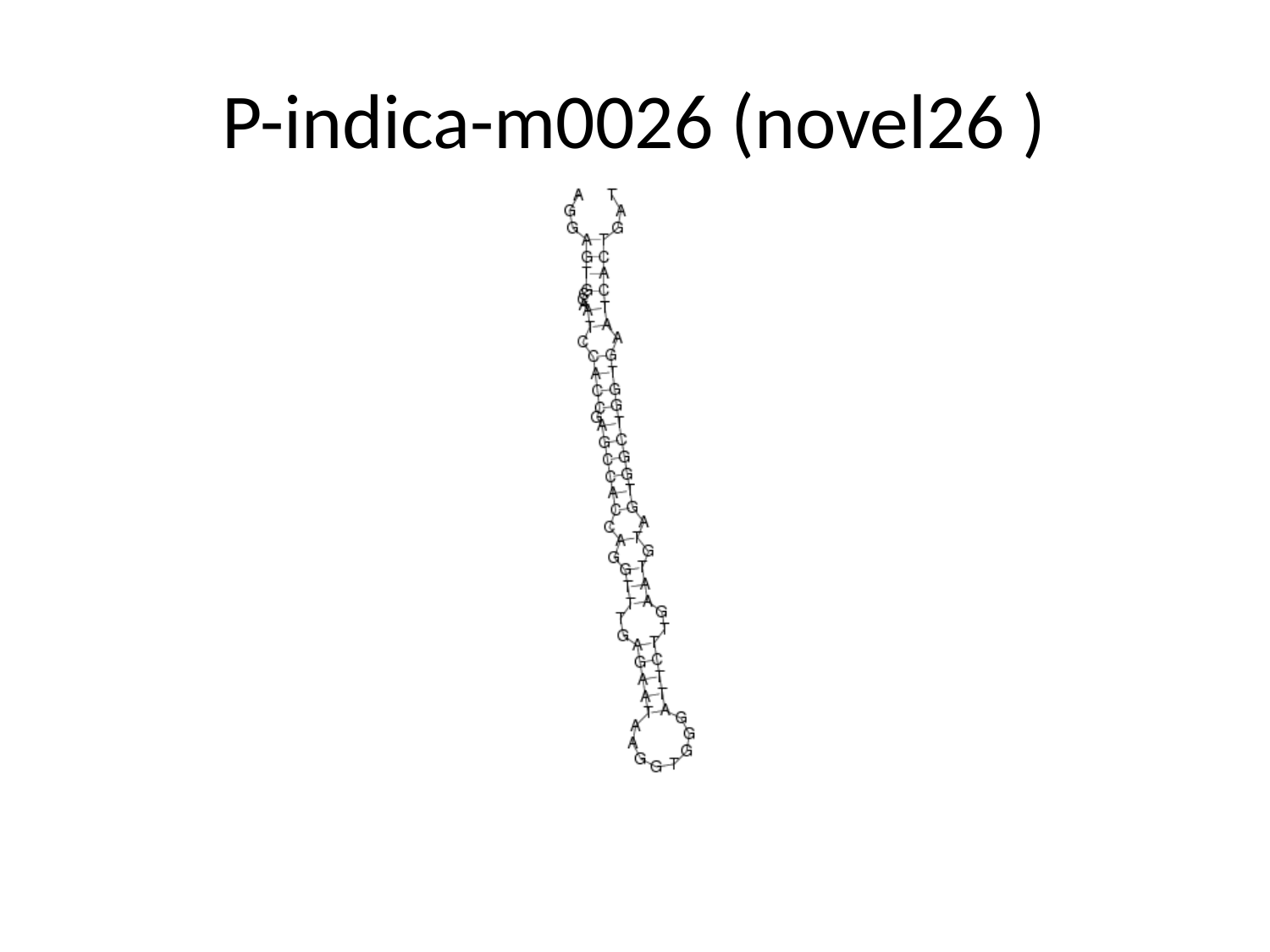

# P-indica-m0026 (novel26 )

## Slide 28
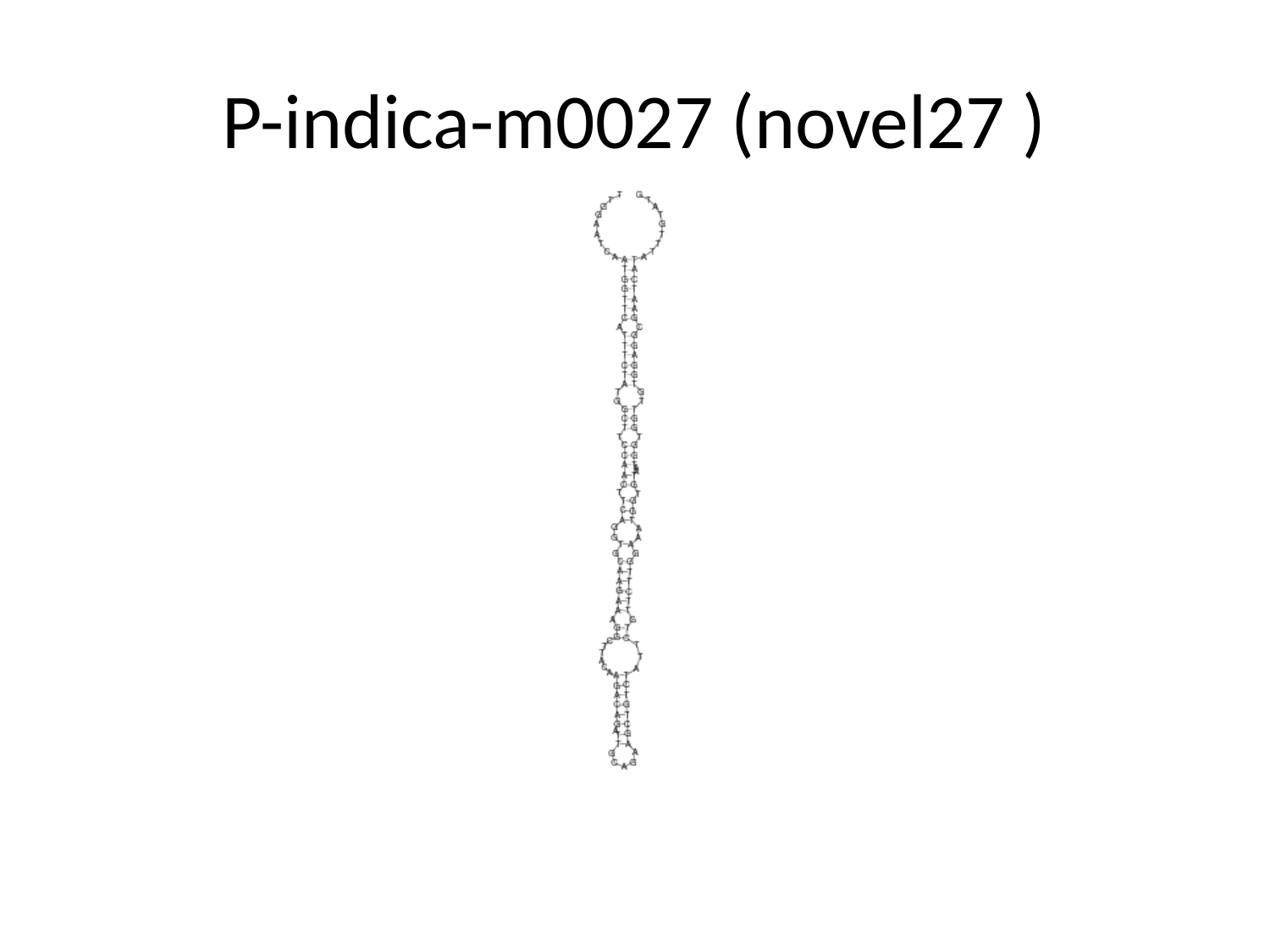

# P-indica-m0027 (novel27 )

## Slide 29
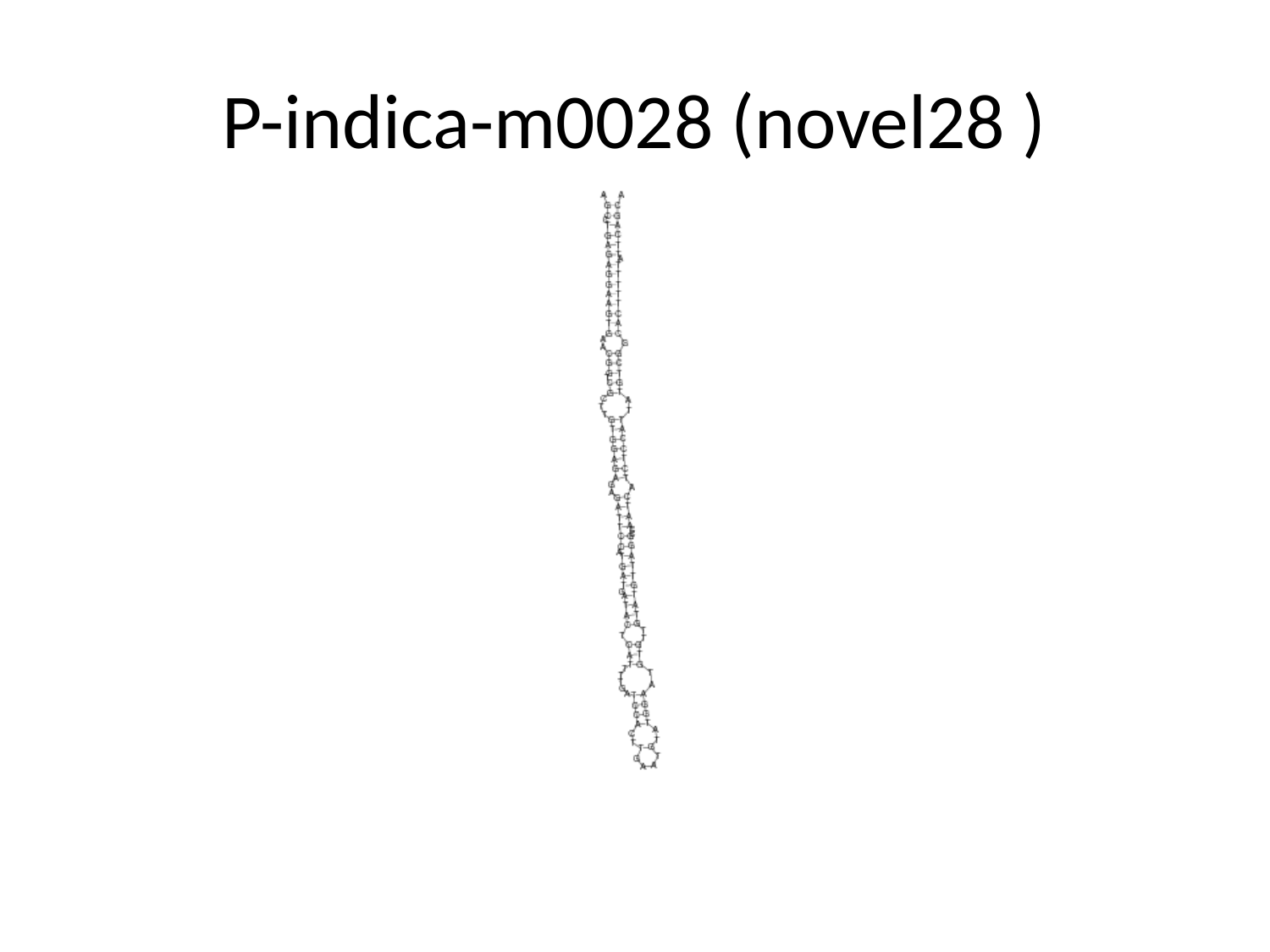

# P-indica-m0028 (novel28 )

## Slide 30
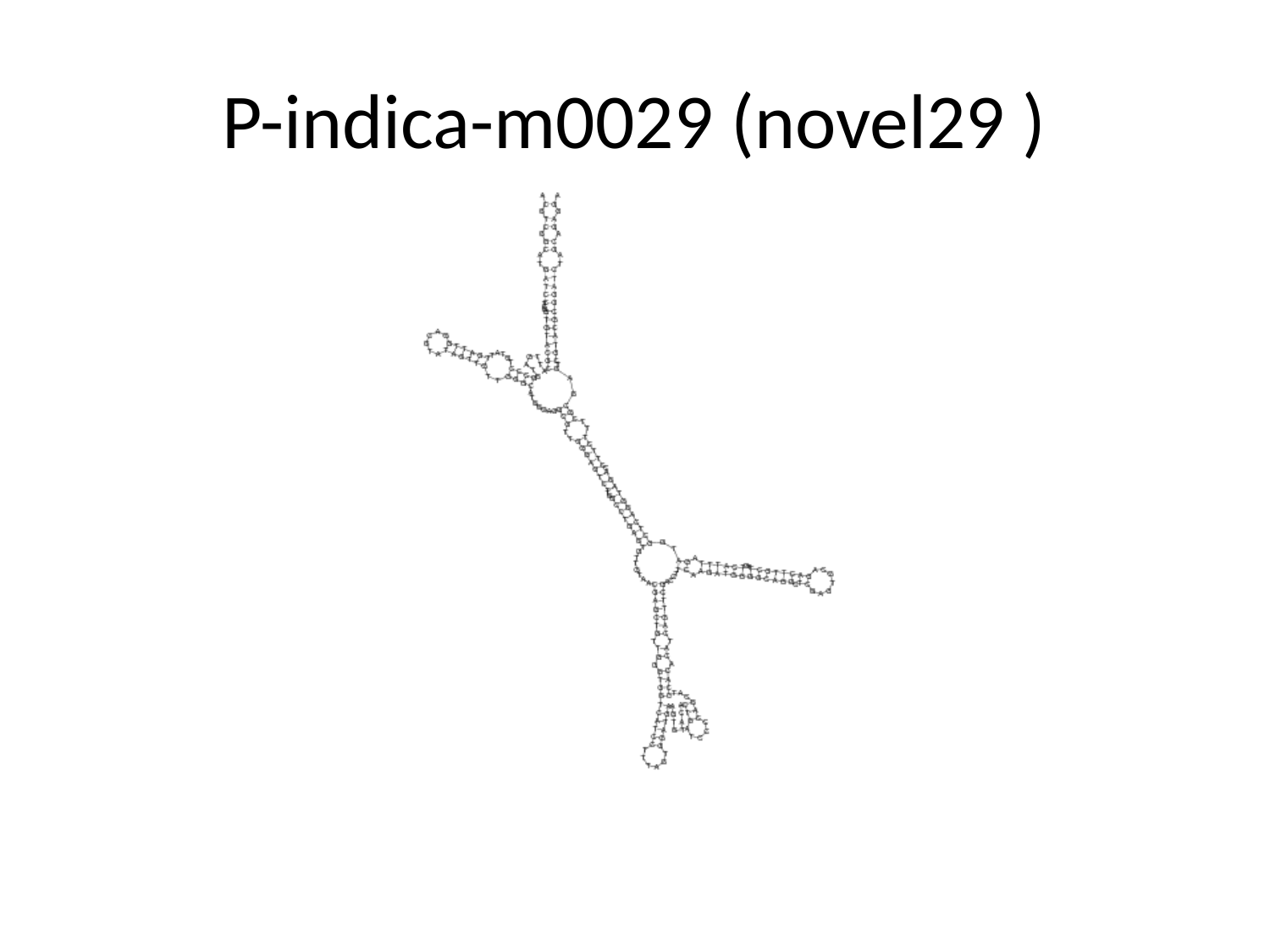

# P-indica-m0029 (novel29 )

## Slide 31
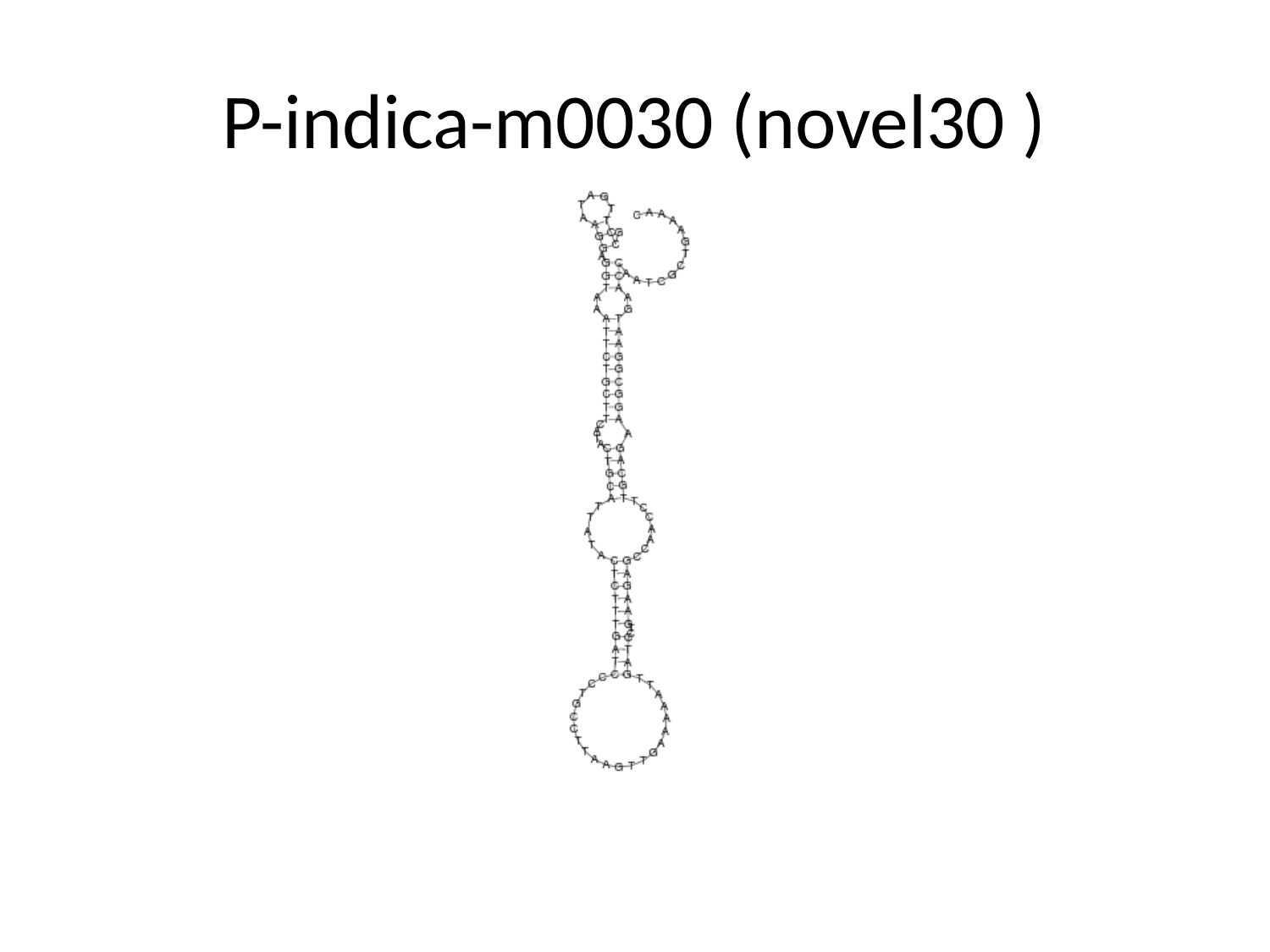

# P-indica-m0030 (novel30 )

## Slide 32
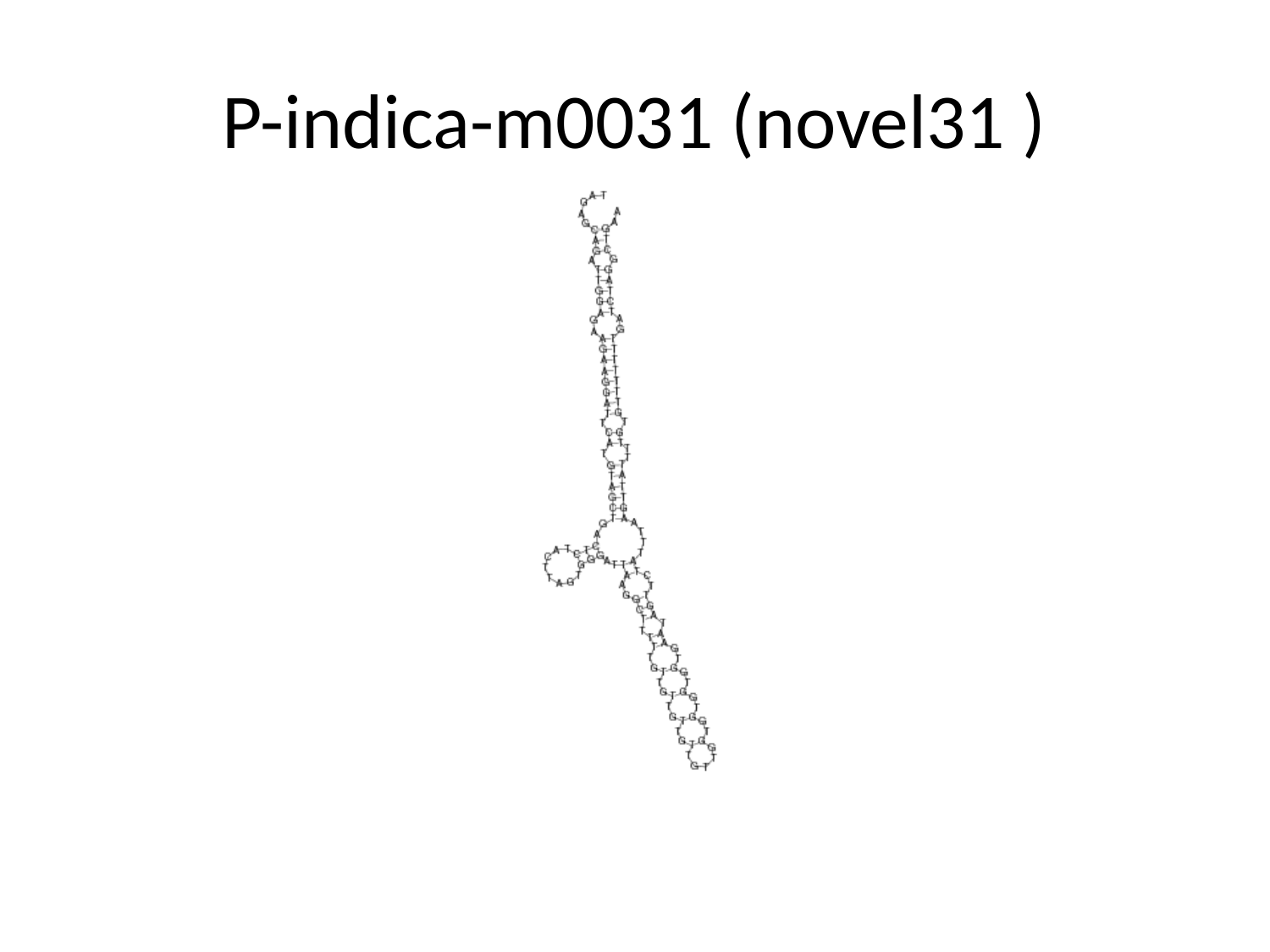

# P-indica-m0031 (novel31 )

## Slide 33
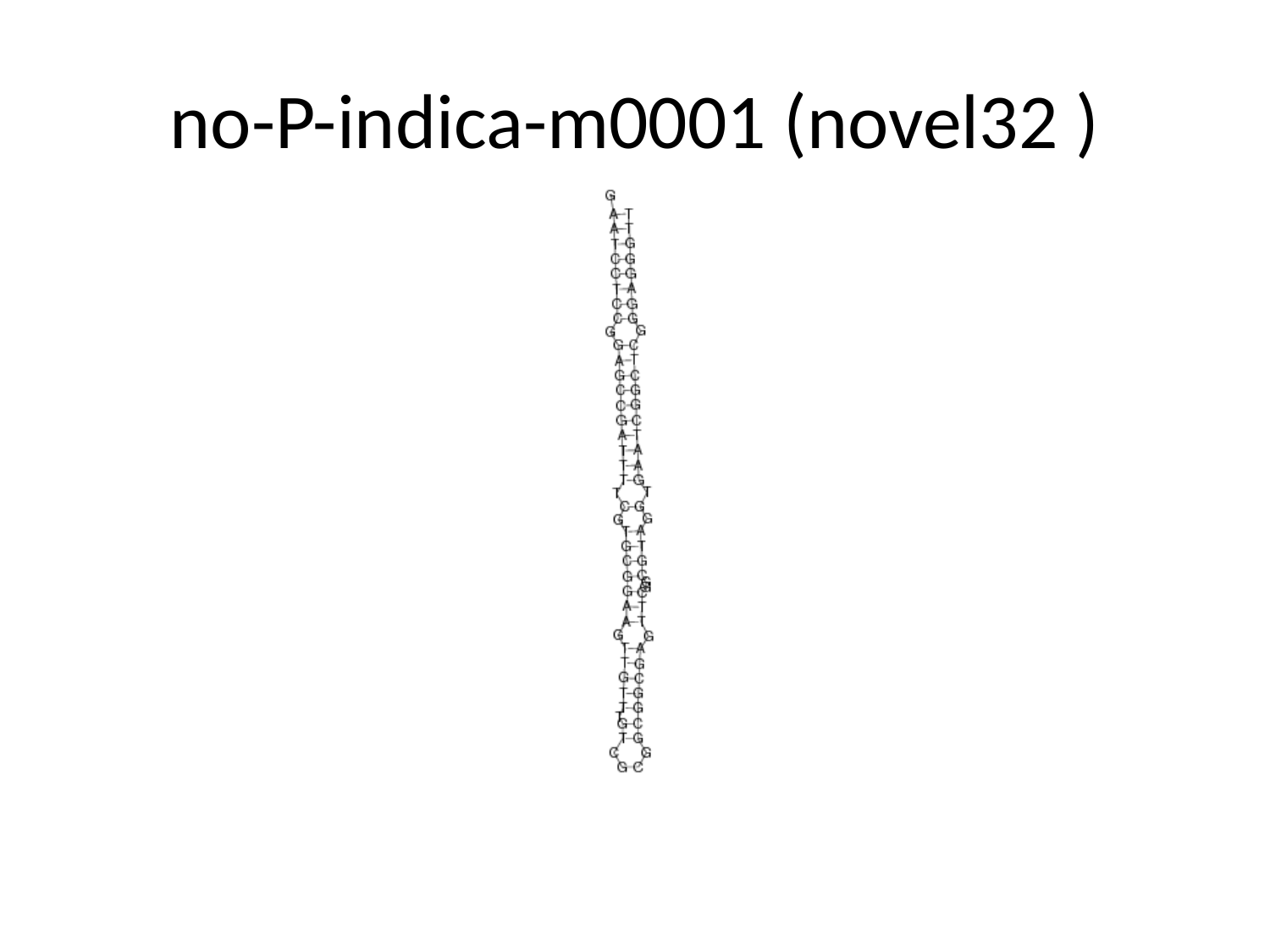

# no-P-indica-m0001 (novel32 )

## Slide 34
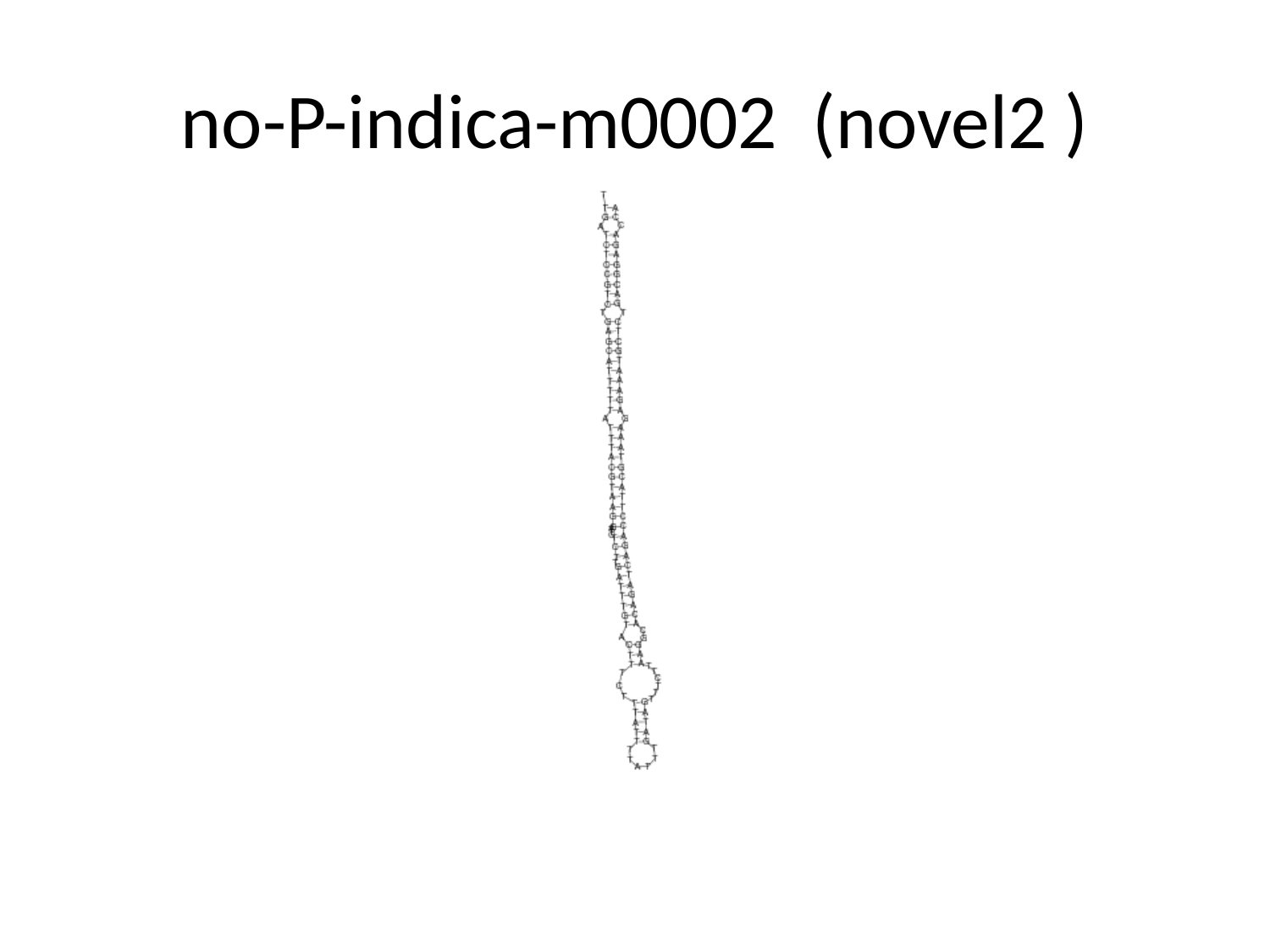

# no-P-indica-m0002 (novel2 )

## Slide 35
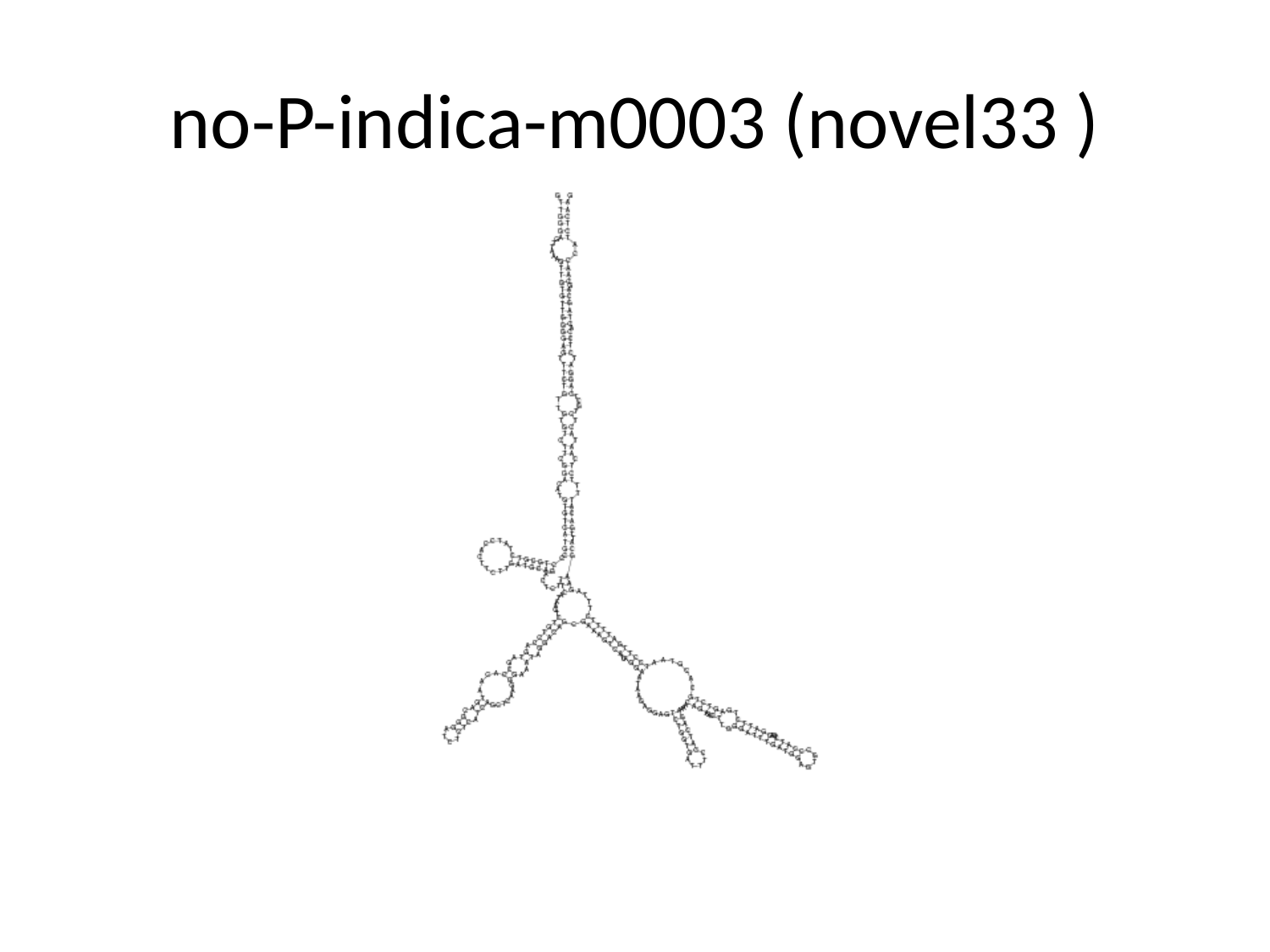

# no-P-indica-m0003 (novel33 )

## Slide 36
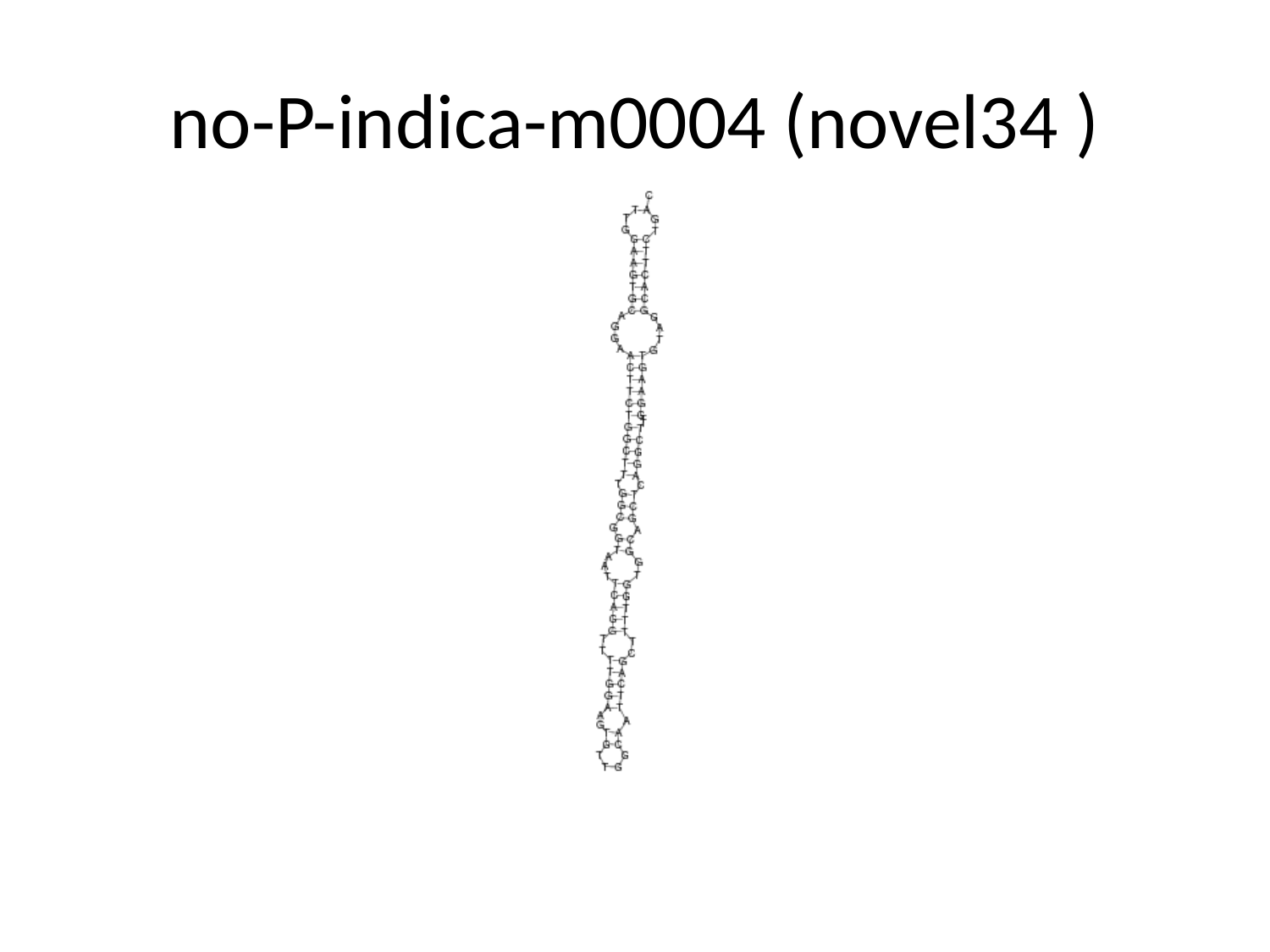

# no-P-indica-m0004 (novel34 )

## Slide 37
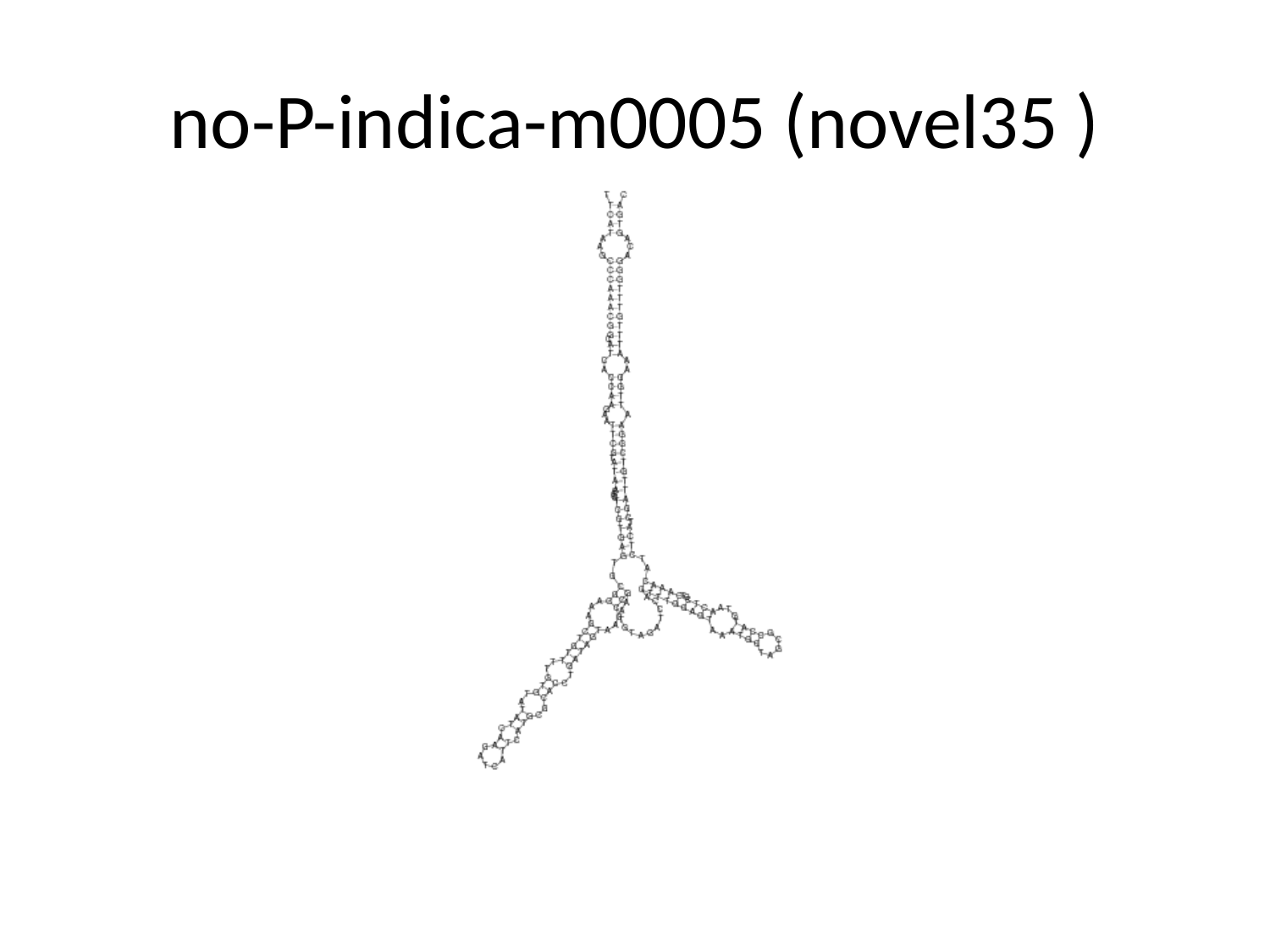

# no-P-indica-m0005 (novel35 )

## Slide 38
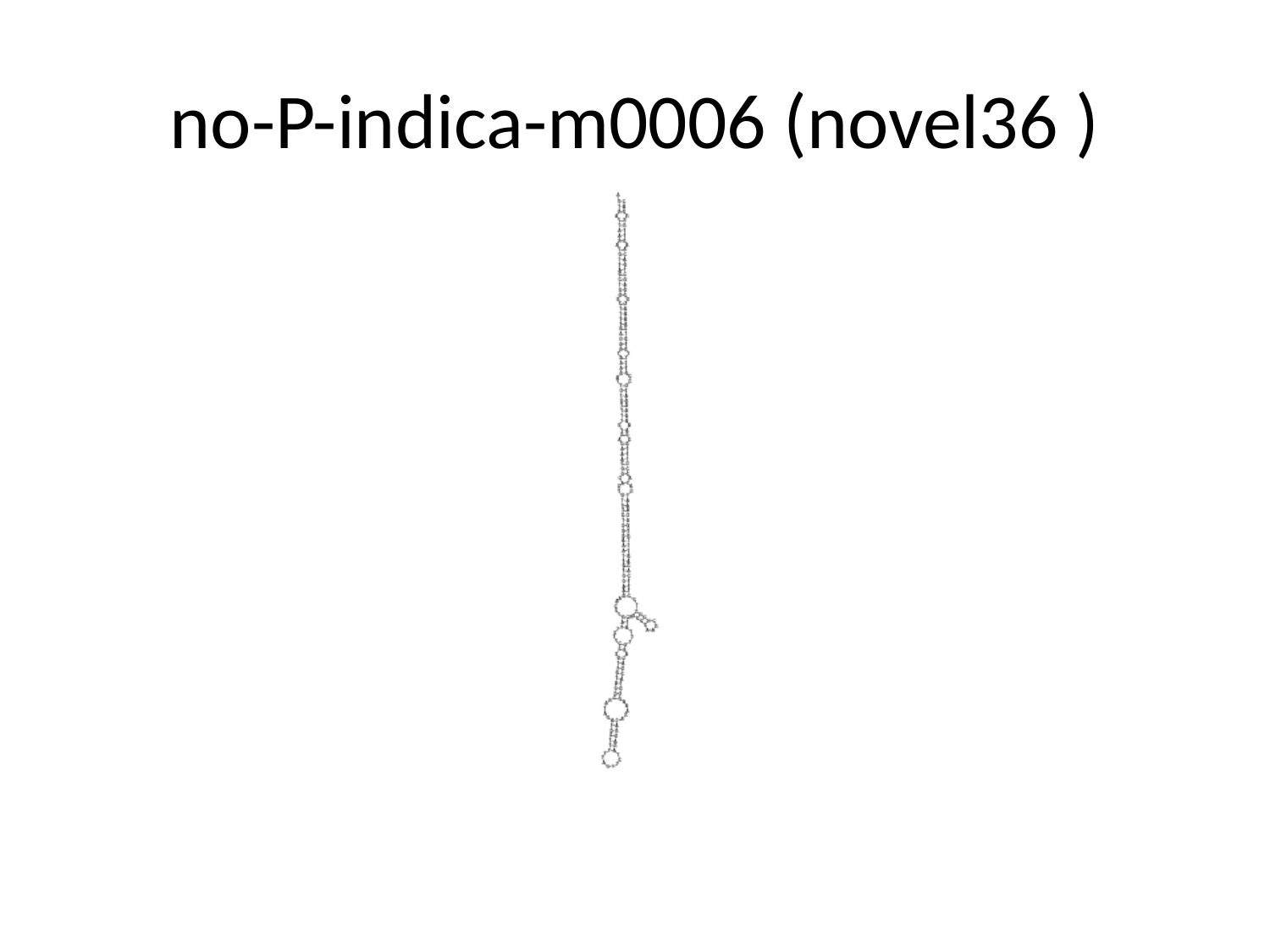

# no-P-indica-m0006 (novel36 )

## Slide 39
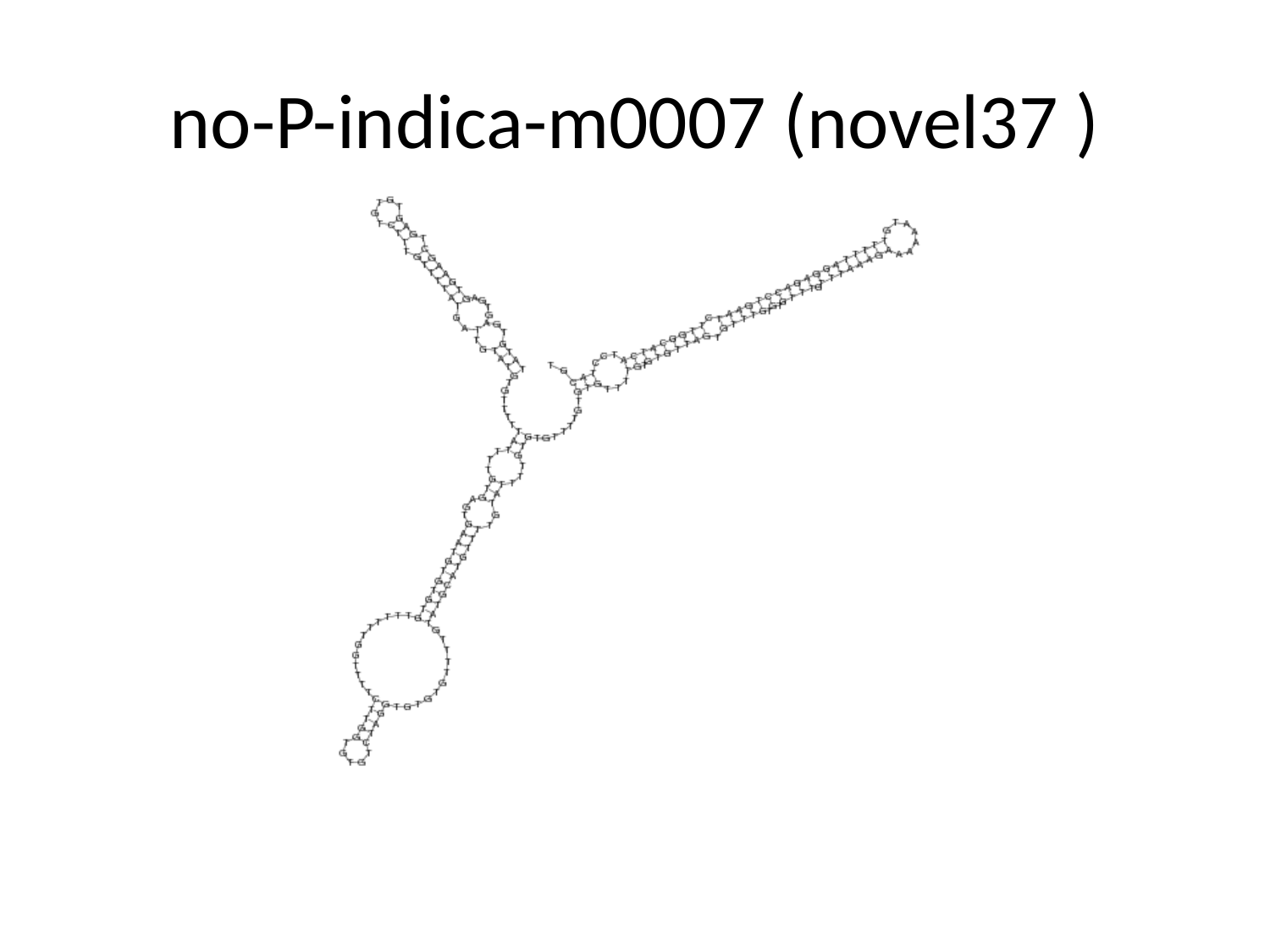

# no-P-indica-m0007 (novel37 )

## Slide 40
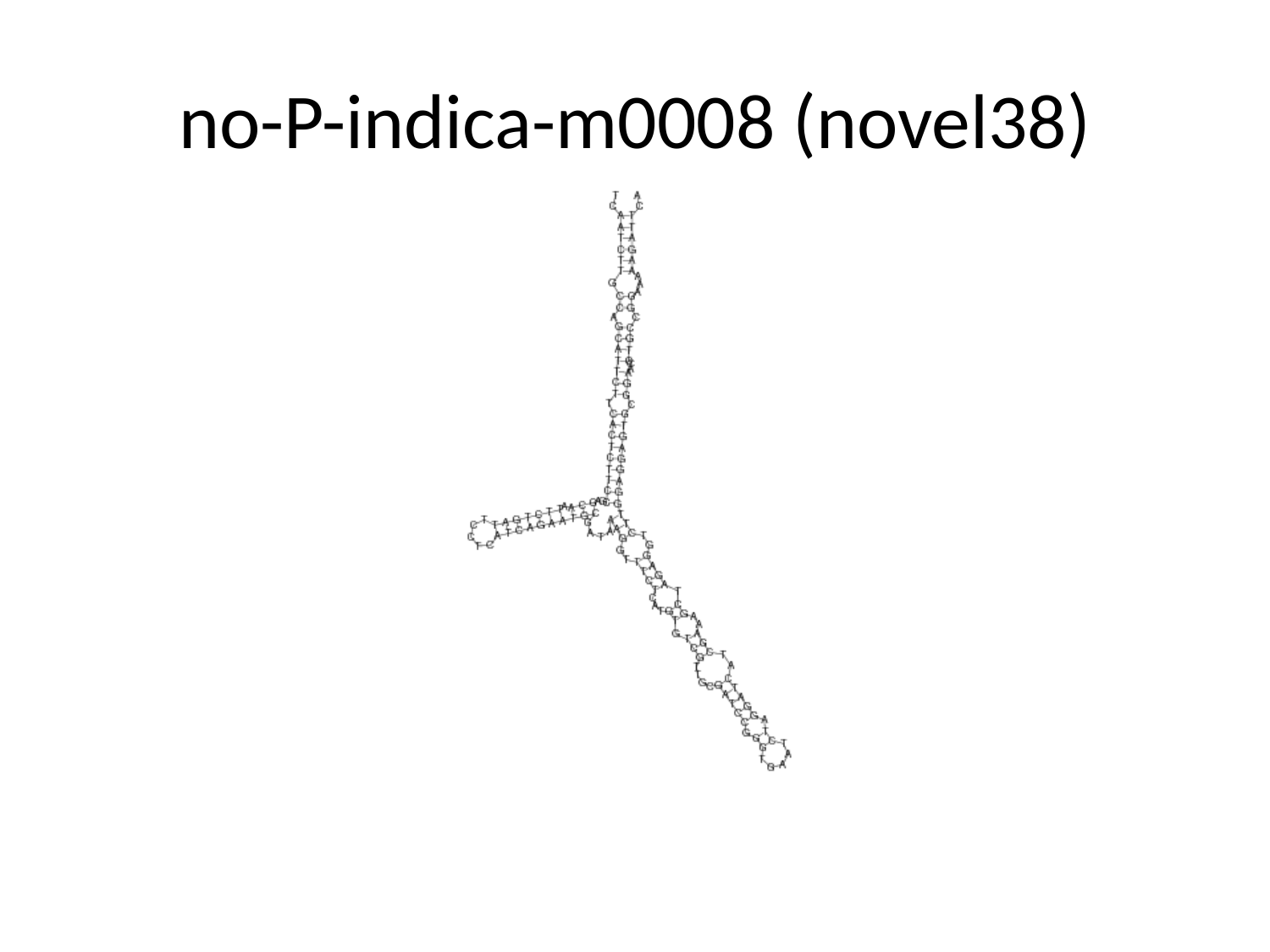

# no-P-indica-m0008 (novel38)

## Slide 41
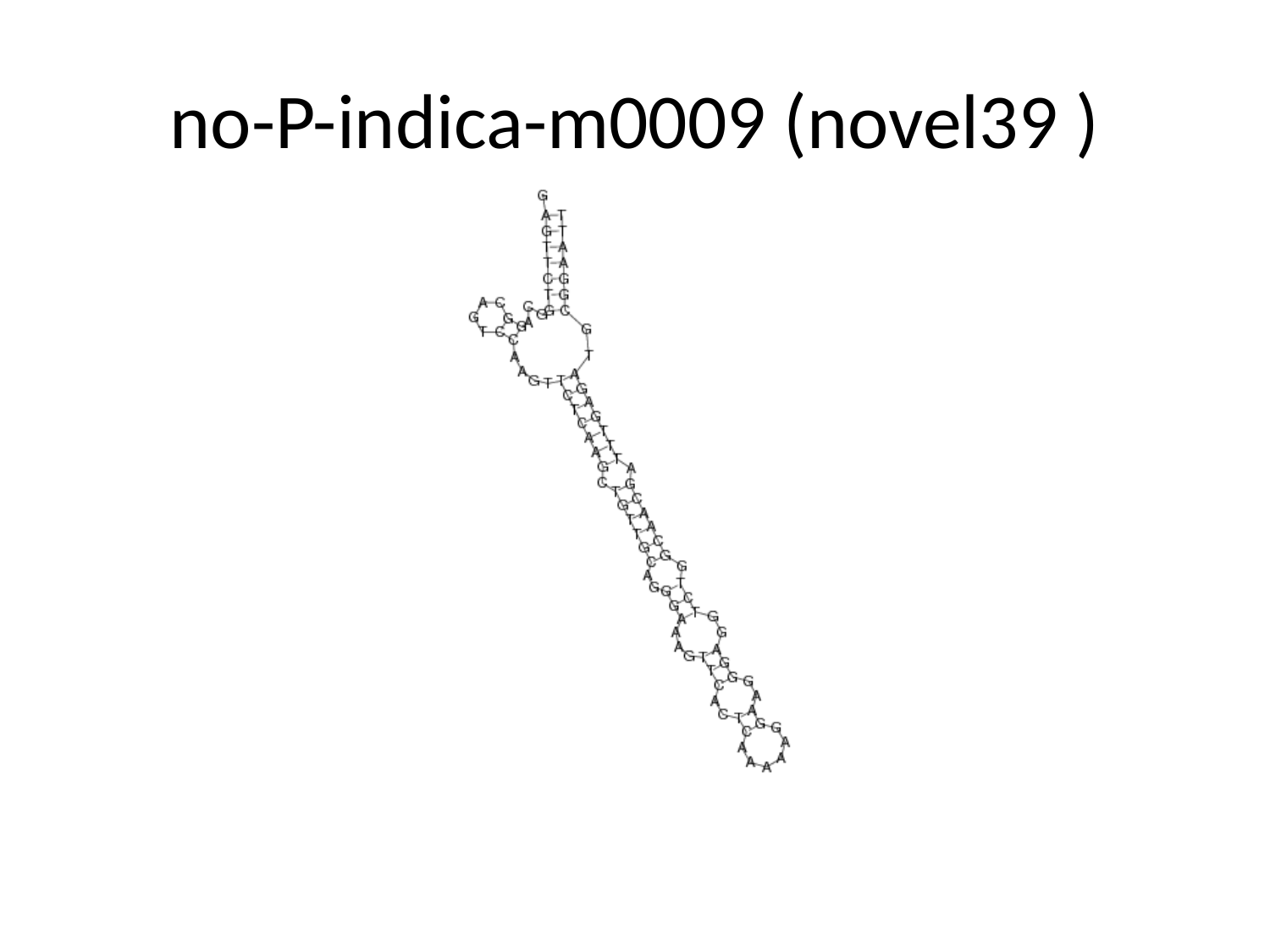

# no-P-indica-m0009 (novel39 )

## Slide 42
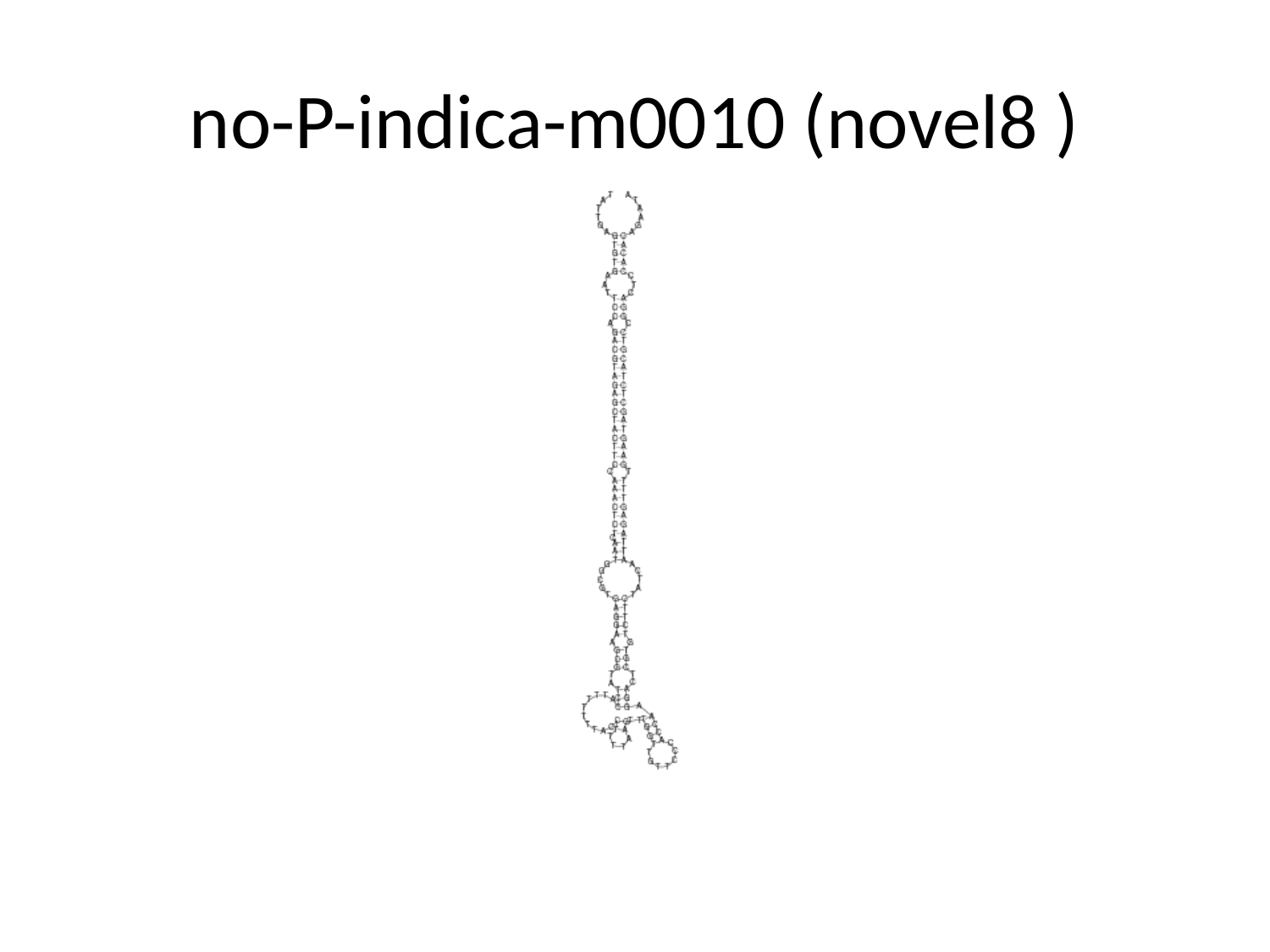

# no-P-indica-m0010 (novel8 )

## Slide 43
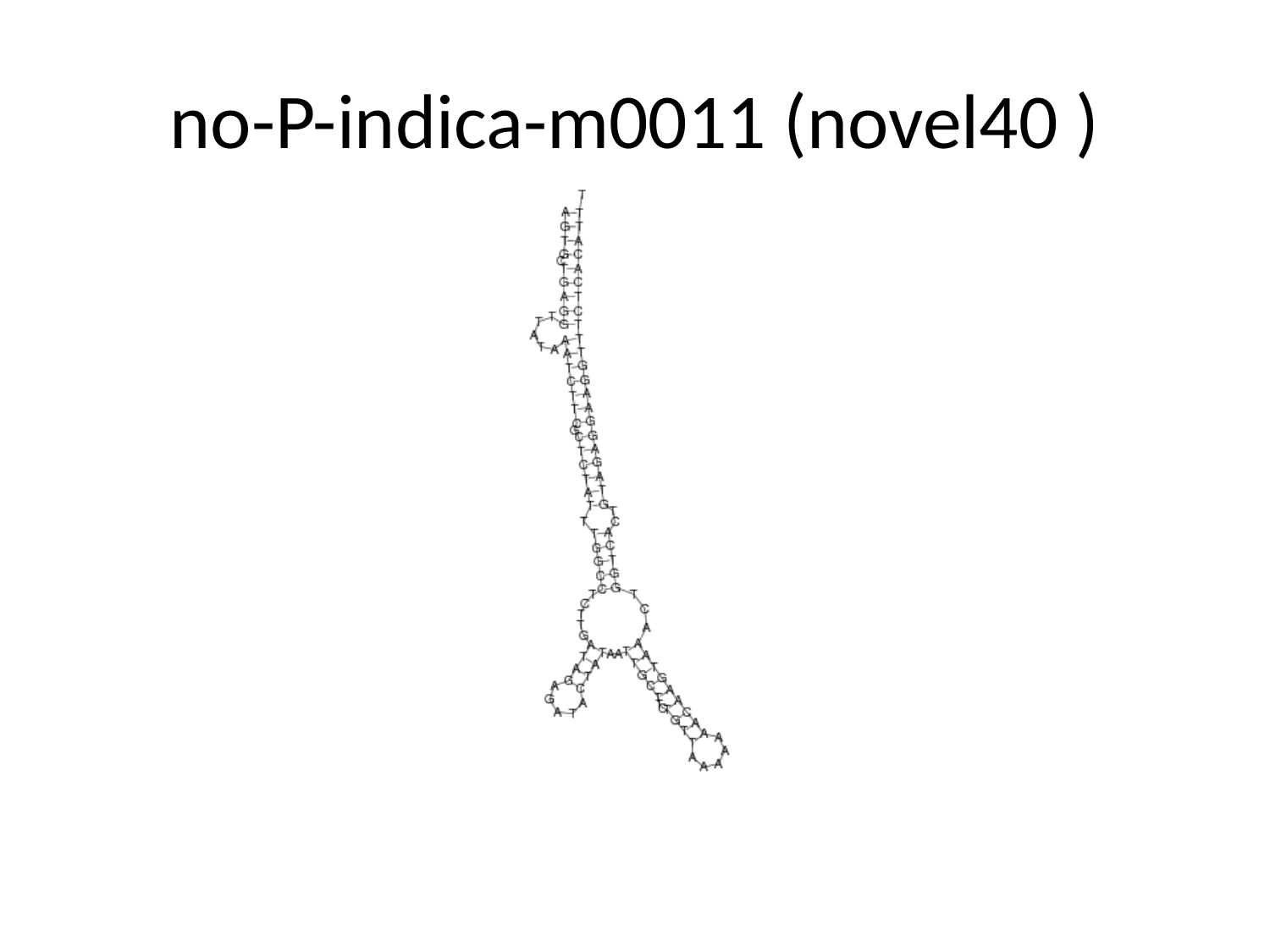

# no-P-indica-m0011 (novel40 )

## Slide 44
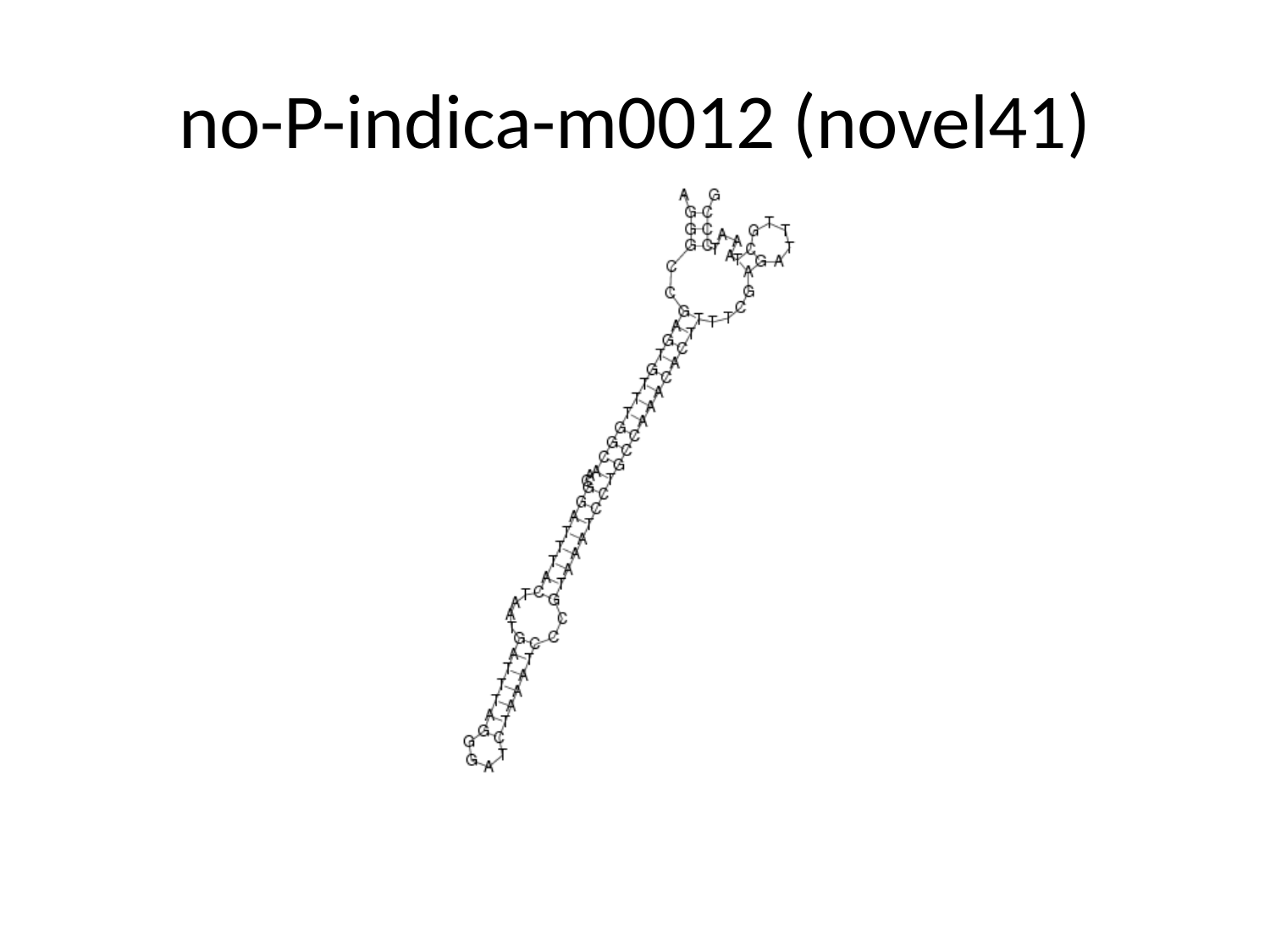

# no-P-indica-m0012 (novel41)

## Slide 45
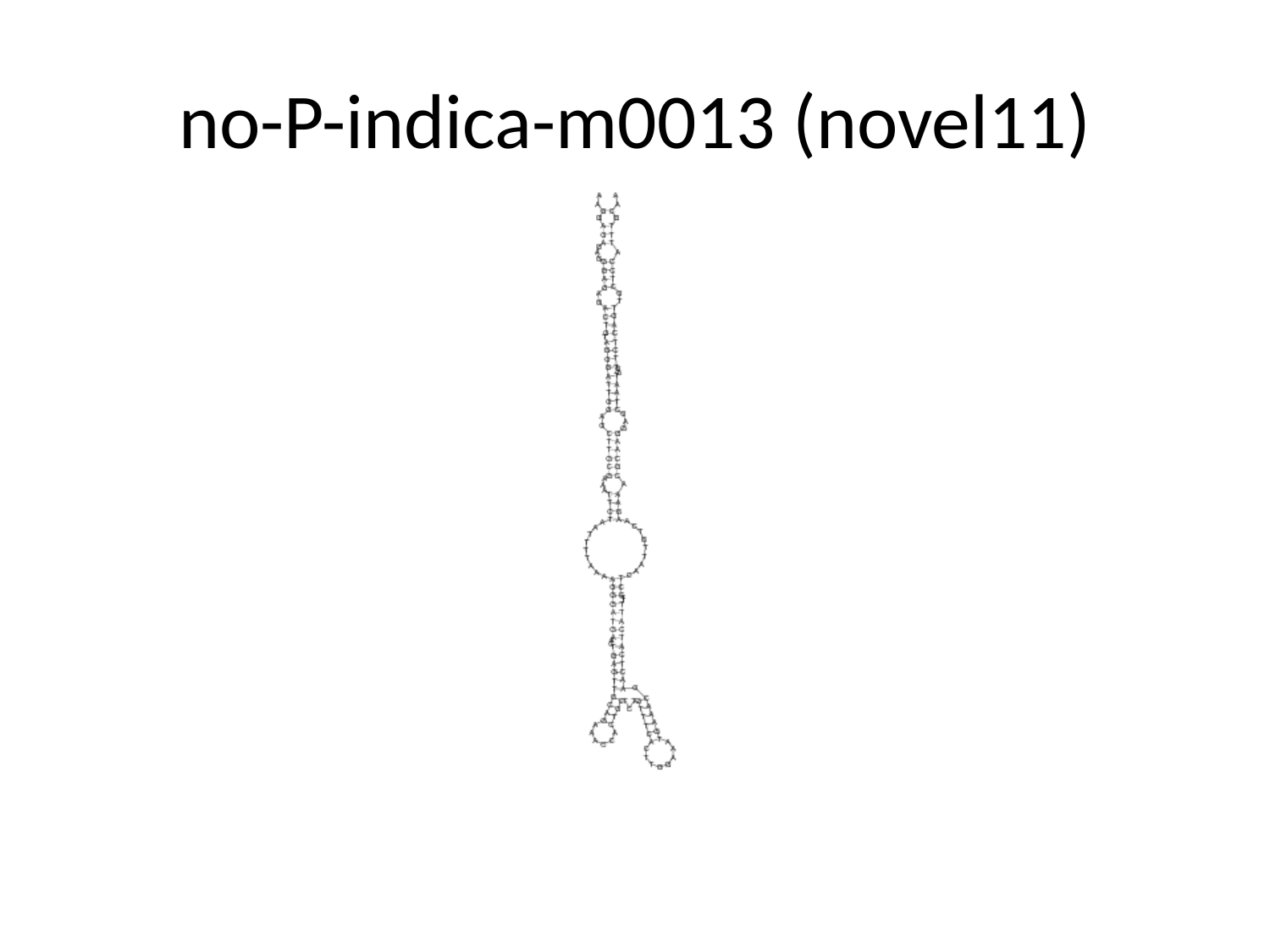

# no-P-indica-m0013 (novel11)

## Slide 46
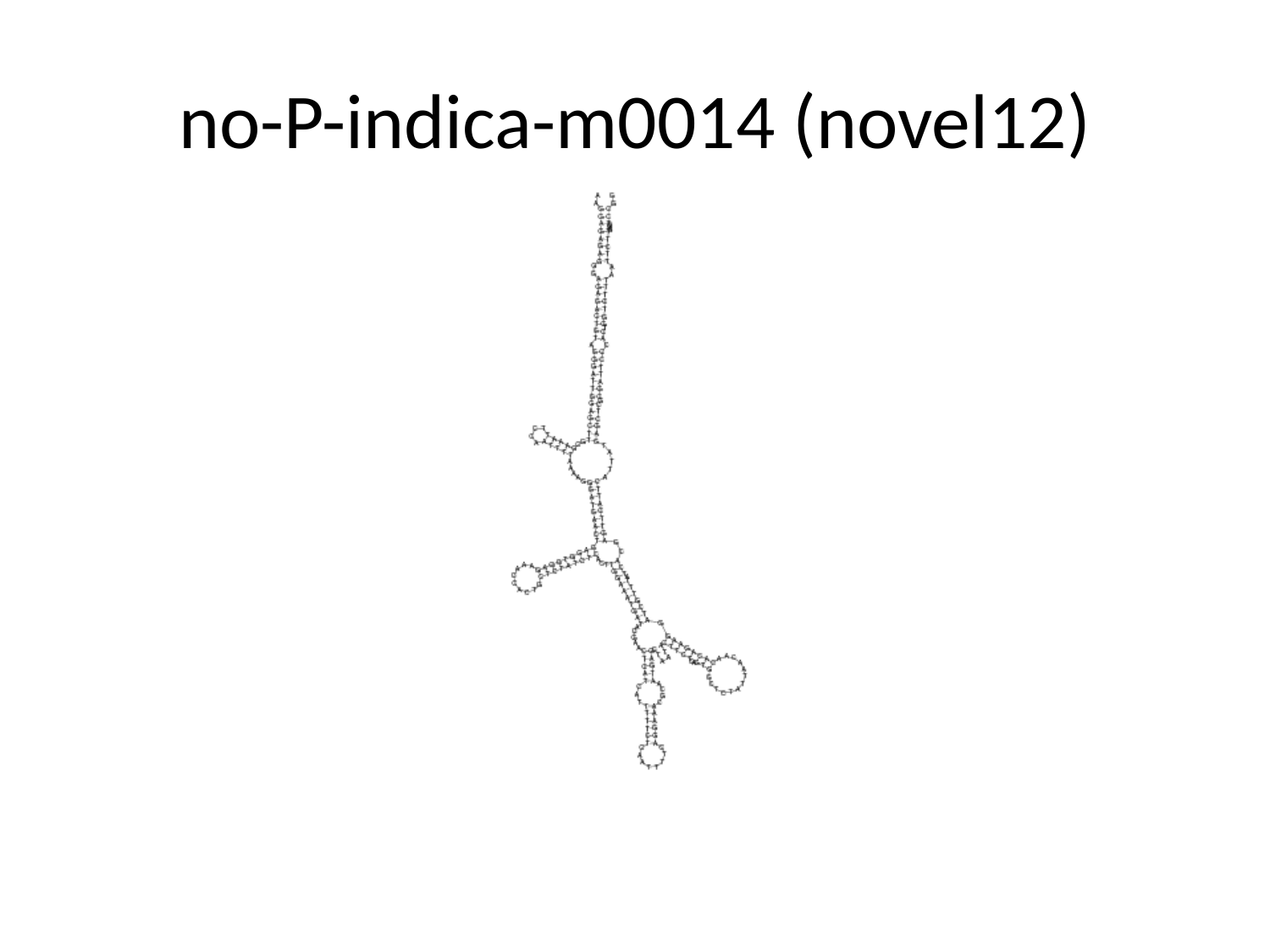

# no-P-indica-m0014 (novel12)

## Slide 47
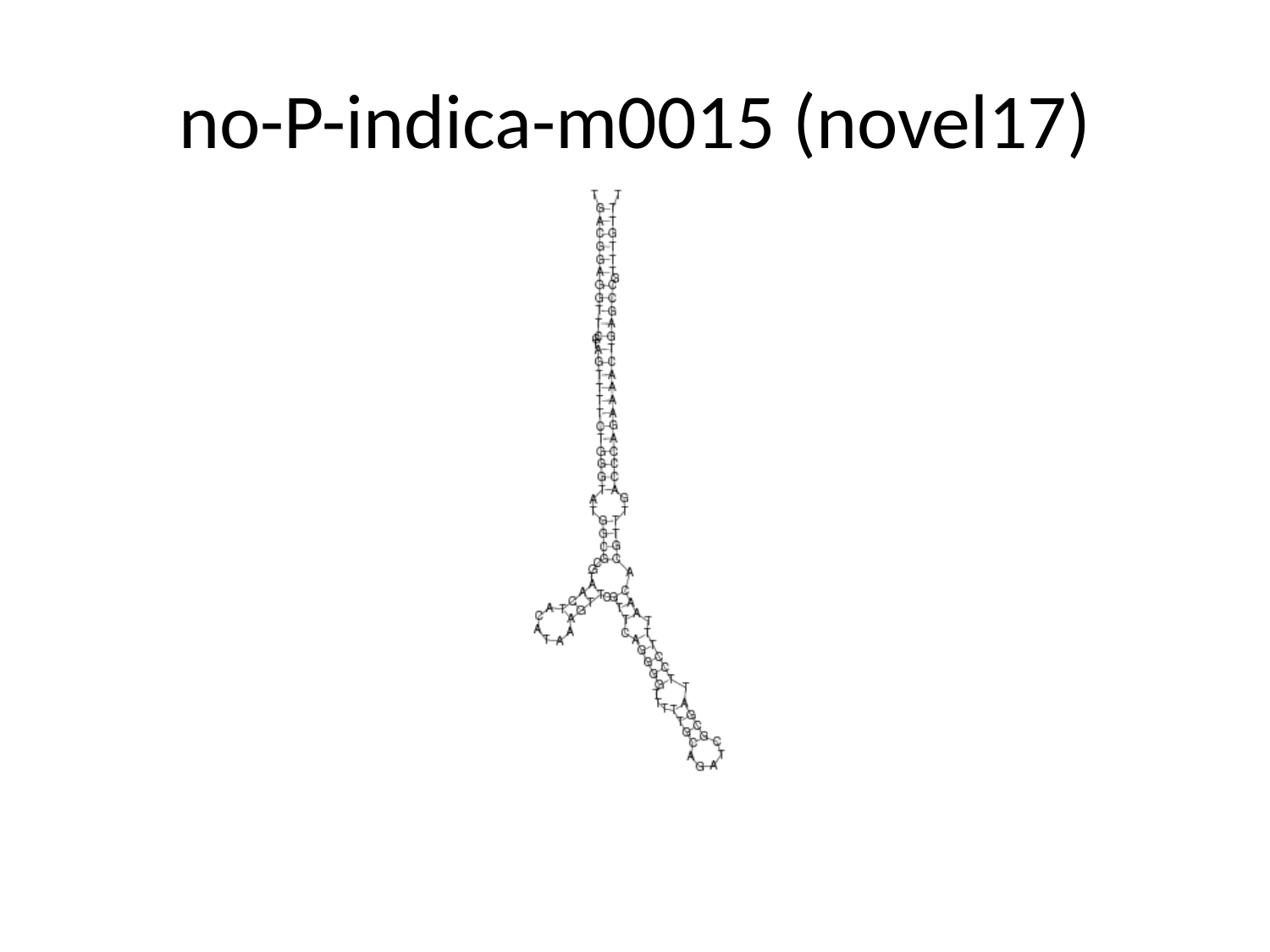

# no-P-indica-m0015 (novel17)

## Slide 48
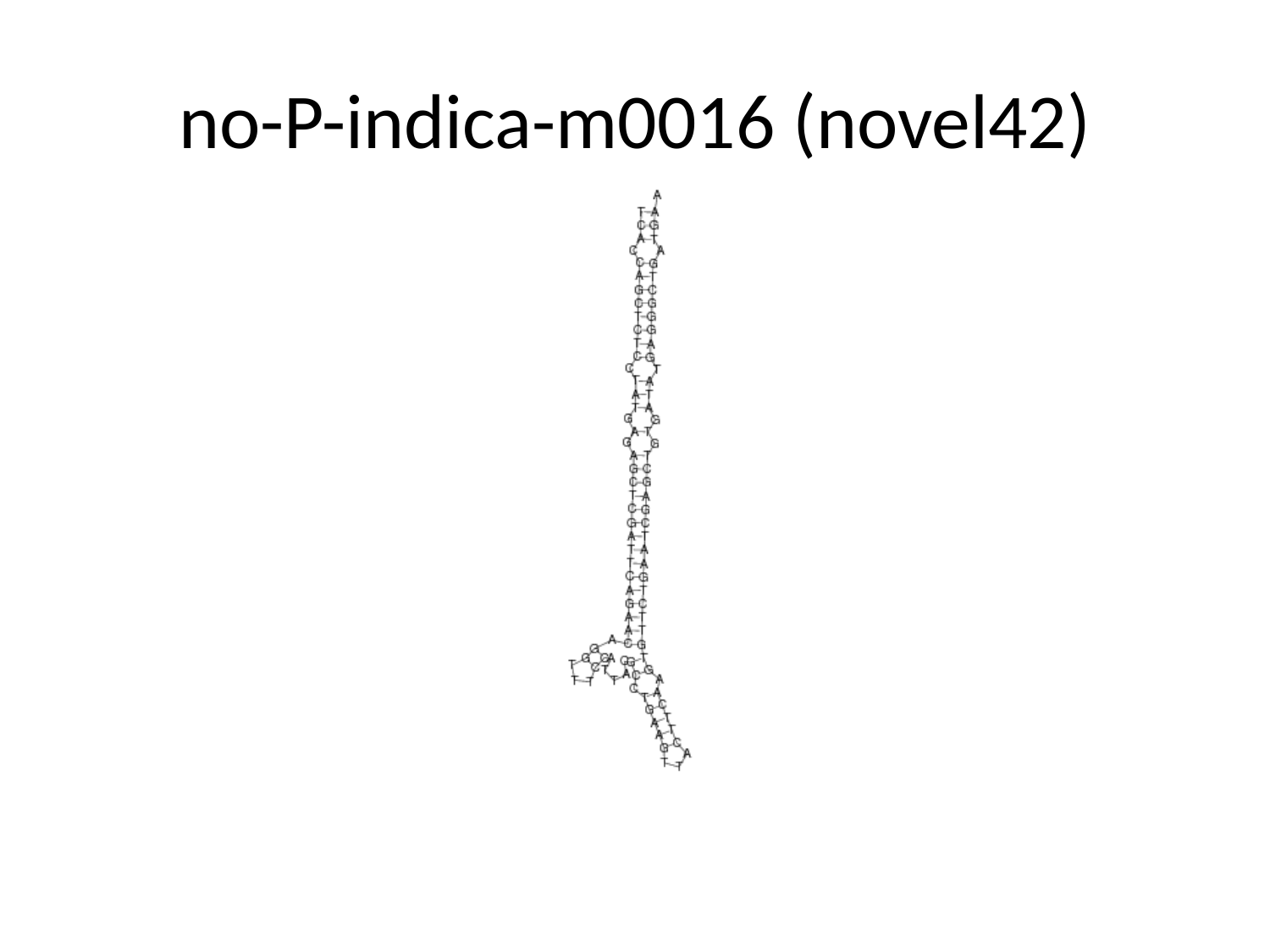

# no-P-indica-m0016 (novel42)

## Slide 49
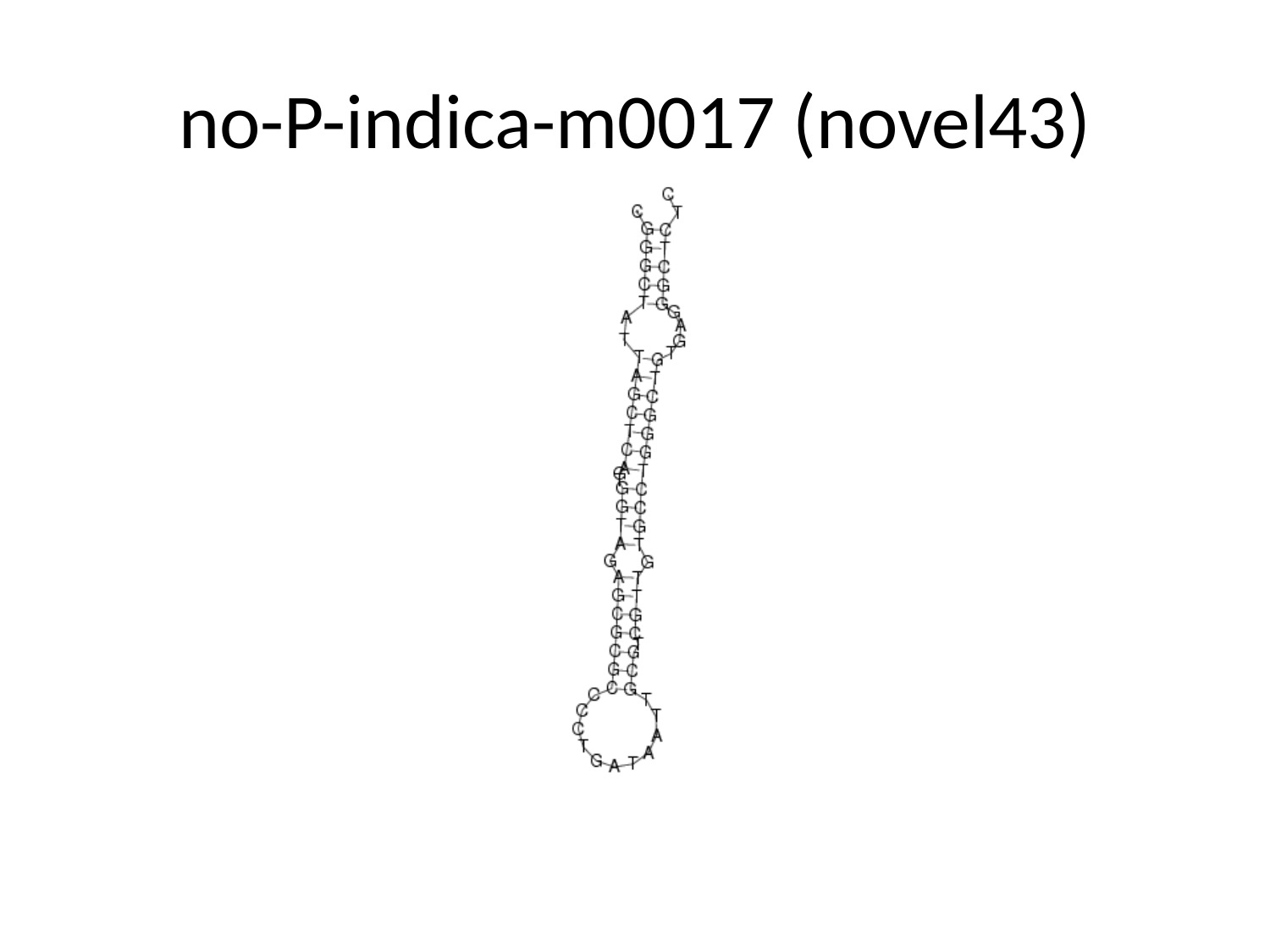

# no-P-indica-m0017 (novel43)

## Slide 50
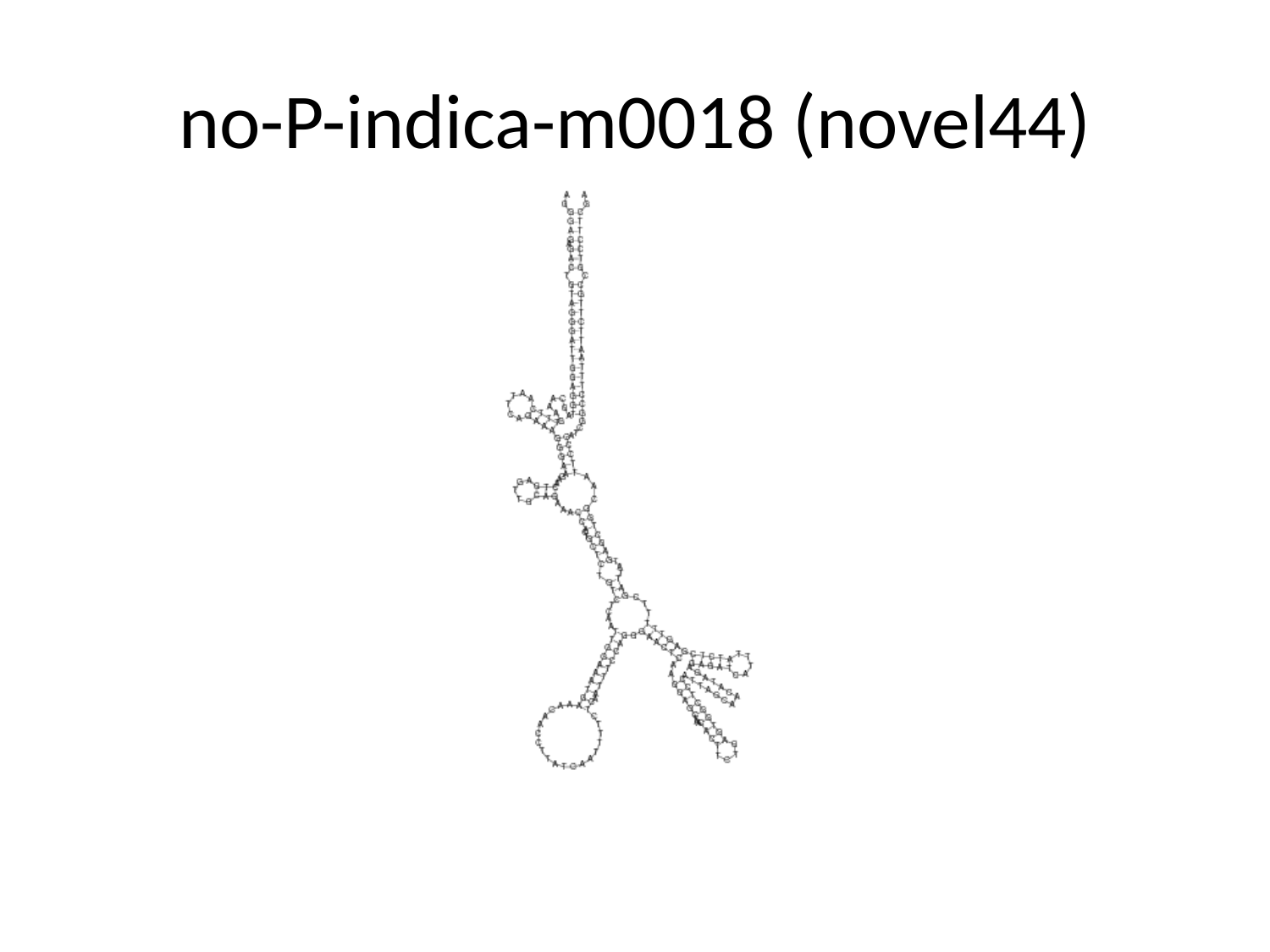

# no-P-indica-m0018 (novel44)

## Slide 51
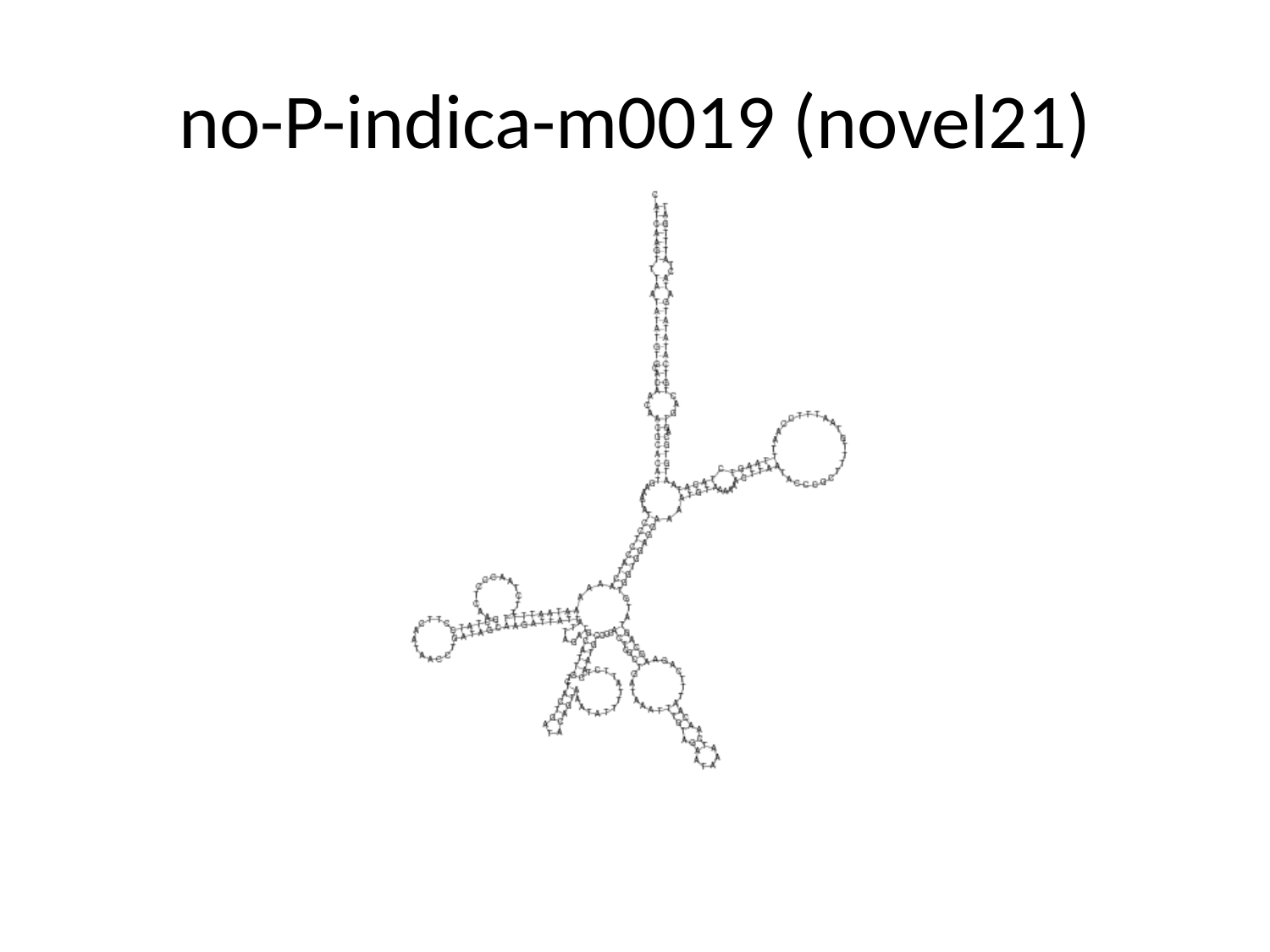

# no-P-indica-m0019 (novel21)

## Slide 52
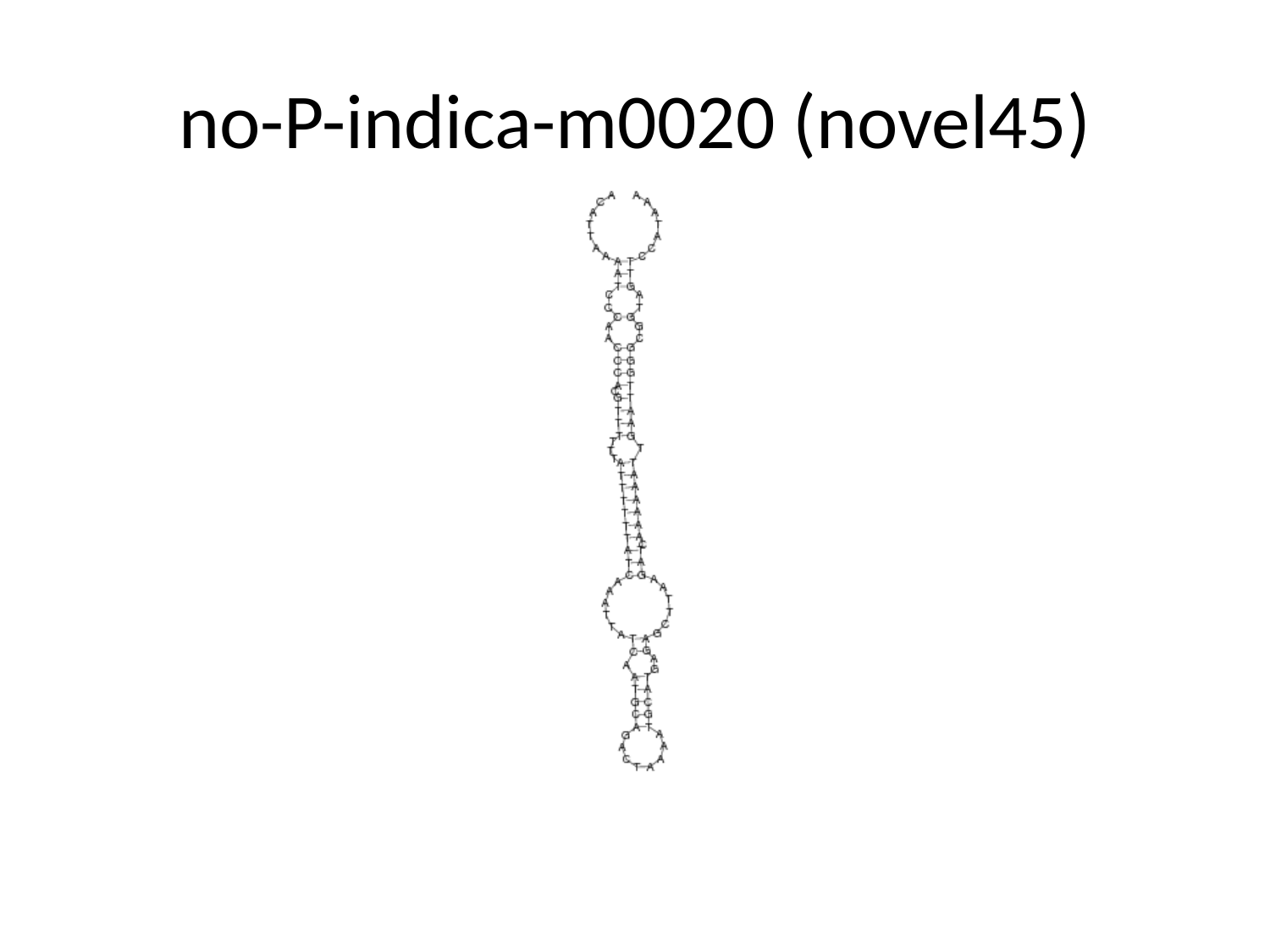

# no-P-indica-m0020 (novel45)

## Slide 53
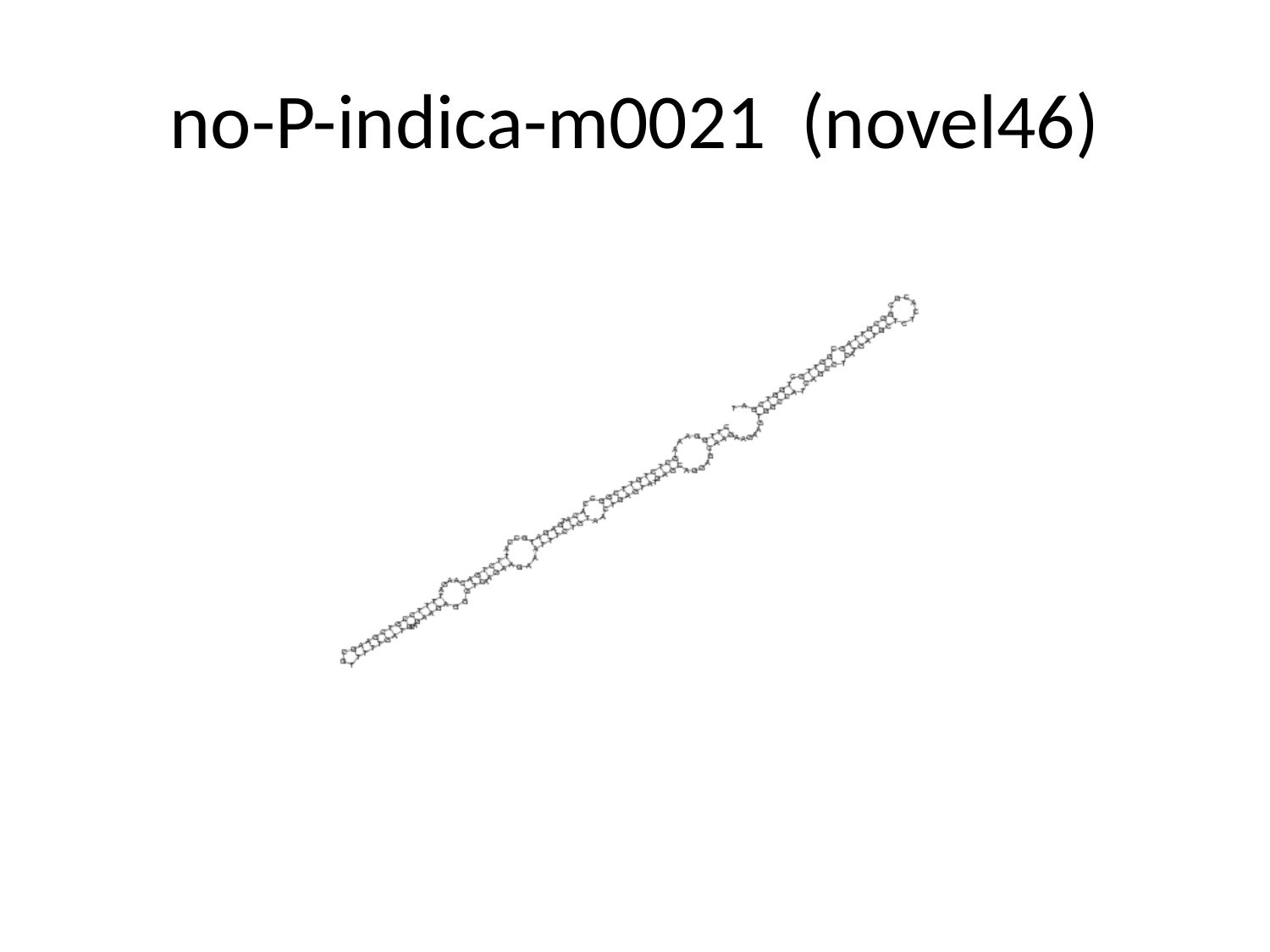

# no-P-indica-m0021 (novel46)

## Slide 54
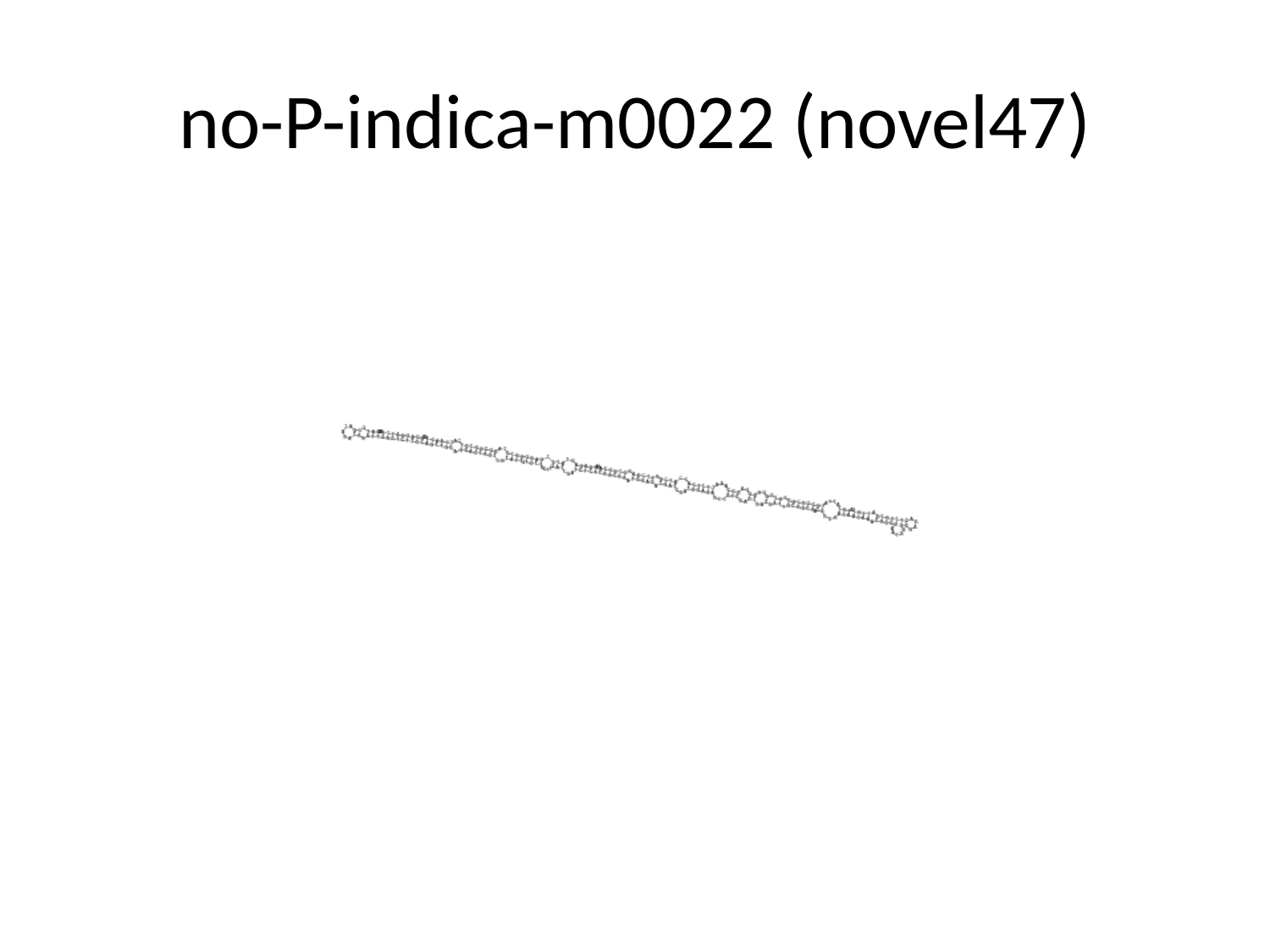

# no-P-indica-m0022 (novel47)

## Slide 55
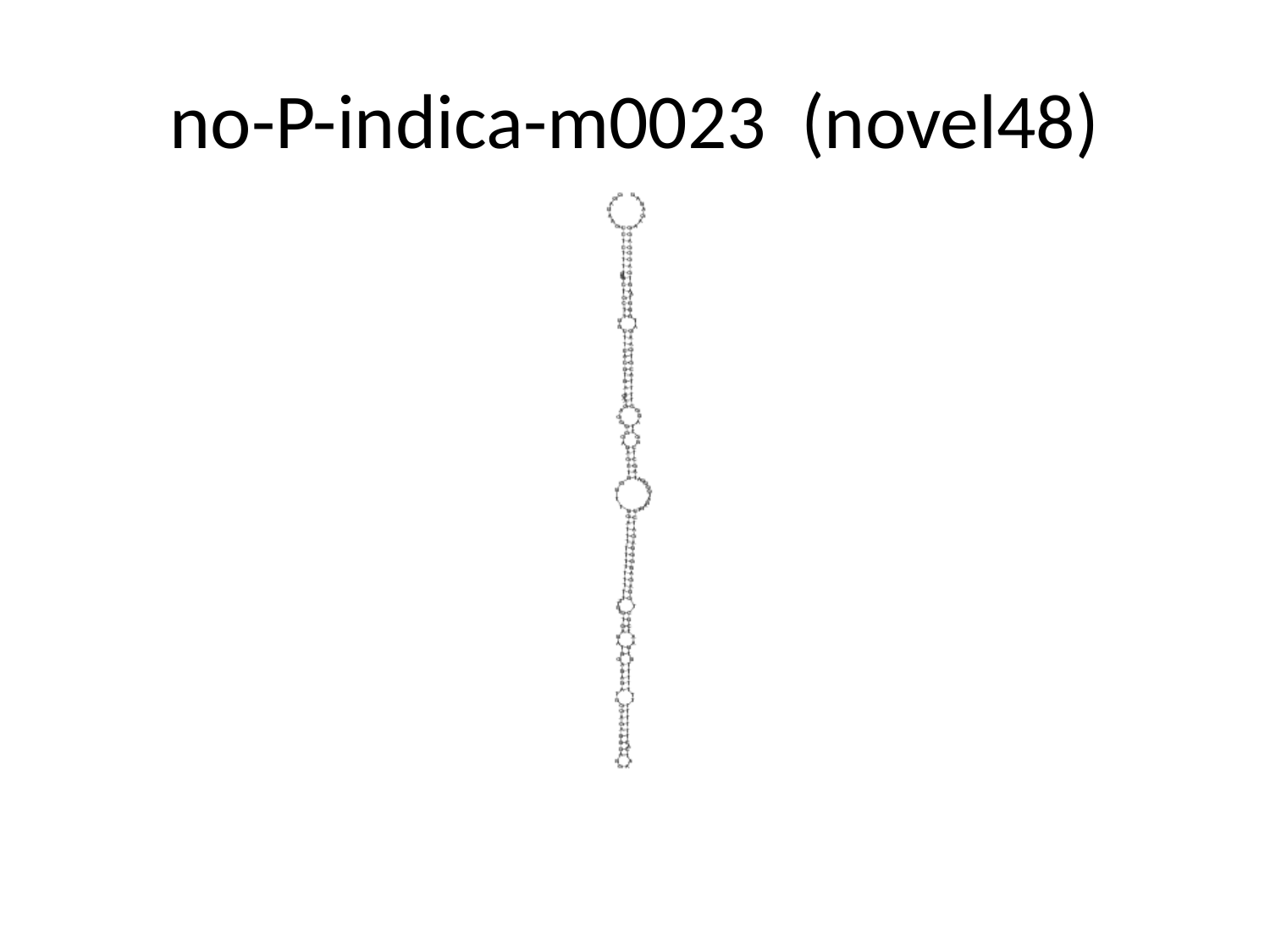

# no-P-indica-m0023 (novel48)

## Slide 56
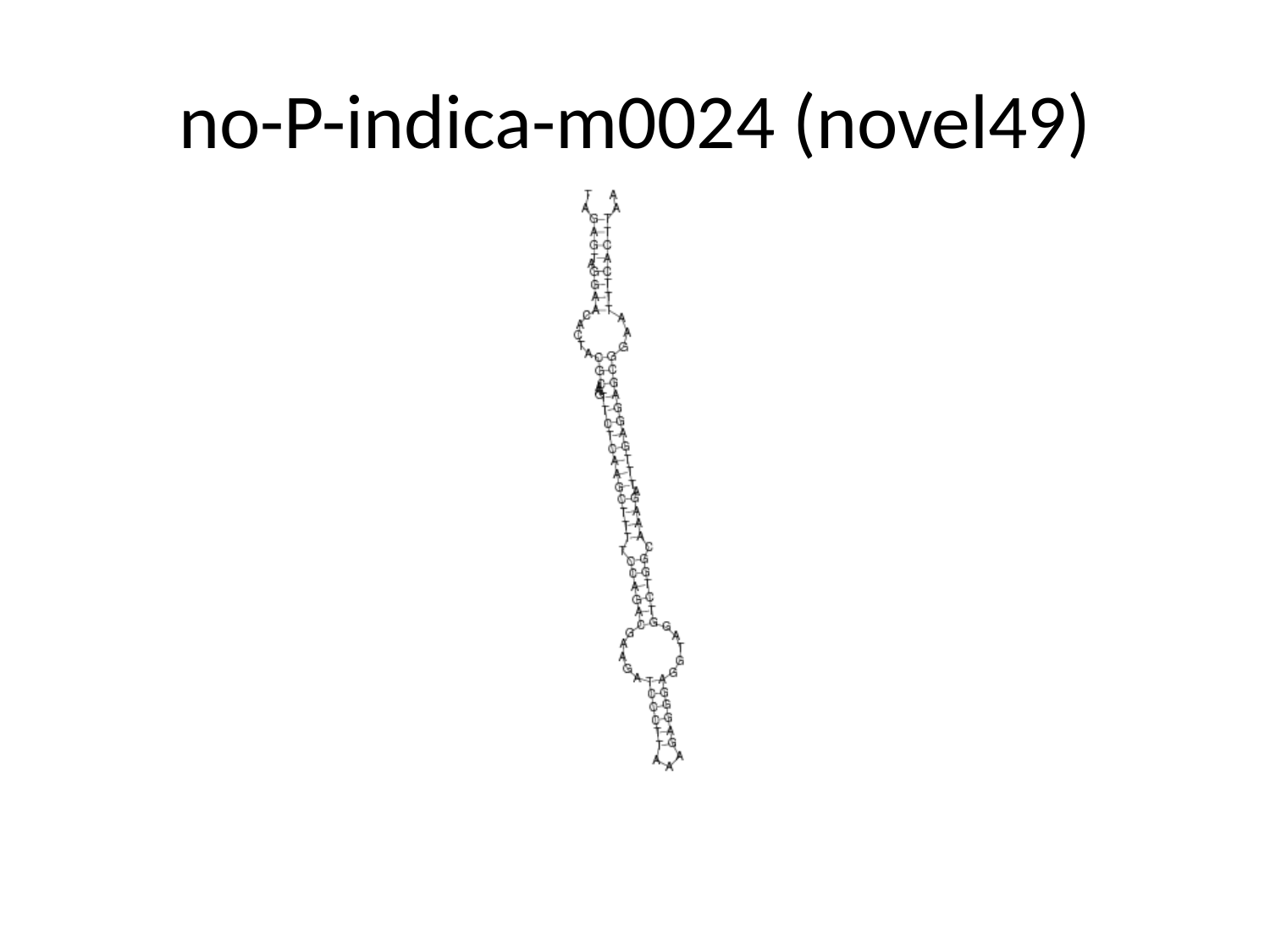

# no-P-indica-m0024 (novel49)

## Slide 57
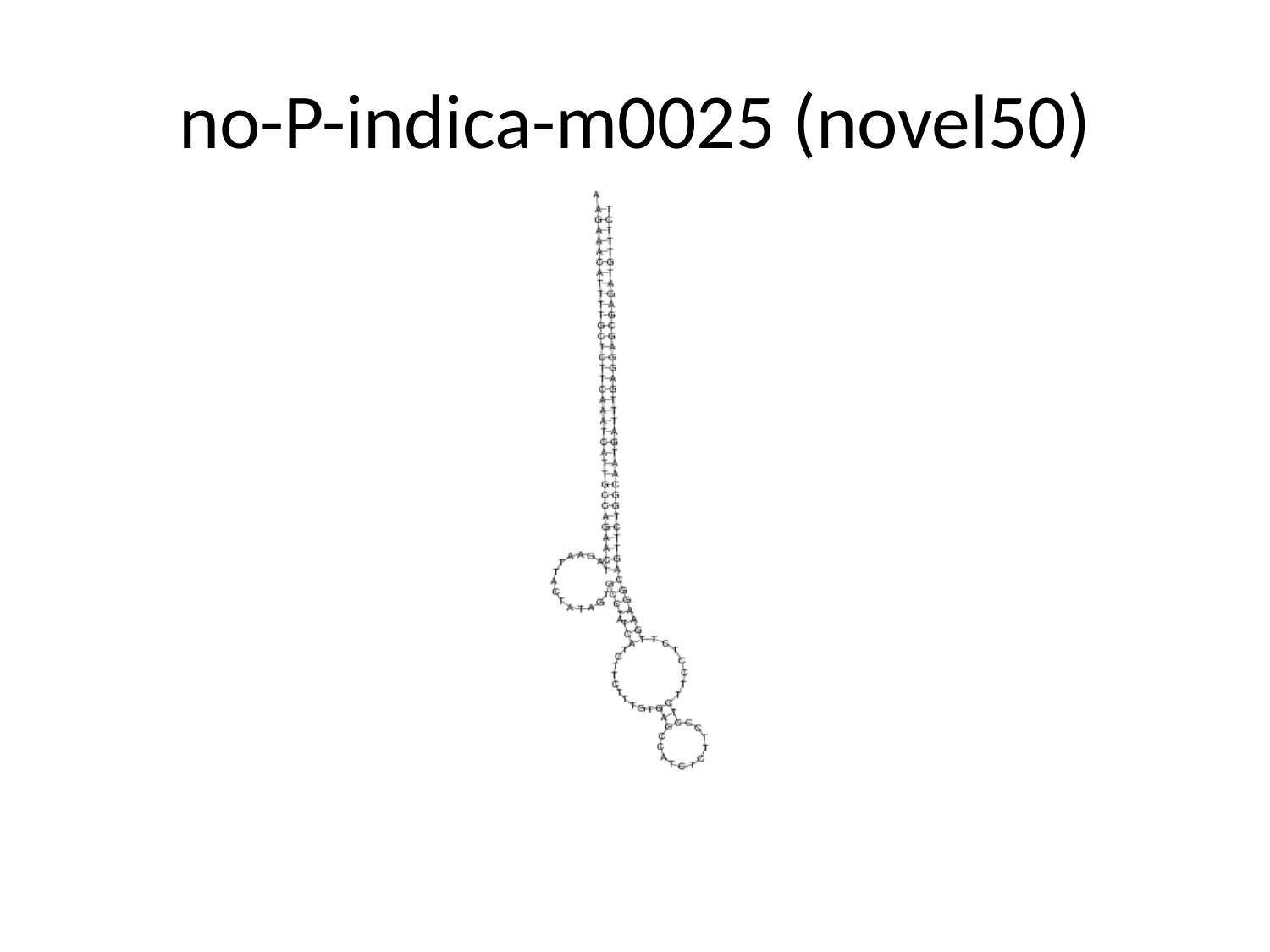

# no-P-indica-m0025 (novel50)

## Slide 58
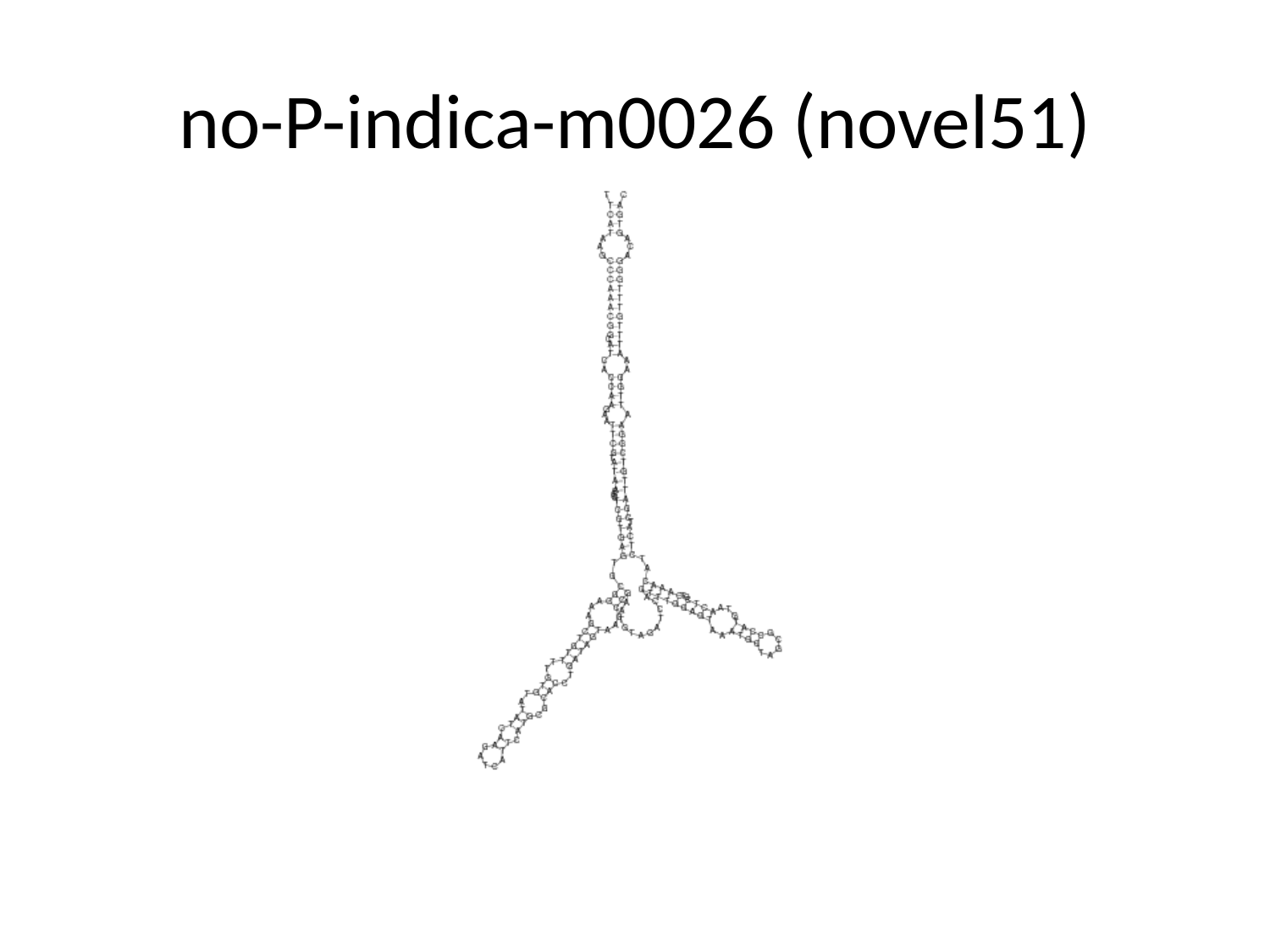

# no-P-indica-m0026 (novel51)
